# FRENCH versus ESI: comparison between two nurse triage emergency scales with referent scenarios

**DOI:** 10.1186/s12873-022-00752-z

**Published:** 2022-12-12

**Authors:** Antoine Aubrion, Romain Clanet, JP Jourdan, Christian Creveuil, E Roupie, Richard Macrez

**Affiliations:** 1 Emergency medical service (SAMU 14), Caen University Hospital, Caen, France; 2 Emergency department, Lisieux Hospital, Lisieux, France; 3 Department of emergency medicine, Caen-Normandie Hospital (CHU), Caen, France; 4 Emergency department, Bayeux Hospital, Bayeux, France; 5 Pharmacy department, Public hospital, Vire, France; 6 Department of Biostatistics and Clinical Research, Caen University Hospital, Caen, France; 7 Physiopathology and Imaging of Neurological Disorders, Normandie Univ, UNICAEN, INSERM, UMR-S U1237, Institut Blood and Brain @ CaenNormandie, GIP Cyceron, Boulevard Becquerel, 14074, Caen, France; 8 Normandie Univ, Unicaen, Cermn, 14000 Caen, France

**Keywords:** Emergency medicine classification, Emergency nursing, Triage, FRENCH scale, ESI

## Abstract

**Objectives:**

Acute triage is needed to prioritize care and achieve optimal resource allocation in busy emergency departments. The main objective is to compare the FRench Emergency Nurse Classification in Hospital scale (FRENCH) to the American scale Emergency Severity Index (ESI). Secondary objectives are to compare for each scale the over and under-triage, the triage matching to the gold standard and the inter-individual sorting reproducibility between the nurses.

**Methods:**

This is a prospective observational study conducting among the nursing staffs and nursing students, selected from Caen University College Hospital and Lisieux Hospital Center emergency departments between two months. Each group individually rank 60 referent clinical cases composed by scales designers. An assessment of scale practicality is collected after for each tool. The collected parameters are analyzed by a Cohen kappa concordance test (κ).

**Results:**

With 8151 triage results of gold standard scenarios sorting in two scales by the same nurses, the FRENCH scale seems to give better triage results than the US ESI scale (nurse: FRENCH 60% and ESI 53%, *p* = 0.003 ; nursing students: FRENCH 49% and ESI 42%, *p* < 0.001). In the two groups ESI has also a big tendency to under-sort (*p* = 0.01), particularly for the most severe patients (*p* < 0.01). The interobserver sorting concordance for any experience gives good results for the FRENCH and the ESI without any difference (nurses : FRENCH K_PQ_=0.72 ESI K_PQ_=0.78; *p* = 0.32 ; students K_PQ_=0.44 K_PQ_=0.55; *p* = 0.22).

**Conclusion:**

The ESI and FRENCH scales comparison on 8151 sorting results shows direct validity in favor of FRENCH one and similar interobserver agreement for both scales.

**Supplementary Information:**

The online version contains supplementary material available at 10.1186/s12873-022-00752-z.

## Introduction

Acute triage is needed to prioritize care and achieve optimal resource allocation in busy emergency departments. Triage is an old process created during the Napoleonic battlefields, then was developed in the civil sector at the beginning of the XXth century [1]. Triage is a key process in emergency departments (ED) organization consisting to decide patient issue by trying to manage patient care and system efficiency [2]. A systematic triage of patients using approved tools is recommended by national and international societies of emergency medicine [3, 4].

Several scales have been developed as decision supports to guide nurses in triage decision. Australasian Triage Scale (ATS, Australia) is based on clinical features in relation to patient presentations [5]. Manchester’s triage system (MTS, United Kingdom) uses series of general and specific determinants to guide decision-making, with patient presentation algorithms [6]. Canadian scale (CTAS, Canada) is based on ATS but includes diagnosis as well [7]. Emergency Severity Index (ESI, USA) published by the Agency for Healthcare Research and Quality in 2005 excludes immediate vital risk and serious disease before considering necessary estimated care [8]. Finally, French Emergency Nurse Classification in Hospital scale (FRENCH, France) is based on 100 determinants (complaints, signs, and vital parameters) [9]. ESI and FRENCH seem to have the best results for appliance and validity. All these scales are based on expert opinion [10]. Using them in ED needs to precisely identify their role and the expected objectives, with a particular attention to their validity and reliability [11]. Under-triage compromises patient’s health and over triage consumed medical team and resources [12]. Triage quality and triage-nurse experience are not always linked [13].

There is no gold standard to evaluate these scales. Evaluating a triage scale with real cases is similar to assessing the way a nurse team can sort patients: it can’t independently assess the tool itself [9]. Undirect validity criteria like consumption resources prediction or hospitalization are considered as validity standards [14]. Validity represents the accuracy of the triage scale, but is not an evaluation of patient correct triage. Using patients don’t allow to compare to gold standard sorting. Before implementation on real patients, a tool should first prove its validity on its own test cases that it proposes. Furthermore direct validity is not influenced by the scale but can only be performed on paper cases.

Our main objective was to compare the French and ESI scales direct validity, by correct response rate for each scale, over and under-triage rates on its example scenarios. Secondary objectives were to compare for each scale the over and under-triage, the triage matching to the gold standard and the inter-individual sorting reproducibility between the nurses.

## Methods

This is a prospective observational study conducting among the nursing staffs and nursing students, selected from Caen University College Hospital and Lisieux Hospital Center emergency departments. For a situation on a real patient, no referral triage score is available. The reliability can only be evaluated on the homogeneity of the answers given by the interobserver agreement. There is a necessity to rely on “gold standard” or “referent” scripted scenarios to allow for comparative assessments during training, as opposed to unpredictable and uncontrolled presentation of actual patients in the ED. The scenarios composed by the designers of each scale provide the reference response expected by the experts. This answer defines the “gold standard” sort for each paper scenario, and allows to define the correct answers, over and under-sorting.

We designed two groups: a first group with graduated nurses with at least 5 years of experience working daily in the ED, and triage without formalized scale(GN) and a second one composed by second year nursing students (NS) but without prior experience working in ED or patient sorting. An interval of 15 days between the two tests for each nurse, and a reversal of the order of the tests for each half group was organized. Each nurse received triage training for each scale before working on a support including two parts: a scale presentation of five pages, 60 referent clinical cases, composed by the designer’s scales with a gold standard triage score. Each nursehad individually rank these 60 same referent clinical cases in 30 min, with the scale handbook available. Then, they assessed the practicality of each scale.

These scenarios were selected from scale handbooks. For the ESI scale, the presentation and the clinical cases have been translated from the ESI Handbook v.4 into French. Two physicians fluent in English and French reviewed the translation. Some vital constants have been converted into European units rounded to the tenth (weight in pounds to kilograms and temperature in Fahrenheit degrees to Celsius). The French version has been adapted nearest as possible of American one.

Statistical analysis were performed with a concordance Cohen’s kappa test with linear weighting (κPL) and quadratic weighting (κPQ). The kappa values interpretation results was based on the definitions provided by Altman and Viera. (0-0.2 poor, 0.2–0.4 passable, 0.4–0.6 moderate, 0.6–0.8 good, 0.8-1 very good) [15, 16]. Their comparison was accomplished by a bilateral Student test. The analysis was made at the Unit of Biostatistics and Clinical Research of the Caen University College Hospital with the IBM SPSS and R software programs. Statistical significance was defined as *p* < 0.05.

## Results

In the first group, sixteen graduated nurses (69.6%) accepted to participate to the study, constituting GN group. Among the students, 69 (90.0%) agreed to answer the test, constituting the NS group. 3 students were excluded by a very low response rate (< 10%).

In GN group, based on 1920 cases sorted in a scale (response rate 100%), we obtained for the FRENCH scale 575 correct triage (59.9%) for FRENCH and 508 (52.9%) for ESI (*p* = 0.033; Table 1 A). Over-triage was more commonfor FRENCH (22.5%) compared to ESI (11.5%), *p* < 0.01. Under-triage was less common for FRENCH (17.6%) compared to ESI (35.6%; *p* < 0.01, Table 1 A). In comparison, for level 1, the same users have correctly sorted 63 cases out of 80 (78.8%) with the French, against 109 out of 192 (56.8%) with the ESI (*p* < 0.001). For level 2, 64.8% with the French (83/128), against 42.9% with the ESI (96/224) ; *p* < 0.001 (Table 1 A). Categories 1 and 2 were more under-sorted in ESI (level 1: 43.2%, level 2: 50.4%, *p* < 0.001, Table 1 A) compared to FRENCH scale (level 1: 21.3%, level 2: 17.2%; *p* < 0.001, Table 1 A). For concordance test compared to the gold standard sorting, we obtained an average of κPL = 0.63 and κPQ = 0.77 for the FRENCH; similar to average of κPL = 0.63 and κPQ = 0.78 for ESI (Table 1B). For inter-observer triage concordance we obtained κPL = 0.52 and κPQ = 0.69 for FRENCH and κPL = 0.58 and κPQ = 0.75 for ESI without any significative difference (p-κPL = 0.34; p-κPQ = 0.34; Table 1 C).

In the NS group, based on the 6231 cases sorted in a scale (response rate 78% [IC 74–82]), the same users have correctly sorted 1515 cases out of 3101 (48.8%) with the FRENCH scale, against 1327 out of 3130 (42.4%) with the ESI (*p* < 0.001; Table 2 A). Over-triage was more common for FRENCH (33.7%) compared to ESI (21.8%; *p* < 0.001). Under-triage was less common for FRENCH (17.4%) compared to ESI (35.8%; *p* < 0.001). In comparison, for level 1, the same users have correctly sorted 147 cases out of 268 (54.9%) with the French, against 242 out of 650 (37.2%) with the ESI (*p* < 0.01). For level 2, 50.6% with the French (220/435), against 42.9% with the ESI (325/757) ; *p* = 0.02. Categories 1 and 2 were more under-sorted in ESI (level 1: 62.8%, level 2: 48.6%) compared to FRENCH scale (level 1: 45.1%, level 2: 25.3%; *p* < 0.01 and *p* < 0.01. For concordance test compared to the gold standard sorting, we obtained an average of κPL = 0.45 and κPQ = 0.58 for the FRENCH compared to κPL = 0.49 and κPQ = 0.66 for ESI (Table 1B). For inter-observer triage concordance we obtained κPL = 0,31 and κPQ = 0.44 for FRENCH and ΚPL = 0,41 and κPQ = 0.55 for ESI without any significant difference for nursing students (p-κPL = 0.18 ; p-κPQ = 0.22; Table 2C).

Both groups (GN and NS) found significatively more practical FRENCH scale compared to ESI (Table 3).

**Table 1:**
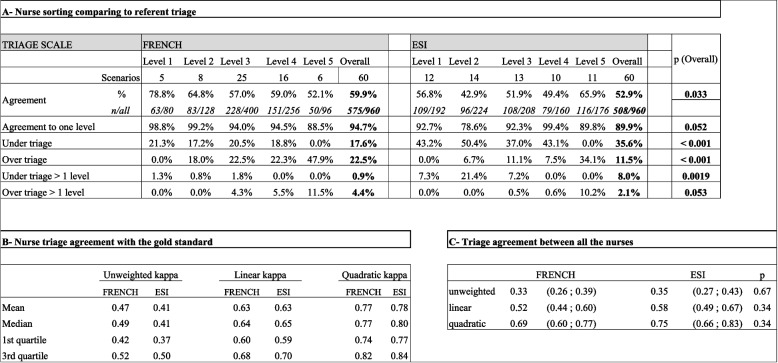
Triage results with graduated nurses group (GN)

**Table 2:**
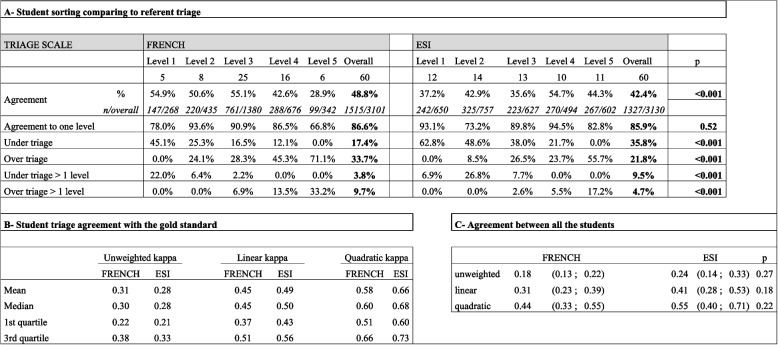
Triage results with nurses student group (NS)

**Table 3:** Users scale practicality evaluation about FRENCH and ESI

	Nurses				Students			
Question	FRENCH	ESI	IC 95%	*p*	FRENCH	ESI	IC 95%	*p*
Ease of learning	8.83	6.29	[1.78 ; 5.22]	*p* < 0.01	7.81	6.81	[0.18 ; 0.82]	*p* = 0.017
Ease of use	8.00	5.37	[2.05 ; 4.95]	*p* < 0.01	7.71	5.94	[1.00 ; 2.54]	*p* < 0.001
Speed of implementation	4.50	5.33	[-1.71;1.38]	*p* = 0.793	7.23	6.94	[-0.56 ; 1.14]	*p* = 0.498
Safety of sorting	7.83	4.67	[3.25 ; 5.42]	*p* = 0.039	7.87	4.45	[2.73 ; 4.10]	*p* < 0.001
Sense of use of the scale	8.66	4.20	[3.25 ; 5.42]	*p* < 0.001	7.81	5.29	[1.85 ; 3.18]	*p* < 0.001
Correlation with the clinical feeling of the situation	7.67	6.04	[-0.07 ; 4.41]	*p* = 0.055	7.32	4.55	[2.18 ; 3.37]	*p* < 0.001

## Discussion

With French GN or SN, we showed that FRENCH scale gives better triage results than the US ESI scale, whatever nurses experience. For unstable patients, we observed that ESI scale is less performing : more undertriage could potentially lead to adverse outcomes and supports the use of FRENCHThe inter-observer triage concordance for any experience gives no significant difference with 8100 results of dual triage. Moreover, the practicality of the two French user populations are in favor of the FRENCH scale about learning, use facility, and tool security.

We chose to take up the 60-referent cases made by each of the two sorting scales developers; their rating defined a gold standard triage for these fictional patients. The same nurses have sorted the cases of the two scales, after training, but without previous experience of one or the other of the two scales. These clinical situations on paper make it possible to guarantee identical triage conditions: each nurse is confronted with the same cases, without neither the subjectivity induced towards the patient, the simultaneous sorting with two judges’ blind, nor the sorting bias posteriori. This is independently assessed of their triage experience and their service habits [9]. These results are consistent with other studies on these same ESI (κPL = 0.84, IC: 0.77–0.91) [17].

For the inter-observer concordance test we obtained correct results with two scales and our data are in accordance to the literature (FRENCH κPL = 0.77, κ = 0.64, ESI v.3 κPL = 0.89) [9, 18]. These concordances are lower than in referring articles with a common practice of the evaluated scale for nurses [9]. In order to compare their results, our nurses did not know either of the two scales in common practice. We have not compared the results of nurses with similar experience but with all experience. Both concordances would be artificially increased. On the other hand, in order to test better knowledge, it seems that the referent cases proposed by the developers of each scale are more difficult to sort than the average of real cases. Finally, triage of paper cases usually gives a lower agreement than the identical real cases [19].

This study has several limits. Paper scenarios obtain different results of triage compared to real cases. However it allows a better inter-individual comparability of the triage. However, paper-cases may not be representative of real clinical practice in ED and leave room for imagination. Cases simulated by an actor would not have this limitation. Furthermore, as the clinical scenarios were performed differently for the two scales, the differences observed may be due to differences in the difficulty of the scenarios (level 1–2 scenarios: 13/60 for French and 26/60 for ESI). Using the same scenarios, by consensus of experts on both scales would not have such important limitation. However, the evaluation of the clinical cases by the experts who constructed each scale seemed more robust than a comparative evaluation by independent experts. The same raters participated in each scale test. Despite an interval of 15 days between the two tests for each nurse, and a reversal of the order of the tests for each half group, contamination between the two scales was indeed possible (Additional file 1: Figure S1). As the difference in choice between two judges is important for the same scale, it seemed important to use the same judges in each scale in order to be able to interpret the results of correct sorting in one or other of the scales [2]. In the case of a randomized trial, the comparison of kappa cannot distinguish the influence of the scales and those of the different judges in the two groups, because of a low intrinsic kappa for the scale.

Experienced nurses worked in the same emergency department but each one had previous experience in other department. The two populations effectives are different but represents a big part of each analyzed group (respectively 69.6% of experience nurses and 90.0% of the nurse students). For both scales, the training received by learners was short. The time offered to answer the questionnaires was limited. The response rate differs between the two groups but is related to the speed of implementation: unlike experienced nurses, most nursing students were unable to full complete the questionnaire. To limit the measurement bias, nurse had to respect the order of the question. A post-training test over several days could provide different results. Subjectivity bias is limited by retaining the same nurses for both scales. Even if the greatest care has been taken for this stage, the translation of the scale and the paper cases of ESI into FRENCH may have lost some nuances. Selection bias is limited by the absence of sampling since all existing scenarios for each scale were included.

Our results seem to be in favor of the FRENCH scale. Indeed each scale was developed in a given context and for a given care organization. Although naive on each scale and in spite of training identical to each one, they may have been influenced by their training,education, and organization French models. The French model defines the nurse as an effector of the medical decision. Whereas the American model leaves a more important place to the decision-making of the nurse, American nurses are more trained and better qualified than in our European system. Thus, for our French nurses, the FRENCH scale may appear more adapted to their practice and more secure. In fact, FRENCH scale leave less freedom to the nurses compared to the ESISo, in a French-style health care system, French nurses and nursing students seem more prone to apply according to the way it has been elaborated, the FRENCH scale than the American ESI scale.

Our study compares two cultures and two ways of thinking. The ESI leaves more freedom for the nurses judgment coupled with an assessment of care needed. FRENCH headed scale approaches an e-sorting scale. A recent paper evaluates an electronic triage system (e-triage) based on machine learning that predicts likelihood of acute outcomes enabling improved patient differentiation [20]. As the FRENCH scale, e-triage is composed of a random model applied to triage data (vital signs, chief complaint) to determine a triage score and seems to improve ESI under-sorting. In both cases the final adjustment of the triage score, depending on the clinical context and the patient’s medical history, is based on the evaluator’s experience: triage nurse for FRENCH and big data in e-sorting. In view of their similar results with ESI, these two conceptions of final triage fit still need to be compared together in a prospective study.

However in the absence of gold standard scale, any comparison need to be attentive to evaluation criteria. In fact the ESI takes into account the care to be provided and therefore influences the indirect validity. Thus an e-sorting scale will have good concordance results. The direct validity is not influenced by the scale but can only be performed on paper cases. However gold standard scenarios are necessarily developed by the designers of the scale. A lower difficulty of the scenarios can enable them to obtain better scores of adequate sorting (direct validity). Identical scenarios for each scale, sorted by their own experts, would limit this bias.

## Conclusion

Triage is an old process required in any emergency department but without gold standard scale. Different scales are proposed according to countries and cultures. The comparison of two scales requires evaluators without previous experience of one or the other of the two scales and gold standard scenarios for each scale. The ESI and FRENCH comparison on referentpapercase with experience and student French nurses showed direct validity in favor of FRENCH and similar inter-observer agreement for both scales. Triage with these scales appears easily applicable and reproducible and will improve our practices in caring from the emergencies’ reception. Further studies are needed, especially to evaluate the effectiveness of guided e-sorting scales.

## Supplementary Information

**Additional file 1: Figure S1**. Study diagram.
**Additional file 2**.

## Data Availability

The datasets used and/or analysed during the current study available from the corresponding author on reasonable request.

## Abbreviations

ED: Emergency Department
ESI: Emergency Severity Index
FRENCH: FRench Emergency Nurse Classification in Hospital scale
GN: Graduated Nurses group
NS: Nurses Student group
κPL: kappa test with linear weighting
κPQ: kappa test with quadratic weighting

## Declarations

Our study proposes the evaluation by professional nurses or apprentices of clinical situations on paper proposed in the learning guide of the Emergency Severity Index (ESI), and those of the French Emergency Nurse Classification in Hospital Scale (FRENCH). These practice cases are the official tests that give the correct triage for each situation. They are considered gold standard cases for testing the validity of the sorting in each scale.

The evaluation of a triage scale with real patients would not allow to compare the 2 scales between them. It would be similar to evaluating how well a team of nurses can triage patients. It cannot independently evaluate the tool itself. As indicated in the bibliography, direct validity can only be achieved by using the paper-based situations proposed in the learning guide for each scale.

All participating nurses were informed about the objectives of the survey. Their written consent was obtained before filling in the questionnaire. The clinical situations used were those of the official national guides. No real patients or medical data were used in this study.
