# Supplementary material for: FRENCH versus ESI: comparison between two nurse triage emergency scales with referent scenarios
Source: BMC Emerg Med. 2022 Dec 12;22:201. doi: 10.1186/s12873-022-00752-z (PMC9743579; doi:10.1186/s12873-022-00752-z)
Supplement: Supplementary file 1 — Additional file 1: Figure S1. Study diagram. Additional file 2. [file 12873_2022_752_MOESM1_ESM.pdf]

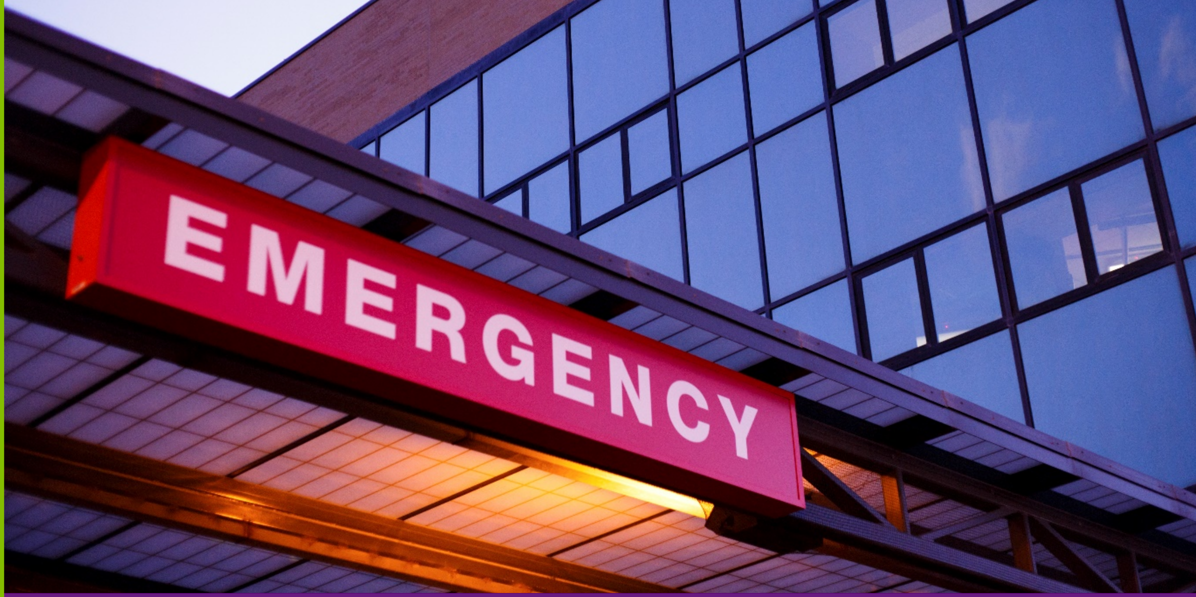

IMPLEMENTATION HANDBOOK
2020 EDITION

ESI

EMERGENCY SEVERITY INDEX

A Triage Tool for Emergency Department Care

Version 4

EMERGENCY NURSES ASSOCIATION

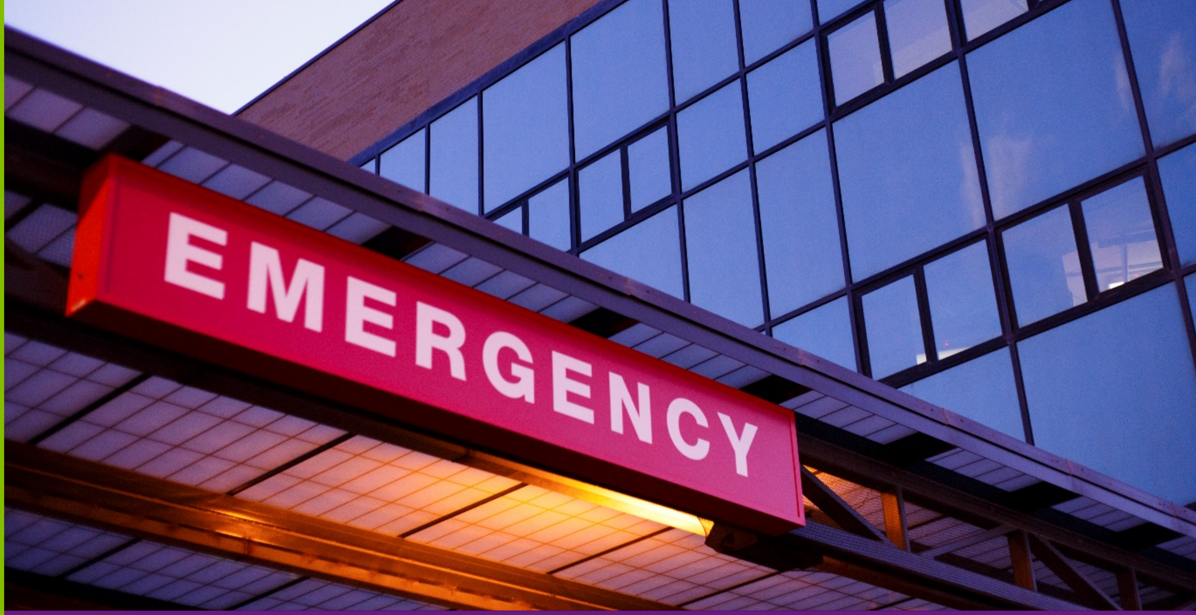

IMPLEMENTATION HANDBOOK
2020 EDITION

ESI

EMERGENCY SEVERITY INDEX

A Triage Tool for Emergency Department Care

Version 4

Nicki Gilboy RN, MS, CEN, FAEN
Associate Chief Nursing Officer for Emergency Medicine
UMass Memorial Medical Center
Worcester, MA

Paula Tanabe, PhD, MSN, MPH, RN
Associate Professor
Schools of Nursing and Medicine
Duke University
Durham, NC

Debbie Travers, PhD, RN, FAEN, CEN
Assistant Professor, Health Care Systems and Emergency Medicine
Schools of Nursing and Medicine
University of North Carolina
Chapel Hill, NC

Alexander M. Rosenau, DO, CPE, FACEP
Senior Vice Chair, Department of Emergency Medicine
Lehigh Valley Health Network, Allentown, PA
Associate Professor of Medicine
University of South Florida, Tampa, FL
Co-Medical Director, Eastern EMS Council

EMERGENCY NURSES ASSOCIATION

EMERGENCY NURSES
ASSOCIATION
Emergency Nurses Association
930 E. Woodfield Road
Schaumburg, IL 60173
847-460-4000
education@ena.org
www.ena.org

Copyright © 2020 by Emergency Nurses Association (ENA)

All rights reserved. No part of the material protected by this copyright may be reproduced or utilized in any form, electronic or mechanical, including photocopying, recording, or by any information storage and retrieval system, without written permission from the copyright owner.

The content, statements, views, and opinions herein are the sole expression of the respective contributors. Reference herein to any specific commercial product, process, or service by trade name, trademark, manufacturer, or otherwise does not constitute or imply its endorsement or recommendation by ENA, and such reference shall not be used for advertising or product endorsement purposes. All trademarks displayed are the trademarks of the parties noted herein. ESI Implementation Handbook is an independent publication and has not been authorized, sponsored, or otherwise approved by the owners of the trademarks or service marks referenced in this product.

There are images in this book that feature models; these models do not necessarily endorse, represent, or participate in the activities represented in the images. Any screenshots in this product are for educational and instructive purposes only. Any individuals and scenarios featured in the case studies throughout this product may be real or fictitious but are used for instructional purposes only.

ENA accepts no responsibility for course instruction by the course director or any course instructors. Because implementation of this course of instruction involves the exercise of professional judgment, ENA shall not be liable for the acts or omissions of course participants in the application of this course of instruction.

The authors, editors, and ENA have checked with reliable resources in regards to providing information that is complete and accurate. Due to continual evolution of knowledge, treatment modalities, and drug therapies, ENA cannot warranty that the information, in every aspect, is current. ENA is not responsible for any errors, omissions, or for the results obtained from use of such information. Please check with your healthcare institution regarding applicable policies.

Medication dosages are subject to change and modification. Please check with your local authority or institutional policy for the most current information.

Editorial and Production Credits
Director, Content Development: Nicole Williams, MSN, RN-BC
Senior Product Manager: Mark Kardon
eLearning Manager: Ken Loredo
Nurse Content Manager: Katrina Ceci, MSN, RN, TCRN, CPEN, NPD-BC, CEN
Nursing Education Specialist: Stephanie Klavohn, BSN, RN, CEN, TCRN
Developmental Editor: Chris Zahn, Ph.D.
Graphic Artist: Lauren Hamm
Cover Image: © Antishock/iStock/Getty Images

Printed in the United States of America
24 23 22 21 20   10 9 8 7 6 5 4 3 2 1

Contents

	Preface	**xiii**
	Acknowledgments	**ix**
**CHAPTER 1**	Introduction to the Emergency Severity Index: A Research-Based Triage Tool	1
	Standardization of Triage Acuity in the U.S.	1
	History of the Emergency Severity Index	2
	Research on the Emergency Severity Index	2
	Benefits of the Emergency Severity Index	4
	References	5
**CHAPTER 2**	Overview of the Emergency Severity Index	7
	Decision Point A: Does the Patient Require Immediate Lifesaving Intervention?	9
	Decision Point B: Should the Patient Wait?	11
	Decision Point C: Resource Needs	12
	Decision Point D: The Patient's Vital Signs	13
	Summary	15
	References	15
**CHAPTER 3**	ESI Level 2	17
	1. Is This a High-Risk Situation?	18
	2. Is the Patient Experiencing New Onset Confusion, Lethargy, or Disorientation?	22
	3. Is the Patient Experiencing Severe Pain or Distress?	22
	Special Situations	25
	Summary	25
	References	25

**CHAPTER 4**	**ESI Levels 3–5 and Expected Resource Needs**	**27**
	Resource Predictions	27
	What Constitutes a Resource?	28
	Resources in Context	29
	Summary	30
	References	30
**CHAPTER 5**	**The Role of Vital Signs in ESI Triage**	**33**
	Are Vital Signs Necessary at Triage?	33
	Using Vital Signs with ESI Triage	34
	Vital Signs and Pediatric Fever	35
	Case Examples	35
	Summary	37
	References	37
**CHAPTER 6**	**Use of the ESI for Pediatric Triage**	**39**
	Background and Research	39
	Pediatric Triage Assessment: What Is Different for Pediatric Patients?	40
	A Standardized Approach to Pediatric Triage Assessment	41
	Assigning ESI Levels for Pediatric Patients	43
	Pediatric Patient Case Studies	46
	Summary	46
	References	47
**CHAPTER 7**	**Implementation of ESI Triage**	**49**
	Decision-Making and Planning	49
	Policies and Procedures	50
	Planning ESI Education	51
	Implementation Day	55
	Post-Implementation	55
	References	55
**CHAPTER 8**	**Evaluation and Quality Improvement**	**57**
	ESI Triage Quality Indicators and Thresholds	58

	ESI Triage Data Collection	59
	Summary	61
	References	61
**CHAPTER 9**	**ESI Practice Cases**	**63**
	Practice Cases	63
	Practice Cases: Answers and Discussion	70
**CHAPTER 10**	**Competency Cases**	**77**
	Set A Competency Cases	77
	Set B Competency Cases	79
	Set A Competency Cases – Answers	80
	Set B Competency Cases – Answers	82
**APPENDIX A**	**Frequently Asked Questions and Post-Quiz Materials for Chapters 2–8**	**85**
	Chapter 2	85
	Chapter 3	85
	Chapter 4	88
	Chapter 5	90
	Chapter 6	92
	Chapter 7	93
	Chapter 8	93
**APPENDIX B**	**ESI Triage Algorithm, v4**	95
**APPENDIX C**	**Initialisms, Abbreviations, and Acronyms**	97
	Index	**99**

Preface

The Emergency Severity Index (ESI) is a tool for use in emergency department (ED) triage. The ESI triage algorithm yields rapid, reproducible, and clinically relevant stratification of patients into five groups, from level 1 (most urgent) to level 5 (least urgent). The ESI provides a method for categorizing ED patients by both acuity and resource needs.

Emergency physicians Richard Wuerz and David Eitel developed the original ESI concept in 1998. After pilot testing of the ESI yielded promising results, they brought together a number of emergency professionals interested in triage and the further refinement of the algorithm. The ESI Triage Group included emergency nursing and medical clinicians, managers, educators, and researchers. The ESI was initially implemented in two university teaching hospitals in 1999 and then refined and implemented in five additional hospitals in 2000. The tool was further refined based on feedback from the seven sites. Many research studies have been conducted to evaluate the reliability, validity, and ease of use of the ESI.

One of the ESI Triage Group's primary goals was to publish a handbook to assist emergency nurses and physicians with implementation of the ESI. The group agreed that this was crucial to preserving the reliability and validity of the tool. A draft of this handbook was in progress in 2000 when Dr. Wuerz died suddenly and unexpectedly. The remaining group members were committed to the value of ESI and carrying out Dr. Wuerz's vision for a scientifically sound tool that offers emergency departments a standardized approach to patient categorization at triage. The group completed the first edition of *The Emergency Severity Index (ESI) Implementation Handbook* in 2002 (published by the Emergency Nurses Association [ENA]). The group then formed The ESI Triage Research Team, LLC, and worked with the Agency for Healthcare Research and Quality, which published the second edition in 2005. This 2012 edition was significantly updated including presentation of ESI Version 4. Supporting research was added to Chapter 1, “Introduction to the Emergency Severity Index: A Research-Based Triage Tool.” Pediatric validation research led to the addition of the pediatrics chapter, Chapter 6, “Use of the ESI for Pediatric Triage.”

The present edition of the handbook was created as part of the refreshing of the ESI education materials by the new owner of the ESI, the Emergency Nurses Association. The handbook has come full circle, again being published by the Emergency Nurses Association.

The handbook is intended to be a complete resource for ESI implementation. Emergency department educators, clinicians, and managers can use this practical guide to develop and conduct an ESI educational program, implement the algorithm, and design an ongoing quality improvement program.

The Emergency Severity Index represented a major change in the way triage is practiced. Implementation of the ESI requires a serious commitment from education, management, and clinical staff. Successful implementation of this system is accomplished by committing significant resources during training and implementation. A myriad of benefits may result from a successful ESI implementation: improvements in ED operations, support for research and surveillance, and a standardized metric for benchmarking.

This handbook is intended only as a guide to using the ESI system for categorizing patients at triage in ED settings. Nurses who participate in an ESI educational program are expected to be experienced triage nurses and/or to have attended a separate, comprehensive triage educational program. This handbook is not a comprehensive triage educational program. The ESI educational materials in this handbook are best used in conjunction with a triage educational program. Triage nurses also need education in institution-specific triage policies and protocols. For example, hospitals may develop policies regarding which types of patients can be triaged to fast-track. Triage protocols may also be developed, such as giving acetaminophen for fever, or ordering ankle films for patients who meet specified criteria.

Acknowledgments

Contributors

Cathleen Carlen-Lindauer, RN, MSN, CEN
Clinical Nurse Specialist
Department of Emergency Medicine
Johns Hopkins Hospital
Baltimore, MD
Formerly from The Lehigh Valley Hospital and Health Network
Allentown, PA

Susan McDaniel Hohenhaus, MA, RN, CEN, FAEN
Executive Director
Emergency Nurses Association and ENA Foundation
Des Plaines, IL
Formerly of Hohenhaus & Associates, Inc.
Wellsboro, PA and Chicago, IL

David Eitel, MD, MBA
Physician Advisor, Case Management
Wellspan Health System
York, PA

Jessica Katznelson, MD
Assistant Professor
Division of Pediatric Emergency Medicine
School of Medicine
Department of Pediatrics
University of North Carolina at Chapel Hill

Nancy Mecham, APRN, FNP, CEN
Clinical Nurse Specialist
Emergency Department and Rapid Treatment Unit
Primary Children's Medical Center
Salt Lake City, UT

Valerie Rupp, RN, MSN, CRNP
Adult Care Nurse Practitioner
Lehigh Valley Health Network
Allentown, Pennsylvania

Anna Waller, ScD
Associate Professor
Department of Emergency Medicine
School of Medicine
University of North Carolina at Chapel Hill

Richard Wuerz, MD (deceased)
Attending Physician
Associate Research Director
Department of Emergency Medicine
Brigham and Women's Hospital
Boston, MA
Assistant Professor of Medicine (Emergency Medicine)
Harvard Medical School
Boston, MA

1

Introduction to the Emergency Severity Index: A Research-Based Triage Tool

The purpose of triage in the emergency department (ED) is to prioritize incoming patients and to identify those who cannot wait to be seen. The triage nurse performs a brief, focused assessment and assigns the patient a triage acuity level, which is a proxy measure of how long an individual patient can safely wait for a medical screening examination and treatment. In 2008 there were 123.8 million visits to U.S. emergency departments (Centers for Disease Control and Prevention, 2008, Tables 1, 4). Of those visits, only 18% of patients were seen within 15 minutes, leaving the majority of patients waiting in the waiting room.

This chapter presents evidence for the utility of a standardized triage tool, the Emergency Severity Index (ESI).

Standardization of Triage Acuity in the U.S.

The Institute of Medicine (IOM) published the landmark report, "The Future of Emergency Care in the United States," and described the worsening crisis of crowding that occurs daily in most emergency departments (Institute of Medicine, 2006). With more patients waiting longer in the waiting room, the accuracy of the triage acuity level is even more critical. Under-categorization (undertriage) leaves the patient at risk for deterioration while waiting. Over-categorization (over-triage) uses scarce resources, limiting availability of an open ED bed for another patient who may require immediate care. Rapid, accurate triage of the patient is important for successful ED operations. Triage acuity ratings are useful data that can be used to describe and benchmark the overall acuity of an individual ED's case mix. This is possible only when the ED is using a reliable and valid triage system and when every patient, regardless of mode of arrival or location of triage (i.e. at the bedside), is assigned a triage level (Welch & Davidson, 2010). By having this information, difficult and important questions such as "Which EDs see the sickest patients?" and "How does patient acuity affect ED overcrowding?" can then be answered. There is also growing interest in the establishment of standards for triage acuity and other ED data elements in the U.S. to support clinical care, ED surveillance, benchmarking, and research activities (Barthell et al., 2004; Gilboy et al., 1999; Haas et al., 2008; Handler et al., 2004; National Center for Injury Prevention and Control, 1997).

Historically, EDs in the U.S. did not use standardized triage acuity rating systems. Since 2000, there has been a trend toward standardization of triage acuity scales that have five levels (e.g., 1–resuscitation, 2–emergent, 3–urgent, 4–less urgent, 5–nonurgent). The Emergency Nurses Association (ENA) and the American College of Emergency Physicians (ACEP) formed a Joint Triage Five Level Task Force in 2002 to review the literature and make a recommendation for EDs throughout the U.S. regarding which triage system should be used. Prior to this task force work, there were a variety of triage acuity systems in use in the U.S., dominated by three-level scales (e.g., 1-emergent, 2- urgent, 3-nonurgent). The following position statement was approved in 2003 by the Board of Directors of both organizations: "ACEP and ENA believe that quality of patient care would benefit from implementing a standardized emergency department (ED) triage scale and acuity categorization process. Based on expert consensus of currently available evidence, ACEP and ENA support the adoption of a reliable, valid five-level triage scale" (American College of Emergency Physicians, 2003; Emergency Nurses Association, 2003). The task force published a second paper in 2005 and specifically recommended EDs use either the Emergency Severity Index (ESI) or Canadian Triage and Acuity Scale (CTAS) (Fernandes et al., 2005). Both ESI and CTAS have established reliability and validity. In 2010, and then again in 2017, ACEP revised the original statement: "The American College of Emergency Physicians (ACEP) and the Emergency Nurses Association (ENA) believe that the quality of patient care benefits from implementing a standardized emergency department (ED) triage scale and acuity categorization process. Based on expert consensus of currently available evidence, ACEP and ENA support the adoption of a reliable, valid five-level triage scale such as the Emergency Severity Index (ESI)" (ACEP, 2017). Following the adoption of this position statement, the number of EDs using three-level triage systems has decreased, and the number of EDs using the

triage systems has decreased, and the number of EDs using the five-level ESI triage system increased significantly (McHugh & Tanabe, 2011).

Some hospitals continue to use other triage systems. In 2009, the American Hospital Association reported the following survey data in which hospitals reported which triage system they used: ESI (57%), 3-level (25%), 4-level (10%), 5-level systems other than ESI (6%), 2-level or other triage system (1%), no triage (1%) (McHugh & Tanabe, 2011). The Centers for Disease Control and Prevention National Center for Health Statistics reports national level data regarding ED visits (Niska et al., 2010). The report now categorizes arrival acuity as five levels based on how urgently patients need to be seen by the physician or healthcare provider and includes the following categories: immediate (immediately), emergent (1–14 minutes), urgent (15–60 minutes), semi-urgent (1 to 2 hours), and nonurgent (2–24 hours). While this time-based categorization system has not been validated, it provides national-level data of acuity case mix upon presentation.

History of the Emergency Severity Index

The ESI is a five-level triage scale developed by ED physicians Richard Wuerz and David Eitel in the U. S. (Gilboy et al., 1999; Wuerz et al., 2000). Wuerz and Eitel believed that a principal role for an emergency department triage instrument is to facilitate the prioritization of patients based on the urgency of treatment for the patients' conditions. The triage nurse determines priority by posing the question, "Who should be seen first?" Wuerz and Eitel realized, however, that when more than one top priority patient presents at the same time, the operating question becomes, "How long can each patient safely wait?"

The ESI was developed around a new conceptual model of ED triage. In addition to asking which patient should be seen first, triage nurses use the ESI to also consider what resources are necessary to move the patient to a final disposition (admission, discharge, or transfer). The ESI retains the traditional foundation of initially evaluating patient urgency, and then seeks to maximize patient streaming: getting the right patient to the right resources at the right place and the right time.

Version 1 of the ESI was originally implemented at two university-based EDs in 1999. In 2000, the ESI was revised with input from ED clinicians to include pediatric patient triage criteria, and then version 2 was implemented in five additional hospitals (including non-university teaching and community settings). Based on feedback from nurses and physicians using the ESI at these sites, along with the best available scientific evidence, the ESI was further refined in 2001 as version 3 (Wuerz et al., 2001). Limitations in ESI levels 1 and 2 criteria were noted in version 3. Tanabe et al. (2005), conducted a prospective research study of 571 ESI level-2 patients at five hospitals. Twenty percent of level-2 patients received immediate, lifesaving interventions. The study team concluded that such patients would benefit from being classified as ESI level 1. The ESI Research Team revised ESI level 1 criteria, accordingly, resulting in ESI version 4, the most current version of the triage algorithm (Tanabe et al., 2005), which is included in this implementation handbook.

Emergency physicians and nurses in the U.S. and Canada have conducted several research studies in which the reliability and validity of the ESI have been assessed. Like the Australasian, Canadian, and United Kingdom scales, ESI triage has five levels. ESI is different in both its conceptual approach and practical application. The underlying assumption of the triage scales from Australia, Canada, and the United Kingdom is that the purpose of triage is to determine how long the patient can wait for care in the ED. Clear definitions of time to physician evaluation are an integral part of both algorithms. This represents a major difference between ESI and the CTAS and the Australasian Triage Scale (ATS). *The ESI does not define expected time intervals to physician evaluation.*

The ESI is unique in that it also, for less acute patients, requires the triage nurse to anticipate expected resource needs (e.g., diagnostic tests and procedures), in addition to assessing acuity. The process of categorizing ED patients using the ESI is described in detail in subsequent chapters. Briefly, acuity judgments are addressed first and are based on the stability of the patient's vital functions, the likelihood of an immediate life or organ threat, or a high-risk presentation. For patients determined not to be at risk of high acuity and deemed "stable," expected resource needs are addressed based on the experienced triage nurse's prediction of the resources needed to move the patient to an appropriate disposition from the ED. Resource needs can range from none to two or more; however, the triage nurse never estimates beyond two defined resources.

Research on the Emergency Severity Index

In order for a triage system to be widely adopted and used, it must have excellent reliability and validity. Researchers have focused evaluation of triage systems on these constructs. (Pedhazur & Schmelkin, 1991; Waltz et al., 1991).

Reliability

For a rating system, reliability is the consistency, or agreement, among those using a rating system. Two types of reliability pertain to ED triage acuity ratings. Inter-rater reliability is a measure of reproducibility: will two different nurses rate the same patient as at the same triage acuity level? Intra-rater reliability is an indication of whether the same nurse, conducting repeated ratings, will rate the same patient as at the same acuity level.

Validity

Validity is the accuracy of the rating system. Validity assessment indicates how well the system measures what it is intended to measure. The validity of acuity levels is an indication of whether or not, for example, the level of "non-urgent" is an accurate assessment of the lack of urgency or acuity of an ED patient's problem. Validity assessments of triage have used related measures of acuity including admission rate, resource utilization, and 6-month mortality. If many patients with low acuity triage levels are admitted to the hospital, the triage system is not valid. The same would be true for very high acuity levels. If many high acuity patients were discharged home, the triage system is not valid.

Findings

In a pilot study of ESI version 1 ratings for 493 triage encounters at two Boston hospitals in 1998, researchers found that the system was both valid and reliable (Wuerz et al., 2000). The patients were triaged simultaneously by the triage nurse using the traditional three-level scale and by the research nurse who used version 1 of the ESI. After this triage, an investigator triaged the patients again using the ESI. The investigator was blinded to the research nurse's ESI rating and used only the written triage note to make the triage decision. Triage levels were strongly associated with resources used in the ED and with outcomes such as hospitalization. Higher acuity patients (ESI levels 1 and 2) consumed more resources and were more likely to be admitted to the hospital than those with lower acuity ratings (ESI levels 4 and 5). Inter-rater reliability between the research nurse and the investigator was found to be good, with 77 percent exact agreements and 22 percent within one triage level.

The reliability of the ESI has been evaluated in several studies, using the kappa statistic to measure inter-rater reliability. The kappa statistic ranges from 0 (no agreement) to 1 (perfect agreement). At one of the two original ESI sites, a study was conducted to compare the reliability of triage ratings of a three-level scale with the ESI version 1 (Travers et al., 2002). Reliability of the three-level system (weighted kappa of 0.53) was poor while it was at an acceptable level for the five-level ESI (weighted kappa of 0.68).

In another study, researchers examined the reliability and validity of ESI version 2 during and after implementation of the system into triage practice at seven hospitals in the Northeast and Southeast. During the ESI triage education program, more than 200 triage nurses at the seven sites were asked to rate 40 case studies using the ESI (Eitel et al.,2003). The study results indicated substantial inter-rater reliability with kappa statistics ranging from 0.70 to 0.80. Three hundred eighty-six triage decisions on actual patients were also evaluated and found to have acceptable to high inter-rater reliability, with weighted kappa statistics ranging from 0.69 to 0.87. In another study at a midwestern, urban ED, researchers evaluated the reliability of the ESI version 3 for 403 actual patient triages and found a kappa of 0.89 (Tanabe et al., 2004).

Researchers have also compared inter-rater reliability of the ESI triage system with the CTAS (Worster et al., 2004). Ten Canadian nurses were randomly assigned to initial ESI version 3 training or CTAS refresher training and after completing the training rated 200 case studies with the ESI or CTAS, respectively. Both groups had excellent inter-rater reliability, with kappas of 0.89 (ESI) and 0.91 (CTAS).

The validity of the ESI has been evaluated by examination of outcomes for several thousand patients. The studies found consistent, strong relationships between the ESI and hospitalization, ED length of stay, and mortality (Eitel et al., 2003; Tanabe et al., 2004; Wuerz, 2001; Wuerz et al., 2001). The ESI also has been found to moderately correlate with physician evaluation and management codes and nursing workload measures (Travers et al., 2002). The ESI has been shown to facilitate meaningful comparisons of case mix between hospitals. A stratified random sample of 200 patients was selected from each of the seven initial ESI hospitals, and case mix was compared (Eitel et al., 2003). As expected, there was a higher percentage of high acuity patients at the tertiary care centers, compared with a higher percentage of low resource patients at the community hospitals. In a survey of nursing staff at the two original university-based EDs, responses to the implementation of the ESI were positive (Wuerz et al., 2001). The nurses reported that the ESI was easier to use and more useful in prioritizing patients for treatment than the former three-level systems in use at the two sites.

The performance of ESI in pediatric patients has also been evaluated. Travers et al. (2009) have conducted the largest evaluation of ESI in pediatric patients. Reliability was evaluated using both written case scenarios and actual patient triages at five different sites. The validity of ESI was assessed in a group of 1173 pediatric patients using hospital admission, resource consumption, and ED length of stay. Interrater reliability for written case scenarios was 0.77 and 0.57 for live triages, suggesting room for improvement in educational training of ED nurses. Validity of triage categories in pediatric patients was established with outcome measures of hospitalization, resource utilization, and ED length of stay. The outcomes from this study suggested the need for additional education of ED nurses in the area of overall pediatric triage, which led to the inclusion of a pediatric chapter (Chapter 6, "Use of the ESI for Pediatric Triage," in the ESI implementation handbook. In a separate investigation, 16 ED physicians and 17 ED nurses scored 20 pediatric written case scenarios (Durani et al., 2009). Overall inter-rater reliability was excellent (weighted kappa = . 92 for physicians and .93 for nurses).

Several studies have evaluated the performance of ESI with an elderly population. In a study of 929 patients age 65 or older

with a total of 1,087 ED visits over a 1-month period in 2004, ED resource utilization, ED length of stay, hospital admission, and 1-year survival were assessed. The ESI algorithm performed well in all areas (Baumann & Strout, 2007). In a separate investigation of 782 patients > 65 years of age, the accuracy of ESI at identifying elderly patients requiring a lifesaving intervention was investigated (Platts-Mills et al., 2010). While specificity was high (99%), sensitivity was poor (42%). This suggests further evaluation of the performance of ESI in elderly patients may be warranted.

The ESI has been translated into several languages and evaluated for reliability and validity. Good inter- and intra-rater reliability (weighted kappas of .73 and .65) was found when evaluated in the Netherlands (Storm-Versloot et al., 2009). The ESI was translated into German, and researchers found excellent inter-rater reliability (k=0.985) and good evidence for validity. Relationships were found between ESI triage levels and number of resources used, hospitalization, and death (Grossman et al, 2011). In a separate evaluation in an urban European country, evidence for validity was established by the finding of relationships between the ESI and the number of resources used and proportion of patients requiring hospital admission (Elshove-Bolk et al., 2007). van der Wulp and colleagues compared the predictive validity of the ESI and Manchester triage systems (van der Wulp et al., 2009). Both systems demonstrated good predictive ability, with ESI scoring higher. Finally, ESI and the Taiwan Triage System (TTS) were compared on validity assessed by hospitalization. ESI was better able to discriminate patient acuity and hospitalization than the TTS (Chi & Huang, 2006).

Benefits of the Emergency Severity Index

The ESI has been implemented by hospitals in different regions of the country, by university and community hospitals, and by teaching and nonteaching sites. ED clinicians, managers and researchers at those sites have identified several benefits of ESI triage over conventional three-level scales. In 2008, the National Opinion Research Center conducted a survey of 935 persons who requested ESI training materials from the Agency for Healthcare Research and Quality. Respondents were asked to rate their satisfaction with ESI as a triage tool as well as to compare ESI with other triage tools. Overall, ratings of satisfaction were high; respondents reported ESI was simple to use, reduced the subjectivity of the triage decision, and was more accurate than other triage systems (Friedman et al., 2012).

One benefit of the ESI is the rapid identification of patients who need immediate attention. The focus of ESI triage is on quick sorting of patients in the setting of constrained resources. ESI triage is a rapid sorting into five groups with clinically meaningful differences in projected resource needs and, therefore, in associated operational needs. Use of the ESI for this rapid sorting can lead to improved flow of patients through the ED. For example, level 1 and 2 patients can be taken directly to the treatment area for rapid evaluation and treatment, while lower acuity patients can safely wait to be seen.

Other benefits of the ESI include determination of which patients do not need to be seen in the main ED and those who could safely and more efficiently be seen in a fast-track or urgent care area. For example, in many hospitals, the triage policy stipulates that all ESI level-4 and level-5 patients can be sent to either the medical fast track or minor trauma areas of the ED. The triage policy may also allow some level-3 patients to be sent to urgent care, such as patients needing simple migraine headache treatment. ESI level-3 patients triaged to urgent care and all patients sent to the acute area from urgent care for more serious conditions are monitored in the quality improvement program. Nurses using the ESI have reported the tool facilitates communication of patient acuity more effectively than the former three-level triage scales used at the sites (Wuerz et al., 2001). For example, the triage nurse can tell the charge nurse, "I need a bed for a level-2 patient," and through this common language, the charge nurse understands what is needed without a detailed explanation of the patient's condition by the triage nurse. Hospital administrators can use the case mix in real time to help make decisions regarding the need for additional resources or possibly diverting ambulance arrivals. If a waiting room has multiple level-2 patients with long waits, the hospital may need to develop a plan for the disposition of those patients who are waiting for an inpatient bed and occupying space in the ED.

The ESI also has been used as the foundation for ED policies that address specific populations. For example, the psychiatric service at one site is expected to provide consults for level-2 and level-3 patients with psychiatric complaints within 30 minutes of notification and for level-4 and level-5 patients within 1 hour. At another site, the ESI has been incorporated into a policy for patients greater than 20 weeks pregnant who present to the ED. Patients rated at ESI levels 1 and 2 are treated in the ED by emergency medicine with an obstetrical consult. Those rated 3, 4, or 5 are triaged to the labor and delivery area of the hospital.

Standardization of ED triage acuity data using the ESI is beneficial for secondary uses of ED data. For example, ED crowding researchers have incorporated the ESI into metrics for measuring and predicting ED crowding (Bernstein et al., 2003). Wider adoption of the ESI by U.S. hospitals could lead to the establishment of a true standard for triage acuity assessment, which will facilitate benchmarking, public health surveillance, and research.

References

American College of Emergency Physicians. (2003, 2010, 2017). ACEP policy statements: Triage scale standardization [Policy statement]. American College of Emergency Physicians. https://www.acep.

org/globalassets/new-pdfs/policy-statements/triage.scale.standardization.pdf

Barthell, E. N., Coonan, K., Finnell, J., Pollock, D., & Cochrane, D. (2004). Disparate systems, disparate data: integration, interfaces and standards in emergency medicine information technology. *Academic Emergency Medicine, 11*(11), 1142–1148. https://doi.org/10.1197/j.aem.2004.08.008

Baumann, M. R., & Strout, T. D. (2007). Triage of geriatric patients in the emergency department: Validity and survival with the Emergency Severity Index. *Annals of Emergency Medicine, 49*(2), 234–240. https://doi.org/10.1016/j.annemergmed.2006.04.011

Bernstein, S. L., Verghese, V., Leung, W., Lunney, A. T., & Perez, I. (2003). Development and validation of a new index to measure emergency department crowding. *Academic Emergency Medicine, 10*(9), 938–942. https://doi.org/10.1111/j.1553-2712.2003.tb00647.x

Centers for Disease Control and Prevention. (2008). *National Hospital Ambulatory Medical Care Survey: 2008 emergency department summary tables*. http://www.cdc.gov/nchs/data/ahcd/nhamcs_emergency/2008_ed_web_tables.pdf

Chi, C. J., & Huang, C. M. (2006). Comparison of the Emergency Severity Index (ESI) and the Taiwan Triage System in predicting resource utilization. *Journal of the Formosan Medical Association*, *105*(8), 617–625. https://doi.org/10.1016/S0929-6646(09)60160-1

Durani, Y., Brecher, D., Walmsley, D., Attia, M. W., & Loiselle, J. M. L. (2009). The Emergency Severity Index version 4: Reliability in pediatric patients. *Pediatric Emergency Care, 25*(11), 751–753. https://doi.org/10.1097/PEC.0b013e3181b0a0c6

Eitel, D. R., Travers, D. A., Rosenau, A., Gilboy, N., & Wuerz, R. C. (2003). The Emergency Severity Index version 2 is reliable and valid. *Academic Emergency Medicine, 10*(10), 1079–1080. https://doi.org/10.1197/S1069-6563(03)00350-6

Elshove-Bolk, J., Mencl, F., van Rijswijck, B. T. F, Simons, M. P., van Vugt, A. B. (2007). Validation of the Emergency Severity Index (ESI) in self-referred patients in a European emergency department. *Emergency Medicine, 24*, 170–174. https://doi.org/10.1136/emj.2006.039883

Emergency Nurses Association. (2017). Triage scale standardization [Joint policy statement with ACEP]. https://www.ena.org/docs/default-source/resource-library/practice-resources/position-statements/supported-statements/triage-scale-standardization.pdf?sfvrsn=a940caa_4

Fernandes, C., Tanabe, P., Gilboy, N., Johnson, L., McNair, R., Rosenau, A., Sawchuk, P., Thompson, D. A., Travers, D. A., Bonalumi, N., & Suter, R. E. (2005). Five level triage: A report from the ACEP/ENA Five Level Triage Task Force. *Journal of Emergency Nursing*, *31*(1), 39–50. https://doi.org/10.1066./j.jen.2004.11.002

Friedman Singer, R., Infante, A. A., Oppenheimer, C. C., West, C. A., & Siegel, B. (2012). The use of and satisfaction with the Emergency Severity Index. *Journal of Emergency Nursing*, *38*(2), 120–126. https://doi.org/10.1016/j.jen.2010.07.004

Gilboy, N., Travers, D. A., & Wuerz, R. C. (1999). Re-evaluating triage in the new millennium: A comprehensive look at the need for standardization and quality. *Journal of Emergency Nursing, 25*(6), 468 –473. https://doi.org/10.1016/s0099-1767(99)70007-3

Grossman, F. F., Nickel, C. H,. Christ, M., Schneider, K., Spirig, R., & Bingisser, R. (2011). Transporting clinical tools to new settings: Cultural adaptation and validation of the Emergency Severity Index in German. *Annals of Emergency Medicine, 57*(3), 257–264. https://doi.org/10.1016/j.annemergmed.2010.07.021

Institute of Medicine of the National Academies. (2006). *The Future of Emergency Care in the United States Health System*. *Annals of Emergency Medicine*, *48*(2), 115–120. https://doi.org/10.1016/j.annemergmed.2006.06.015

Haas, S. W., Travers, D., Tintinalli, J. E., Pollock, D., Waller, A., Barthell, E., Burt, C., Chapman, W., Coonan, K., Kamens, D., & McClay, J. (2008). Towards vocabulary control for chief complaint. *Academic Emergency Medicine*, *15*(5), 476–482. https://doi.org/10.1111/j.1553-2712.2008.00104.x

Handler, J. A., Adams, J. G., Feied, C. F., Gillam, M., Vozenilekv J., Barthell, E., & Davidson, S. J. (2004). Emergency medicine information technology consensus conference: Executive summary. *Academic Emergency Medicine*, *11*(11), 1112–1113. https://doi.org/10.1197/j.aem.2004.08.005

McHugh, M., Tanabe, P., Khar, R., & McClelland, M. (2011, June 1–5). The Emergency Severity Index is the most commonly used triage system in the U.S. [Paper presentation]. Society of Academic Emergency Medicine 2011 Annual Meeting, Boston, MA, United States.

National Center for Injury Prevention and Control. (1997). Data elements for emergency department systems. (Release 1.0). Atlanta, GA: Centers for Disease Control and Prevention. https://stacks.cdc.gov/view/cdc/6575

Niska, R., Bhuiya, F., & Xu, J. (2010). *National Hospital Ambulatory Medical Care Survey:* 2007 Emergency Department Summary. (National Health Statistics Reports No. 26). National Center for Health Statistics. https://www.cdc.gov/nchs/data/nhsr/nhsr026.pdf

Pedhazur, E, J., & Schmelkin, L. P. (1991). *Measurement, design and analysis: An integrated approach.* Erlbaum.

Platts-Mills, T. F., Travers, D., Biese, K., McCall, B., Kizer, S., LaMantia, M., Busby-Whitehead, J., & Cairns, C. B. (2010). Accuracy of the Emergency Severity Triage instrument for identifying elder emergency department patients receiving an immediate life-saving intervention. *Academic Emergency Medicine*, *17*(3), 238–243. https://doi.org/10.1111/j.1553-2712.2010.00670.x

Storm-Versloot, M. N., Ubbink, D. T., Chin a Choi, V., & Luitse, J. S. K. (2009). Observer agreement of the Manchester Triage System and the Emergency Severity Index: a simulation study. *Emergency Medicine Journal*, *26*, 556–560. https://doi.org/10.1136/emj.2008.059378

Tanabe, P., Gimbel, R., Yarnold, P. R., Kyriacou, D. N., & Adams, J. G. (2004). Reliability and validity of scores on the emergency

severity index version 3. *Academic Emergency Medicine*, *11*(1), 59–65. https://doi.org/10.1197/j.aem.2003.06.013

Tanabe, P., Travers, D., Gilboy. N., Rosenau, A., Sierzega, G., Rupp, V., Martinovich, Z., Adams, J. G. (2005). Refining Emergency Severity Index triage criteria. *Academic Emergency Medicine*, *12*(6), 497–501. https://doi.org/10.1197/j.aem.2004.12.015

Travers, D. A., Waller, A. E., Bowling, J. M, Flowers, D., Tintinalli, J. (2002). Five-level triage system more effective than three-level in tertiary emergency department. *Journal of Emergency Nursing*, *28*(5), 395 –400. https://doi.org/10.1067/men.2002.127184

Travers, D. A., Waller, A. E., Katznelson, J., & Agan, R. (2009). Reliability and validity of the Emergency Severity Index for pediatric triage. *Academic Emergency Medicine*, *16*(9), 843–849. https://doi.org/10.1111/j.1553-2712.2009.00494.x

Van der Wulp, I., Schrijvers, A. J. P., van Stel, H. F. (2009). Predicting admission and mortality with the Emergency Severity Index and the Manchester Triage System: A retrospective observational study. *Emergency Medicine Journal*, *26*(7), 506–509. https://doi.org/10.1136/emj.2008.063768

Waltz, C. F., Strickland, O. L., & Lenz, E. R. (1991). *Measurement in nursing research* (2nd ed.). F. A. Davis.

Welch, S., & Davidson, S. (2010). Exploring new intake models for the emergency department. *American Journal of Medical Quality*, *25(*3), 172–180. https://doi.org/10.1177/1062860609360570

Worster, A., Gilboy, N., Fernandes, C. M. , Eitel, D., Eva, K., Geisler, R., & Tanabe, P. (2004). Assessment of inter-observer reliability of two five-level triage and acuity scales: A randomized controlled trial. *Canadian Journal of Emergency Medicine*, *6*(4), 240–245. https://doi.org/10.1017/s1481803500009192

Wuerz, R. (2001). Emergency Severity Index triage category is associated with six-month survival.. *Academic Emergency Medicine*, *8*(1), 61–64. https://doi.org/10.1111/j.1553-2712.2001.tb00554.x

Wuerz, R., Milne, L. W., Eitel, D. R., Travers, D., & Gilboy, N. (2000). Reliability and validity of a new five-level triage instrument. *Academic Emergency Medicine*, *7*(3), 236–242. https://doi.org/10.1111/j.1553-2712.2000.tb01066.x

Wuerz, R., Travers, D., Gilboy, N., Eitel, D. R., Rosenau, A., & Yazhari, R. (2001). Implementation and refinement of the Emergency Severity Index. *Academic Emergency Medicine*, *8*(2), 170–176. https://doi.org/10.1111/j.1553-2712.2001.tb01283.x

2

Overview of the Emergency Severity Index

The Emergency Severity Index (ESI) is a simple to use, five-level triage algorithm that categorizes emergency department patients by evaluating both patient acuity and resource needs. Initially, the triage nurse assesses only the acuity level. If a patient does not meet high acuity level criteria (ESI level 1 or 2), the triage nurse then evaluates expected resource needs to help determine a triage level (ESI level 3, 4, or 5). The ESI is intended for use by nurses with triage experience or those who have attended a separate, comprehensive triage educational program.

Inclusion of resource needs in the triage rating is a unique feature of the ESI triage system. Acuity is determined by the stability of vital functions and the potential threat to life, limb, or organ. The triage nurse estimates resource needs based on previous experience with patients presenting with similar injuries or complaints. Resource needs are defined as the number of resources a patient is expected to consume in order for a disposition decision (discharge, admission, or transfer) to be reached. Once oriented to the algorithm, the triage nurse will be able to triage patients rapidly and accurately into one of five explicitly defined and mutually exclusive levels.

This chapter presents a step-by-step description and overview of how to triage using the ESI algorithm. Subsequent chapters explain key concepts in more detail and provide numerous examples to clarify the finer points of ESI.

Algorithms are frequently used in emergency care. Most emergency clinicians are familiar with the algorithms used in courses such as Basic Life Support, Advanced Cardiac Life Support, and the Trauma Nursing Core Course. These courses present a step-by-step approach to clinical decision making that the clinician is able to internalize with practice. The ESI algorithm follows the same principles.

Each step of the algorithm guides the user toward the appropriate questions to ask or the type of information to gather. Based on the data or answers obtained, a decision is made, and the user is directed to the next step and ultimately to the determination of a triage level.

Figure 2-1. Emergency Severity Index Conceptual Algorithm, v4

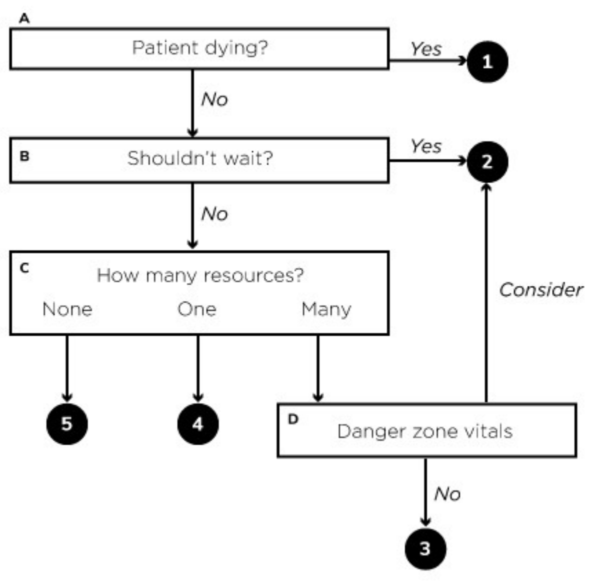

A conceptual overview of the ESI algorithm is presented in Figure 2-1 to illustrate the major ESI decision points. The ESI algorithm itself is shown in Figure 2-2. The algorithm uses four decision points (A, B, C, and D) to sort patients into one of the five triage levels. Triage with the ESI algorithm requires an experienced ED nurse, who starts at the top of the algorithm. With practice, the triage nurse will be able to rapidly move from one ESI decision point to the next.

The four decision points depicted in the ESI algorithm are critical to accurate and reliable application of ESI. The figure shows the four decision points reduced to four key questions:

A. Does this patient require immediate lifesaving intervention?
B. Is this a patient who should not wait?
C. How many resources will this patient need?
D. What are the patient's vital signs?

Figure 2-2. ESI Triage Algorithm, v4

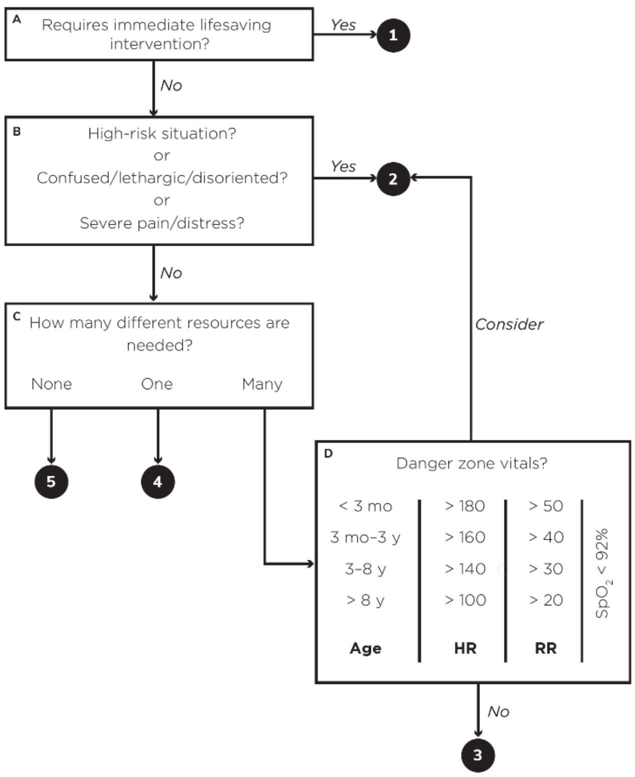

A. **Immediate lifesaving intervention required**: Airway, emergency medications, or other hemodynamic interventions (intravenous access, supplemental oxygen, monitor, electrocardiogram and labs DO NOT count); and any of the following clinical conditions: intubated, apneic, pulseless, severe respiratory distress, pulse oximetry (SpO_2) < 90%, acute mental status changes, or unresponsive.

Unresponsiveness is defined as a patient who is either

1. Nonverbal and not following commands (acutely) or
2. Requires noxious stimulus (P or U on AVPU scale).

B. **High risk situation:** A patient you would put in your last open bed.

Severe pain/distress is determined by clinical observation and/or patient rating of greater than or equal to 7 on 0-10 pain scale.

C. **Resources**: Count the number of different types of resources, not the individual tests or radiographs (e.g.,: complete blood count, electrolytes, and coagulants equals one resource, while completed blood count plus chest radiograph equals two resources).

Resources	Not Resources
• Labs (blood, urine)<br>• Electrocardiogram, radiographs<br>• Computed tomography, magnetic resonance imaging, ultrasound, angiography	• History and physical exam (including pelvic)<br>• Point-of-care testing
• Intravenous fluids (hydration)	• Saline or heparin lock
• Intravenous, intramuscular, or nebulized medications	• Oral medications<br>• Tetanus immunization<br>• Prescription refills
• Specialty consultation	• Phone call to primary care physician
• Simple procedure = 1 (laceration repair, urinary catheter)<br>• Complex procedure = 2 (procedural sedation)	• Simple wound care (dressings, recheck))<br>• Crutches, splints, slings

D. **Danger Zone Vital Signs**

Consider uptriage to ESI level 2 if any vital sign criterion is exceeded.

Pediatric Fever Considerations

1–28 days of age: assign at least ESI level 2 if T > 38° C (100.4°F)

1–3 months of age: consider assigning ESI level 2 if T > 38°C (100.4°F)

3 months–3 years of age: Consider assigning ESI 3 if:

1. T > 39°C (102.2°F), or
2. Incomplete immunizations, or
3. No obvious source of fever

The answers to these questions guide the user to the correct triage level.

The following sections examine the answers to these questions by detailing each of the decision points, A–D.

Decision Point A: Does the Patient Require Immediate Lifesaving Intervention?

Simply stated, at decision point A (Figure 2-3) the triage nurse asks, "Does this patient require immediate lifesaving intervention?" If the answer is "yes," the triage process is complete, and the patient is automatically triaged as ESI level 1. A "no" answer moves the user to the next step in the algorithm, decision point B.

Figure 2-3. Decision Point A: Is the Patient Dying?

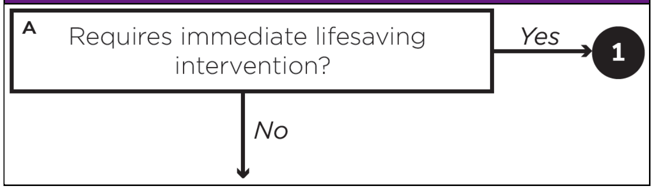

The following questions are used to determine whether the patient requires an immediate lifesaving intervention:

- Does this patient have a patent airway?
- Is the patient breathing?
- Does the patient have a pulse?
- Is the nurse concerned about the pulse rate, rhythm, and quality?
- Is the nurse concerned about this patient's ability to deliver adequate oxygen to the tissues?
- Was this patient intubated pre-hospital because of concerns about the patient's ability to maintain a patent airway, spontaneously breathe, or maintain oxygen saturation?
- Does the patient require an immediate medication, or other hemodynamic intervention such as volume replacement or blood?
- Does the patient meet any of the following criteria: already intubated, apneic, pulseless, severe respiratory distress, $SpO_2 < 90$ percent, acute mental status changes, or unresponsive?

Research has demonstrated that the triage nurse is able to accurately predict the need for immediate lifesaving interventions (Tanabe, et al., 2005). Table 2-1 lists interventions that are considered lifesaving and those that are not, for the purposes of ESI triage.

Interventions not considered lifesaving include some that are diagnostic or therapeutic but none that would save a life. Lifesaving interventions are aimed at securing an airway, maintaining breathing, supporting circulation, or addressing a major change in level of consciousness (LOC).

The ESI level-1 patient always presents to the emergency department with an unstable condition. Because the patient could die without immediate care, a team response is initiated: the physician is at the bedside, and nursing is providing critical care. ESI level-1 patients are seen immediately because timeliness of interventions can affect morbidity and mortality.

Immediate physician involvement in the care of the patient is a key difference between ESI level-1 and ESI level-2 patients. Level-1 patients are critically ill and require immediate physician evaluation and interventions. When considering the need for immediate lifesaving interventions, the triage nurse carefully evaluates the patient's respiratory status and oxygen saturation. A patient in severe respiratory distress or with an $SpO_2 < 90$ percent may still be breathing but needs immediate intervention to maintain an airway and oxygenation status. This is the patient who will require the physician in the room ordering medications such as those used for rapid sequence intubation or preparing for other interventions for airway and breathing.

Each patient with chest pain must be evaluated within the context of the level-1 criterion to determine whether the patient requires an immediate lifesaving intervention. Some patients presenting with chest pain are very stable. Although they may require a diagnostic electrocardiogram within 10 minutes of arrival, these patients do not meet the level-1 criterion.

However, patients who are pale, diaphoretic, in acute respiratory distress, or hemodynamically unstable do meet the level-1 criterion and will require immediate lifesaving interventions.

When determining whether the patient requires immediate lifesaving intervention, the triage nurse must also assess the patient's level of responsiveness. The ESI algorithm uses the AVPU (alert, verbal, pain, unresponsive) scale (Table 2-2). The goal for this part of the algorithm is to identify the patient who has a recent and/or sudden change in level of conscience and requires immediate intervention. The triage nurse needs to identify patients who are non-verbal or require noxious stimuli to obtain a response. ESI uses the AVPU scale and patients that score a P (pain) or U (unresponsive) on the AVPU scale meet the level-1 criterion. Unresponsiveness is assessed in the context of acute changes in neurological status, not for the patient who has known developmental delays, documented dementia, or aphasia. Any patient who is unresponsive, including the intoxicated patient who is unresponsive to painful stimuli, meets the level-1 criterion and should receive immediate

Table 2-1. Immediate Lifesaving Interventions

	Lifesaving	Not Lifesaving
Airway/breathing	• Assisted ventilation<br>• Intubation<br>• Surgical airway<br>• Emergent non-invasive positive pressure ventilation	Oxygen administration<br>• Nasal cannula<br>• Non-rebreather
Electrical Therapy	• Defibrillation<br>• Emergent cardioversion<br>• External pacing	• Cardiac monitor
Procedures	• Chest needle decompression<br>• Pericardiocentesis<br>• Open thoracotomy<br>• Intraosseous access	Diagnostic Tests<br>• Electrocardiogram<br>• Labs<br>• Ultrasound<br>• Focused assessment with sonography for trauma (FAST)
Hemodynamics	• Significant intravenous fluid resuscitation<br>• Blood administration<br>• Control of major bleeding	• Intravenous access<br>• Saline lock for medications
Medications	• Naloxone<br>• Dextrose<br>• Dopamine<br>• Atropine<br>• Adenosine	• Aspirin<br>• Intravenous nitroglycerin<br>• Antibiotics<br>• Heparin<br>• Pain medications<br>• Respiratory treatments with beta agonists

Table 2-2. Four Levels of the AVPU Scale

AVPU Level	Level of Consciousness
A	Alert. The patient is alert, awake and responds to voice. The patient is oriented to time, place, and person. The triage nurse is able to obtain subjective information.
V	Verbal. The patient responds to verbal stimuli by opening their eyes when someone speaks to them. The patient is not fully oriented to time, place, or person.
P	Painful. The patient does not respond to voice, but does respond to a painful stimulus, such as a squeeze to the hand or sternal rub. A noxious stimulus is needed to elicit a response.
U	Unresponsive. The patient is nonverbal and does not respond even when a painful stimulus is applied

Blansfield, J. (Ed.). (2019). *Trauma Nursing Core Course provider manual* (8th ed., p. 30). Emergency Nurses Association.

evaluation. An example of a recent mental status change that would require immediate intervention would be a patient with decreased mental status who is unable to maintain a patent airway or is in severe respiratory distress.

An ESI level-1 patient is not always brought to the emergency department by ambulance. The patient or his or her family member may not realize the severity of the illness and, instead of calling an ambulance, may drive the patient to the emergency department. The patient with a drug overdose or acute alcohol intoxication may be dropped at the front door. Infants and children, because they are "portable," may be brought to the ED by car and carried into the emergency department. The experienced triage nurse is able to instantly identify this critical patient. With a brief, "across-the-room" assessment, the triage nurse recognizes the patient that is in extremis. Once identified, this patient is taken immediately to the treatment area and resuscitation efforts are initiated.

Patients assessed as an ESI level 1 constitute approximately 1 percent to 3 percent of all ED patients (Eitel, et al., 2003; Wuerz, Milne, Eitel, Travers, & Gilboy, 2000; Wuerz, et al., 2001). Upon arrival, the patient's condition requires immediate lifesaving interventions from either the emergency physician and nurse or the trauma or code team. From ESI research we know that most ESI level-1 patients are admitted to intensive care units, while some die in the emergency department (Eitel, et al., 2003; Wuerz, 2001). A few ESI level-1 patients are discharged from the ED if they have a reversible change in level

of consciousness or vital functions such as with hypoglycemia, seizures, alcohol intoxication, or anaphylaxis.

Examples of ESI level 1 include the following:

- Cardiac arrest
- Respiratory arrest
- Severe respiratory distress
- $SpO_2 < 90$
- Critically injured trauma patient who presents unresponsive
- Overdose with a respiratory rate of 6 breaths/minute
- Severe respiratory distress with agonal or gasping type respirations
- Severe bradycardia or tachycardia with signs of hypoperfusion
- Hypotension with signs of hypoperfusion
- Trauma patient who requires immediate crystalloid and colloid resuscitation
- Chest pain, pale, diaphoretic, systolic blood pressure 70 by palpation
- Weak and dizzy, heart rate = 30 beats/minute
- Anaphylactic shock
- Baby that is flaccid
- Unresponsive patient with a strong odor of alcohol
- Hypoglycemia with a change in mental status
- Intubated head bleed with unequal pupils
- Child who fell out of a tree and is unresponsive to painful stimuli

Figure 2-4. Decision Point B: Should the Patient Wait?

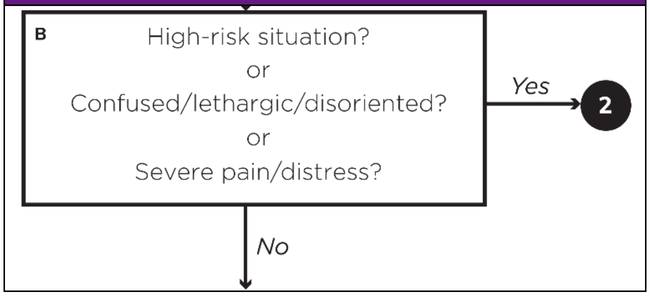

Decision Point B: Should the Patient Wait?

Once the triage nurse has determined the patient does not meet the criterion for ESI level 1, the triage nurse moves to decision point B (Figure 2-4) At decision point B, the nurse needs to decide whether this patient is a patient who should not wait to be seen. If the patient should not wait, the patient is triaged as ESI level 2. If the patient can wait, then the user moves to the next step in the algorithm.

Three broad questions are used to determine whether the patient meets level-2 criteria:

- Is this a high-risk situation?
- Is the patient confused, lethargic, or disoriented?
- Is the patient in severe pain or distress?

The triage nurse obtains pertinent subjective and objective information to quickly answer these questions. A brief introduction to ESI level-2 criteria is presented here, while a more detailed explanation of which patients meet ESI level-2 criteria is presented in Chapter 3, "ESI Level 2."

Is This a High-Risk Situation?

Based on a brief patient interview, gross observations, and finally the "sixth sense" that comes from experience, the triage nurse identifies the patient who is high risk. Frequently the patient's age and past medical history influence the triage nurse's determination of risk.

A high-risk patient is one whose condition could easily deteriorate or who presents with symptoms suggestive of a condition requiring time-sensitive treatment. This is a patient who has a potential threat to life, limb, or organ.

A high-risk patient does not require a detailed physical assessment or even a full set of vital signs in most cases. The patient may describe a clinical portrait that the experienced triage nurse recognizes as a high-risk situation. An example is the patient who states, "I never get headaches, and I lifted this heavy piece of furniture, and now I have the worst headache of my life." The triage nurse would triage this patient as ESI level 2 because the symptoms suggest the possibility of a subarachnoid hemorrhage.

When the patient is categorized as ESI level 2, the triage nurse has determined that it would be unsafe for the patient to remain in the waiting room for any length of time. While ESI does not suggest specific time intervals, ESI level-2 patients remain a high priority, and generally, placement and treatment should be initiated rapidly. ESI level-2 patients are very ill and at high risk. The need for care is immediate and an appropriate bed needs to be found. Usually, rather than move to the next patient, the triage nurse determines that the charge nurse or staff in the patient care area should be immediately alerted that they have an ESI level 2 patient. Unlike with level-1 patients, the emergency nurse can initiate care through protocols without a physician immediately at the bedside. The nurse recognizes that the patient needs interventions but is confident that the patient's clinical condition will not deteriorate. The nurse can initiate intravenous access, administer supplemental oxygen, obtain an electrocardiogram, and place the patient on a cardiac monitor, all before a physician is needed. Although the physician does not need to be present immediately, he or she should be notified that the patient is there and has been classified as an ESI level-2 patient.

Examples of high-risk situations include the following:

- Active chest pain, suspicious for acute coronary syndrome but does not require an immediate lifesaving intervention, stable
- A needle stick in a health care worker
- Signs of a stroke, but does not meet level-1 criteria
- A possible ectopic pregnancy, hemodynamically stable
- A patient on chemotherapy and therefore immunocompromised, with a fever
- A suicidal or homicidal patient

Chapter 3 contains additional information on high-risk situations.

Is the Patient Confused, Lethargic, or Disoriented?

This is the second question to be asked at decision point B. Again, the concern is whether the patient is demonstrating an acute change in level of consciousness. Patients with a baseline mental status of confusion do not meet level-2 criteria.

Examples of patients who are confused, lethargic, or disoriented include the following:

- New onset of confusion in an elderly patient
- The 3-month-old whose mother reports the child is sleeping all the time
- The adolescent found confused and disoriented

Each of these examples indicates that the brain may be either structurally or chemically compromised.

Is the Patient in Severe Pain or Distress?

The third question the triage nurse needs to answer at decision point B is whether this patient is currently in pain or distress. If the answer is "no," the triage nurse is able to move to the next step in the algorithm. If the answer is "yes," the triage nurse needs to assess the level of pain or distress. This is determined by clinical observation and/or a self-reported pain rating of 7 or higher on a scale of 0 to 10. When patients report pain ratings of 7/10 or greater, the triage nurse **may** triage the patient as ESI level 2 but is **not required** to assign a level-2 rating.

Pain is one of the most common reasons for an ED visit, and clearly all patients reporting pain 7/10 or greater **do not** need to be assigned an ESI level-2 triage rating. A patient with a sprained ankle presents to the ED and rates their pain as 8/10. This patient's pain can be addressed with simple nursing interventions: wheelchair, elevation, and application of ice. This patient is safe to wait and should not be assigned to ESI level 2 based on pain.

In some patients, pain can be assessed by clinical observation:

- Distressed facial expression, grimacing, crying
- Diaphoresis
- Body posture
- Changes in vital signs – hypertension, tachycardia, and increased respiratory rate

The triage nurse observes physical responses to acute pain that support the patient's rating. For example, the patient with abdominal pain who is diaphoretic, tachycardic, and has an elevated blood pressure or the patient with severe flank pain, vomiting, pale skin, and a history of renal colic are both good examples of patients that meet ESI level-2 criteria.

The triage nurse should also consider the question, "Would I give my last open bed to this patient?" If the answer is yes, then the patient meets the criteria for ESI level 2.

Chapter 3 provides additional information on ESI level 2 and pain.

Severe distress can be physiological or psychological. Examples of distress include the sexual assault victim, the victim of domestic violence, the combative patient, or the bipolar patient who is currently manic.

ESI level-2 patients constitute approximately 20 to 30 percent of emergency department patients (Travers, et al., 2002; Wuerz, et al., 2001; Tanabe, Gimbel, et al., 2004). Once an ESI level-2 patient is identified, the triage nurse needs to ensure that the patient is cared for in a timely manner. Registration can be completed by a family member or at the bedside. ESI level-2 patients need vital signs and a comprehensive nursing assessment but not necessarily at triage. Placement in the treatment area is a priority and should not be delayed to finish obtaining vital signs or ask additional questions. ESI research has shown that 50 to 60 percent of ESI level-2 patients are admitted from the ED (Wuerz, et al., 2001).

Decision Point C: Resource Needs

If the answers to the questions at the first two decision points are "no," then the triage nurse moves to decision point C (Figure 2-5).

Figure 2-5. Resource Prediction

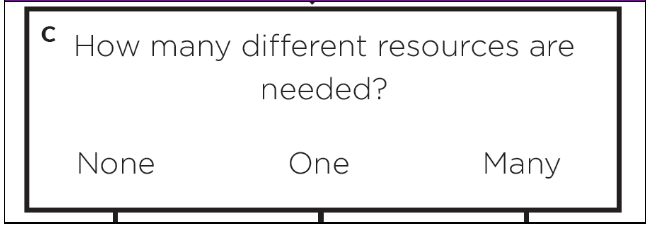

The triage nurse should ask, "How many different resources do you think this patient is going to consume in order for the physician to reach a disposition decision?" The disposition decision could be to send the patient home, admit to the observation unit, admit to the hospital, or even transfer to

Table 2-3. ESI Resources	
**Resources**	**Not Resources**
• Labs (blood, urine)<br>• Electrocardiogram, radiographs<br>• Computed tomography, magnetic resonance imaging, ultrasound, angiography	• History and physical exam (including pelvic)<br>• Point-of-care testing
• Intravenous fluids (hydration)	• Saline or heparin lock
• Intravenous, intramuscular, or nebulized medications	• Oral medications<br>• Tetanus immunization<br>• Prescription refills
• Specialty consultation	• Phone call to primary care physician
• Simple procedure = 1 (laceration repair, urinary catheter)<br>• Complex procedure = 2 (procedural sedation)	• Simple wound care (dressings, recheck))<br>• Crutches, splints, slings

another institution. This decision point again requires the triage nurse to draw from past experiences in caring for similar emergency department patients. ED nurses need to clearly understand that the estimate of resources has to do with standards of care and is independent of type of hospital (i.e., teaching or non-teaching) location of the hospital (urban or rural), or which provider is working that day. A patient presenting to any emergency department should consume the same general resources in one ED as in any other ED.

Considering the patient's brief subjective and objective assessment, past medical history, allergies, medications, age, and gender, how many different resources will be used in order for the physician to reach a disposition? In other words, what is typically done for the patient who presents to the emergency department with this common complaint? The triage nurse is asked to answer these questions based on his or her assessment of the patient and should not consider individual practice patterns but rather the routine practice in the particular ED.

To identify resource needs, the triage nurse must be familiar with emergency department standards of care. The nurse must be knowledgeable about the concept of "prudent and customary." One easy way to think about this concept is to ask the question, "Given this patient's chief complaint or injury, which resources are the emergency physician likely to utilize?" Resources can be hospital services, tests, procedures, consults or interventions that are above and beyond the physician history and physical or very simple ED interventions such as applying a bandage. Further explanations and examples are provided in Chapter 4, "ESI Levels 3–5 and Expected Resource Needs."

A list of what is and is not considered a resource for purposes of ESI triage classification can be found in Table 2-3. ESI level-3 patients are predicted to require two or more resources; ESI level-4 patients are predicted to require one resource; and ESI level-5 patients are predicted to require no resources (Table 2-4). Research has shown that ESI level-3 patients make up 30 to 40 percent of patients seen in the emergency department (Eitel et al., 2003; Wuerz et al., 2001). ESI level 3 patients present with a chief complaint that requires an in-depth evaluation. An example is patients with abdominal pain. They often require a more in-depth evaluation but are felt to be stable in the short term and certainly may have a longer length of stay in the ED. ESI level 4 and ESI level 5 make up between 20 and 35 percent of ED volume, perhaps even more in a community with poor primary care access. Appropriately trained mid-level providers with the right skills mix could care for these patients in a fast-track or express care setting, recognizing that a high proportion of these patients have a trauma-related presenting complaint.

Decision Point D: The Patient's Vital Signs

Before assigning a patient to ESI level 3, the nurse needs to look at the patient's vital signs and decide whether they are outside the accepted parameters for age and are felt by the nurse to be meaningful. If the vital signs are outside accepted parameters, the triage nurse should consider upgrading the triage level to ESI level 2. However, it is the triage nurse's decision as to whether or not the patient should be upgraded to an ESI level 2 based on vital sign abnormalities. This is decision point D.

Vital sign parameters are outlined by age in Figure 2-6. The vital signs used are heart rate, respiratory rate, and oxygen saturation by pulse oximetry and, for any child under age 3, body temperature. Using the vital sign criteria, the triage nurse can upgrade an adult patient who presents with a heart rate of 104, or this patient can remain ESI level 3. A 6-month-old baby with a cold and a respiratory rate of 48 could be triaged as ESI level 2 or 3. Based on the patient's history and physical assessment, the nurse must ask if the vital signs are enough of a concern to say that the patient is high risk and cannot wait to be seen. Chapter 5, "The Role of Vital Signs in ESI Triage," explains vital signs in detail and provides examples.

Temperature is only included for children under age 3. Significant fever may exclude young children from categories 4 and 5. This will help identify potentially bacteremic children and avoid sending them to a fast track setting or keeping them waiting a prolonged time. Pediatric fever guidelines are described in detail in Chapter 5.

Table 2-4. Predicting Resources

ESI Level	Patient Presentation	Interventions	Resources
5	Healthy 10-year-old child with poison ivy	Needs an exam and prescription	None
5	Healthy 52-year-old male ran out of blood pressure medication yesterday, BP 150/92 mm Hg	Needs an exam and prescription	None
4	Healthy 19-year-old with sore throat and fever	Needs an exam, throat culture, prescriptions	Lab (throat culture) *
4	Healthy 29-year-old female with a urinary tract infection, denies vaginal discharge	Needs an exam, urine, urine culture, maybe urine pregnancy, and prescriptions	Lab (urine, urine culture and sensitivity, urine pregnancy) **
3	A 22-year-old male with right lower quadrant abdominal pain since early this morning, plus nausea, no appetite	Needs an exam, lab studies, Intravenous fluid, abdominal computed tomography scan, and perhaps surgical consult	Two or more
3	A 45-year-old obese female with left lower leg pain and swelling, started 2 days ago after driving in a car for 12 hours	Needs exam, lab, lower extremity non-invasive vascular studies	Two or more

* In some regions throat cultures are not routinely performed; instead, the patient is treated based on history and physical exam. If that is the case the patient would be an ESI level 5.
** All 3 tests count as one resource (Lab).

Figure 2-6. Danger Zone Vital Signs

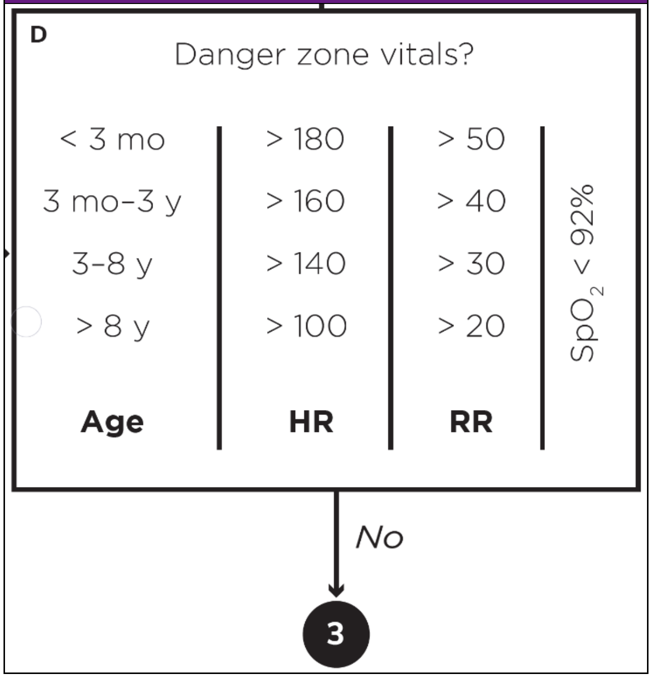

Does Time to Treatment Influence ESI Triage Categories?

An estimate of how long the patient can wait to be seen by a physician is an important component of most triage systems. The Australasian and Canadian Triage Systems both require patients to be seen by a physician within a specific time period, based on their triage category. ESI does not mandate specific time standards in which patients must be evaluated by a physician. However, patients who meet criteria for ESI level 2 should be seen as soon as possible; it is up to the individual institution to determine specific policies for what constitutes "as soon as possible."

Frequently, there may be confusion between institutional policy and "flow or process of patient care" and ESI triage level. Examples of patient scenarios in which flow and triage category may seem to conflict follow.

Often trauma patients present to the triage nurse after sustaining a significant mechanism of injury, such as an unrestrained passenger in a high-speed motor vehicle crash. The patient may have left the crash scene in some way other than by ambulance and presents to triage with localized right upper quadrant pain with stable vital signs. The patient is physiologically stable, walked into the ED, and does not meet ESI level-1criteria. However, the patient is at high risk for a liver laceration and other significant trauma so should be triaged as ESI level 2.

Frequently, EDs have trauma policies and trauma response level categorization that will require rapid initiation of care. Triage and trauma response level are both important and should be recorded as two different scores. While the triage nurse recognizes this is a physiologically stable trauma patient and correctly assigns ESI level 2, he or she should facilitate patient placement and trauma care as outlined by the trauma policy. The patient is probably stable for another 10 minutes and does not require immediate lifesaving interventions. If the same

patient presented with a systolic blood pressure of 80 by palpation, the patient would be triaged as ESI level 1 and require immediate hemodynamic, lifesaving interventions.

Another example of policies that may affect triage level is triage of the patient with stable chest pain. If the patient is physiologically stable but experiencing chest pain, it could potentially be an acute coronary syndrome. The patient meets ESI level-2 criteria. He or she does not require immediate lifesaving interventions but is a high-risk patient. Care is time sensitive; an electrocardiogram should be performed within 10 minutes of patient arrival. Often, EDs will have a policy related to rapid initiation of an electrocardiogram. While care of these patients should be rapidly initiated, the electrocardiogram is not a lifesaving intervention It is a diagnostic procedure. If the triage nurse were to triage all chest pain patients as ESI level 1, it would be difficult to prioritize the care for true ESI level-1 patients who require immediate lifesaving interventions. But the patient with chest pain who presents to triage diaphoretic, with a systolic blood pressure of 80 by palpation would meet ESI level-1 criteria.

A third example of time-sensitive care is a patient who presents with signs of an acute stroke. For example, the patient who reports left arm weakness meets the criteria for ESI level 2, and the stroke team needs to be activated immediately. Time to computed tomography completion is a quality measure that must be met. But the patient with signs of stroke who is unable to maintain an airway meets ESI level-1 criteria. The stroke team would also be activated.

Finally, a somewhat different scenario is an elderly patient who fell, may have a fractured hip, arrives by private car with family, and is in pain. The patient does not really meet ESI level-2 criteria but is very uncomfortable. The triage nurse would categorize the patient as ESI level 3 and probably place the patient in an available bed before other ESI level-3 patients. Ambulance patients may also present with a similar scenario. Arriving by ambulance is not a criterion to assign a patient ESI level 1 or 2. The ESI criteria should always be used to determine triage level without regard to method of arrival.

In general, care of ESI level-2 patients should be rapidly facilitated and the role of the charge nurse or flow manager is to know where these patients can be placed in the treatment area on arrival. All level-2 patients are still potentially very ill and require rapid initiation of care and evaluation. The triage nurse has determined that it is unsafe for these patients to wait. Patients currently may be stable but may have a condition that can easily deteriorate. Initiation of diagnostic treatment may be time sensitive (stable chest pain requires an electrocardiogram within 10 minutes of arrival) or the patient may have a potential major life or organ threat. ESI level-2 patients are still considered to be very high risk.

In the current atmosphere of ED crowding, it is not uncommon for the triage nurse to be in a situation of triaging many ESI level-2 patients with no open ED rooms in which to place the patients. In these situations, the triage nurse may be tempted to "under-triage." This can lead to serious, negative patient outcomes and an underrepresentation of the ED's overall case mix. When faced with multiple ESI level-2 patients simultaneously, the triage nurse must evaluate each patient according to the ESI algorithm. Then, the nurse can "triage" all level-2 patients to determine which patient(s) are at highest risk for deterioration, in order to facilitate patient placement based on this evaluation. For example, the patient with chest pain would be brought in before the patient with a kidney stone.

Summary

In summary, the ESI is a five-level triage system that is simple to use and divides patients by acuity and resource needs. The ESI triage algorithm is based on four key decision points. The experienced ED RN will be able to triage patients using this system rapidly and accurately.

> **NOTE** Appendix A, "Frequently Asked Questions and Post-Quiz Materials for Chapters 2–8," includes frequently asked questions and post-test assessment questions for Chapters 2 through 8. These sections can be incorporated into a locally developed ESI training course.

References

Blansfield, J. (Ed.). (2019). *Trauma Nursing Core Course provider manual* (8th ed.). Emergency Nurses Association.

Eitel, D. R., Travers, D. A., Rosenau, A., Gilboy, N., & Wuerz, R. C. (2003). The Emergency Severity Index version 2 is reliable and valid. *Academic Emergency Medicine, 10*(10), 1079–1080. https://doi.org/10.1197/S1069-6563(03)00350-6

Tanabe, P., Gimbel, R., Yarnold, P. R., Kyriacou, D. N., & Adams, J. G. (2004). Reliability and validity of scores on the emergency severity index version 3. *Academic Emergency Medicine*, *11*(1, 59–65. https://doi.org/10.1197/j.aem.2003.06.013

Tanabe, P., Travers, D., Gilboy. N., Rosenau, A., Sierzega, G., Rupp, V., Martinovich, Z., Adams, J. G. (2005). Refining Emergency Severity Index triage criteria. *Academic Emergency Medicine,*. *12*(6), 497–501. https://doi.org/10.1197/j.aem.2004.12.015

Travers, D. A., Waller, A. E., Bowling, J. M, Flowers, D., Tintinalli, J. (2002). Five-level triage system more effective than three-level in tertiary emergency department. *Journal of Emergency Nursing*, *28*(5), 395 –400. https://doi.org/10.1067/men.2002.127184

Wuerz, R. (2001). Emergency Severity Index triage category is associated with six-month survival. *Academic Emergency Medicine*, *8*(1), 61–64. https://doi.org/10.1111/j.1553-2712.2001.tb00554.x

Wuerz, R., Milne, L. W., Eitel, D. R., Travers, D., & Gilboy, N. (2000). Reliability and validity of a new five-level triage instrument. *Academic Emergency Medicine*, *7*(3), 236–242. https://doi.org/10.1111/j.1553-2712.2000.tb01066.x

Wuerz, R., Travers, D., Gilboy, N., Eitel, D. R., Rosenau, A., & Yazhari, R. (2001). Implementation and refinement of the Emergency Severity Index. *Academic Emergency Medicine*, *8*(2), 170–176. https://doi.org/10.1111/j.1553-2712.2001.tb01283.x

3

ESI Level 2

ESI level-2 criteria are the most frequently misinterpreted criteria. This chapter expands on the information contained in Chapter 2 and discusses in greater detail the decision-making process required to determine which patients meet ESI level-2 criteria. A complete understanding of level-2 criteria is critical to avoid both under- and overtriage of patients.

ED nurses are often reluctant to assign level 2 to patients who meet the criteria when the ED is crowded and there are long waits. It is important for nurses to understand that the triage nurse's primary responsibility is to assign the correct triage level. A patient who is undertriaged may wait for prolonged periods before being evaluated by a physician. This delay in care may result in negative patient outcomes. These cases are the type most frequently involved in litigation.

Triage nurses without sufficient ED experience may be at risk for overtriaging patients. While it is always safer to overtriage than to undertriage, overtriage presents its own set of problems. If a nurse triages most patients as ESI level 2, beds will not be available for true level-1 and level-2 patients when needed, and physician and nurse colleagues will begin to lose confidence in the nurse, his or her triage level judgements, and eventually, the validity of ESI. If the algorithm is not used independently of the number and type of patients surging into an ED, then the accurate application of data for off-line planning will be subverted. When a hospital is implementing the ESI in an ED, considerable time should be devoted to explaining which types of patients should be categorized ESI level 2. In this chapter we highlight common patient presentations that meet ESI level-2 criteria.

After the triage nurse has determined the patient does not require immediate lifesaving intervention, he or she must then decide whether the patient should wait. When making this decision, the triage nurse should consider the following questions: "Would I use my last open bed for this patient?" or "Would I make an alternative bed for this patient in the hallway due to the criticality and time sensitivity of appropriate intervention?" Patients who meet ESI level-1 criteria require immediate resuscitation. Patients who meet ESI level-2 criteria should have their placement rapidly facilitated. ESI does not specify timeframe to physician evaluation, unlike many other triage systems. However, it is understood that level-2 patients should be evaluated as soon as possible.

The following three questions, also listed in Figure 3-1, should be answered and are key components of ESI level-2 criteria:

- Is this a high-risk situation?
- Is the patient experiencing new onset confusion, lethargy, or disorientation?
- Is the patient experiencing severe pain or distress?

Figure 3-1. Patient Assessment

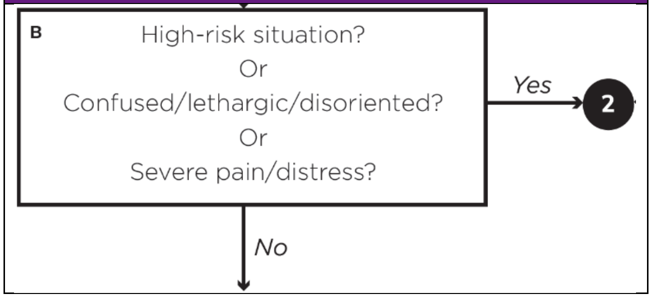

An experienced triage nurse will always assess the patient's chief complaint, presenting signs and symptoms, demographics, and medical history to attempt to identify a high-risk situation.

While the purpose of nurse triage is not to make a medical diagnosis, these situations are based on the experienced triage nurse's knowledge of possible medical diagnoses that are associated with specific chief complaints. A good source of information about the signs and symptoms of various medical diagnoses is the *Emergency Nursing Core Curriculum* (Sweet, 2018) or other emergency nursing textbooks. The following discussion provides some selected examples of high-risk situations. This discussion is not intended to be an exhaustive list. However, it should provide illustrative examples that help drive home the important concepts involved in making these broader but crucial clinical judgements.

1. Is This a High-Risk Situation?

The ability to recognize a high-risk situation is a critical element of the triage decision-making process, *regardless of the triage system used*. ESI highlights the importance of recognizing high-risk situations and uses the triage nurse's expertise and experience to identify patients at high risk.

Little has been written about how ED triage nurses make decisions. Knowledge and experience are necessary but not sufficient. Novice triage nurses are taught symptom clustering such as the cardiac cluster of chest pain with nausea, shortness of breath, and diaphoresis. From prior clinical situations, ED nurses put together "clinical portraits." The nurses store patient scenarios in which they were involved in some way. For example, the scenario of a patient with fever, stiff neck, and a meningococcal rash should trigger recognition of meningitis, a high-risk situation. The nurse should then have a high index of suspicion when a patient with a similar set of complaints presents to triage.

Vital signs are not always helpful in the identification of high-risk patients. More frequently, patients present to the ED with a chief complaint, signs and symptoms, or history suggestive of a problem or condition that is serious and, unless dealt with promptly, can deteriorate rapidly. These are considered high-risk situations and often interpretation of the patient's vital sign data is not required to make the decision that this patient scenario is high-risk. For example, a patient who states that he is allergic to peanuts and just came from a restaurant with throat tightening can be triaged as ESI level 2 (if he does not meet level-1 criteria), prior to obtaining vital signs. The patient is at high risk for anaphylaxis and requires rapid evaluation. Often, patient age, past medical history, and current medications influence the perceived severity of the chief complaint. For example, a frail elderly patient with severe abdominal pain is at a much higher risk of morbidity and mortality than a previously healthy 20-year-old. The elderly patient with abdominal pain should be classified as ESI level 2, while the 20-year-old with stable vital signs will usually be classified as ESI level 3.

It is common for the triage nurse to identify a high-risk situation that may be supported by abnormal vital signs. For example, a patient with a fever and productive cough may have a respiratory rate of 32 and an oxygen saturation of 90 percent. The experienced triage nurse uses knowledge and expertise to recognize that this patient probably has pneumonia and is at high risk for oxygen desaturation.

Inexperienced ED nurses are not likely to consistently identify high-risk situations and make accurate triage decisions because they have not incorporated symptom clustering and clinical portraits into their practices; such approaches are key in identifying the high-risk patient situation.

Abdominal and Gastrointestinal

Abdominal pain is the most frequent chief complaint evaluated in the ED. What distinguishes high-risk abdominal pain? A good history and assessment of current pain rating, respiratory rate, and heart rate, as well as patient demographics, are important elements to consider that will help determine the presence of a high-risk situation.

Pain rating is only one of many factors to consider. Tachycardia, respiratory distress, pallor, bloating, bleeding, general appearance, or hypotension that accompanies severe abdominal pain can represent shock and would place the patient at high risk. The elderly patient with severe abdominal pain presents another potential high-risk situation. Often the elderly experience bowel obstructions, gastrointestinal bleeds, and other abdominal complications associated with significantly higher morbidity and mortality than younger patients.

Several important assessment questions can help the triage nurse determine whether or not the patient meets high-risk criteria. These include the following:

- How long has the patient had the pain?
- How does the patient describe their pain?
- What made the patient come to the ED today?
- Has the patient had severe nausea, vomiting, or diarrhea?
- Has the patient had other symptoms, such as fever or loss of appetite?
- Is the patient dehydrated?

Patients with severe "ripping" abdominal pain radiating to the back are at high risk for an abdominal aortic aneurysm (AAA). Patients with an AAA describe the pain as severe, constant, and sudden in onset and may have a history of high blood pressure. Though other less imminently life-threatening diagnoses such as pancreatitis can masquerade as an AAA, it is the high-risk nature of an AAA that defines this presentation as an ESI 2.

Patients with abdominal pain are often considered ESI level 3 at the beginning of the triage interview, and after the discovery of tachycardia or other risk factors, the triage nurse may determine that the patient is indeed high-risk. This is described further in Chapter 4, "ESI Levels 3–5 and Expected Resource Needs."

Vomiting blood or a chief complaint of blood per rectum should be seriously considered and evaluated in the context of vital signs. A 30-year-old with bright red blood per rectum and normal vital signs is appropriately an emergency but does not warrant an ESI level-2 designation. All five ESI levels are appropriate for emergency care within an ED setting. However, the elderly patient who called an ambulance because he started vomiting blood and has a heart rate of 117 beats/minute and a respiratory rate of 24 breaths/minute is high-risk and does meet ESI level-2 criteria.

Cardiovascular

Chest pain is also a very common chief complaint evaluated in EDs. The presentation of acute coronary syndromes (ACS) is not always specific, and it is sometimes difficult to determine the risk of ACS at triage. Many EDs do not obtain electrocardiograms at triage. It is important to remember that unless the electrocardiogram is interpreted by a physician prior to the triage nurse assessment, it will not alter the triage nurse decision. The mere decision by the triage nurse that the patient should have an electrocardiogram can be interpreted as meaning the patient meets ESI level-2 criteria and is at high risk for cardiac ischemia. Patients who have an episode of chest or epigastric discomfort, with or without accompanying symptoms, usually will need an electrocardiogram performed rapidly to determine the presence of ACS and need to be identified as high-risk ESI level 2.

It is also important for the triage nurse to incorporate knowledge of gender differences in the presentational symptoms characteristic of heart disease. The 54-year-old obese female who presents to the ED with epigastric pain and fatigue is at risk of ACS and should be assigned to ESI level 2 –high risk.

Patients with chest pain who are physiologically unstable and require immediate interventions such as intubation or hemodynamic support should be triaged as ESI level 1. Not all chest pain patients meet level-1 or level-2 criteria. For example, a 20- year-old healthy patient with chest pain, normal oxygen saturation, cough, and fever of 38.3°C (101°F) is at low risk for ACS and does not meet ESI level-1 or level-2 criteria. But, a 20-year-old healthy patient with chest pain who tells the triage nurse he is using cocaine should be considered high-risk. Another example of a patient with chest pain that does not meet ESI level-2 criteria would be the patient with recent upper respiratory symptoms, productive cough with chest pain, and no other cardiovascular risk factors. Each patient must be assessed individually. Again, careful listening, vigilance, and experience are helpful since certain entities, including thoracic aortic dissection, can occur from childhood through adulthood.

Other potentially high-risk cardiovascular situations include hypertensive crisis, acute vascular arterial occlusions, and patients who present with a fever post valve replacement.

Nose and Throat

Patients who are drooling and/or exhibiting respiratory stridor may have impending airway loss. Although less common, epiglottitis, a foreign body (airway foreign body or esophageal foreign body in a child) and peritonsillar abscess place patients at risk for airway compromise. These are extremely high-risk patients. Patients with either of these complaints are in immediate danger of airway compromise and require immediate intervention. ESI level-1 criteria are met.

When patients with epistaxis present, the triage nurse should obtain a blood pressure, although this is not in the ESI algorithm. Epistaxis can be caused by uncontrolled high blood pressure. Several etiologies of epistaxis represent high-risk situations and include the following: brisk bleeding secondary to posterior nosebleed or in the patient using warfarin or other anti-coagulant. In these situations, patients are ESI level 2.

Environmental

Patients with inhalation injuries from closed space smoke inhalation or chemical exposure should be considered high-risk for potential airway compromise. If the patient presents with significant airway distress and requires immediate intervention, they meet the level-1 criterion.

Patients with third-degree burns should also be considered high-risk and be assigned ESI level 2. It is possible that they will require transfer to a burn center for definitive care.

General Medical

Several other general medical complaints need to be considered for possible high-risk situations. These medical complications include the following:

- Diabetic ketoacidosis
- Hyper- or hypoglycemia
- Sepsis
- Complaints of syncope or near syncope
- A variety of other electrolyte disturbances

Patients with diabetes should have a bedside test of glucose performed at triage whenever possible to identify possible hyperglycemic emergencies. If the glucose level is high, patients may be at risk for diabetic ketoacidosis or hyperosmolar hyperglycemic state. Conversely, patients may have very low glucose readings that also place them in a high-risk category. The unconscious patient with critically high or low blood sugar is considered an ESI level 1. The most common electrolyte abnormality is hyperkalemia, which is a very high-risk situation that can lead to serious cardiac dysrhythmias. Hyperkalemia might be suspected in any renal dialysis patient. Near syncope is a very common complaint that should be carefully assessed, especially in the context of patient demographics and past medical history. Finally, oncology patients with a fever are considered immunosuppressed, especially when undergoing chemotherapy. They are at risk for sepsis and should be identified as high-risk and rapidly evaluated.

Genitourinary

Renal dialysis patients unable to complete dialysis often have a variety of electrolyte disturbances that place them at high risk. Testicular torsion is another one of the life or limb, permanent time-sensitive clinical situations capable of producing permanent organ loss. Males with testicular torsion will complain of severe pain, are easily recognized, and require rapid evaluation

and surgical intervention in addition to rapid pain control. Such a patient should not be assigned to the waiting area but must be seen right away.

Mental Health

Many patients who present with mental health problems are at high risk because they may be a danger either to themselves, others, or the environment. Patients who are suicidal, homicidal, psychotic, violent, or present an elopement risk should be considered high-risk.

Intoxication without signs of trauma or associated risk of aspiration does not represent a high-risk criterion. The intoxicated patient needs to be carefully assessed for signs of trauma or behavioral issues related to alcohol use or past medical history, which could represent a high-risk situation, in other words ESI level 2.

Neurological

Patients with severe headache associated with mental status changes, high blood pressure, lethargy, fevers, or a rash should be considered high risk. Any patient with sudden onset of speech deficits or motor weakness should also be assigned ESI level 2. Patients with these symptoms may be experiencing an acute stroke and immediate evaluation is critical. Time from onset of symptoms is a critical factor in determining treatment options, in particular fibrinolytic therapies. A patient with no past medical history of headaches who presents to the emergency department with the sudden onset of a severe "worst headache of my life," should be identified as at high risk for a sub-arachnoid bleed. The patient will often describe exactly what they were doing when the headache began, typically after exertion, such as lifting, having a bowel movement, or having sexual intercourse.

Seizures are another common chief complaint. Sometimes patients arrive by ambulance and are already postictal. All patients with a reported seizure meet ESI level-2 criteria and should not wait for a prolonged period of time: they may experience another seizure.

Obstetrical and Gynecological

Females with abdominal pain or vaginal bleeding should be carefully assessed and vital signs obtained if there is no obvious life threat. Pregnancy history and last menstrual period should always be ascertained from all females of childbearing age. Patients may not recognize that they are pregnant, so the triage nurse should consider pregnancy a possibility in the assessment of female patients. In early pregnancy, the triage nurse should assess for signs and symptoms of ectopic pregnancy and spontaneous abortion. All pregnant patients with localized abdominal pain, vaginal bleeding or discharge, 14 to 20 weeks and over should be assigned ESI level 2 and seen by a physician rapidly (according to individual institutional policy). Patients with generalized cramping and bleeding with stable vital signs do not meet ESI level-2 criteria.

The triage nurse should assess for signs and symptoms of abruptio placentae and placenta previa in late pregnancy.

A postpartum patient with a chief complaint of heavy vaginal bleeding should also be assigned ESI level 2 and urgently need to be seen by a physician. Any female patient, whether pregnant or postpartum, who presents with significant hemodynamic instability and needs immediate lifesaving interventions should be triaged as ESI level 1.

Ocular

Conditions that may be associated with a chief complaint of some type of visual loss include the following:

- Chemical splash
- Central retinal artery occlusion
- Acute narrow-angle glaucoma
- Retinal detachment
- Significant trauma

A chemical splash to the eye (especially if unknown, a base, or an acid) is an immediate threat to vision that may result in permanent deficit. Chemical splashes to the eye, particularly alkali, necessitate immediate flushing to prevent further damage to the cornea. As with any immediate time-sensitive threat to life or limb, this constitutes a very high priority level-2 patient. The triage nurse should facilitate immediate irrigation regardless of bed availability.

Trauma to the eye can result in a globe rupture and hyphema. All these conditions require immediate evaluation and treatment to prevent further complications or deterioration. Patients with significant trauma to the eye or sudden partial or full loss of vision, are at high risk for permanent damage to the eye and should be triaged at ESI level 2.

Orthopedic

Patients with signs and symptoms of compartment syndrome are at high risk for extremity loss and should be assigned ESI level 2. Other patients with high-risk orthopedic injuries include any extremity injury with compromised neurovascular function, partial or complete amputations, or trauma mechanisms identified as having a high risk of injury such as serious acceleration, deceleration, pedestrian struck by a car, and gun shot or stab wound victims.

Patients with possible fractures of the pelvis, femur, or hip and other extremity dislocations should be carefully evaluated and vital signs considered. These fractures can be associated with significant blood loss. Again, hemodynamically unstable patients who need immediate lifesaving intervention such as high-level amputations meet ESI level-1 criteria.

Pediatric

It is not uncommon for the triage nurse to be uncomfortable when making triage acuity decisions about children, especially infants. It is important to obtain an accurate history from the caregiver and evaluate the activity level of the child. The child who is inconsolable or withdrawn may be at high risk of serious illness.

The following conditions are examples of high-risk situations for children:

- Seizures
- Severe sepsis, severe dehydration
- Diabetic ketoacidosis
- Suspected child abuse
- Burns
- Head trauma
- Ingestions and overdoses including vitamins
- Infant less than 30 days of age with a fever of 38°C (100.4°F) or greater
- Sickle cell crisis

See Chapter 6, “Use of the ESI for Pediatric Triage, for a detailed discussion of the use of ESI for triage of patients less than 18 years of age.

Respiratory

Many respiratory complaints place patients at high risk. Patients with mild-to-moderate distress should be further evaluated for respiratory rate and pulse oximetry to determine whether they should be categorized ESI level 2. Patients in severe respiratory distress who require immediate lifesaving intervention such as intubation meet level-1 criteria. The high-risk patient is one who is currently ventilating and oxygenating adequately but is in respiratory distress and has the potential to rapidly deteriorate. Potential etiologies of respiratory distress may include asthma, pulmonary embolus, pleural effusion, pneumothorax, foreign body aspiration, toxic smoke inhalation, or shortness of breath associated with chest pain.

Toxicological

Most patients who present with an overdose should be rapidly evaluated and represent a high-risk situation. It is often difficult to determine which drugs were taken and the quantities consumed. A patient who is apneic on arrival or requires other immediate lifesaving interventions should be categorized as ESI level 1; all other admitted overdoses should be considered ESI level 2.

Transplant

A transplant patient who comes to the ED for a nontransplant-related issue, such as a laceration to a finger, is not automatically ESI level 2. The nurse needs to assess the situation and assign the appropriate triage level. Ill patients whose status is post-organ transplant are immunocompromised and considered high-risk. They can present with organ rejection, sepsis, or other complications. Patients who are on a transplant list are also usually considered high-risk.

Trauma

Traumatic events may involve high-risk injuries that may not be immediately obvious. Any mechanism of injury associated with a high risk of injury should be categorized ESI level 2. If a trauma patient presents with unstable vital signs and requires immediate intervention, the patient should be triaged as ESI level 1. Serious injury results from the transfer of mechanical or kinetic energy and is caused by acceleration forces, deceleration forces, or both. Victims of motor vehicle and motorcycle crashes, falls, and gunshot and stab wounds are examples of blunt and penetrating trauma, which should be assessed carefully for potential for serious injury.

The triage nurse should obtain the following details regarding the injury as pertinent:

- Mechanism of injury
- When the injury occurred
- Loss of consciousness
- Head-injured patient returning/presenting with symptoms of increased intracranial pressure (headache/vomiting)
- Age of the patient
- Distance the patient fell or jumped
- How fast the vehicle was moving
- Location of penetrating injury
- Number of gunshots heard
- Type of weapon

Again, the nurse will use his or her knowledge of the bio-mechanics and mechanism of injury to assess the patient and decide whether the patient meets ESI level-2 criteria. Gunshot wounds to the head, neck, chest, abdomen, or groin usually require trauma team evaluation and immediate interventions and should be triaged using ESI criteria. If the patient requires immediate intervention, they should be triaged as ESI level 1. If the patients do not meet level-1 criteria but are high-risk, they should be triaged as ESI level 2. In EDs that are also trauma centers, trauma criteria and ESI triage criteria should be applied separately, and patients should be assigned both an ESI level and a trauma level, which may or may not be the same. For example, a patient made level 1 trauma by mechanism, who has stable vital signs and no complaints, would be ESI level 2, high-risk mechanism. This patient would not meet ESI level 1 criteria, because he or she does not require a lifesaving intervention. These circumstances are often misinterpreted by ED nurses, and it is important to stress this.

Wound Management

Several factors signal a high-risk wound. These include uncontrolled bleeding, arterial bleeding, and partial or full

amputations. Most wounds do not meet the criteria for ESI level 2. A patient with a stab wound requires careful assessment including neurovascular status. Any uncontrolled bleeding that requires immediate lifesaving intervention to stabilize the patient meets level-1 criteria.

The examples of high-risk situations above are summarized in Table 3-1.

2. Is the Patient Experiencing New Onset Confusion, Lethargy, or Disorientation?

The second question to consider when determining whether a patient meets level-2 criteria is, "Does the patient have new onset confusion, lethargy, or disorientation?" Altered mental status is another frequent chief complaint. Family members, friends, or paramedics may accompany these patients to the ED. At decision point B of the ESI algorithm, the presence of confusion, lethargy, or disorientation refers to new onset or an acute alteration in level of consciousness (LOC). Chronic dementia and chronic confusion do not meet criteria for ESI level 2. For example, if an elderly patient with dementia presents with a possible fractured hip, they do not meet level 2 criteria because the dementia is not considered to be of new onset.

Confusion, lethargy, or disorientation may be caused by a variety of serious medical conditions including stroke, transient ischemic attack, or other structural pathology to the brain, metabolic or electrolyte imbalances such as hypoglycemia or hyponatremia, or toxicological conditions. Other examples of patients who may meet ESI level 2 criteria include patients with diabetic ketoacidosis, patients experiencing an acute psychotic episode, or an otherwise healthy adult or child with new onset confusion.

This portion of the algorithm is usually very clear and leaves very little open to interpretation. If the patient's history is unknown, and the patient presents to triage confused, lethargic, or disoriented, the triage nurse should assume this condition is new and select ESI level 2 as the triage category. Again, if the patient has new onset confusion, lethargy, or disorientation and requires an immediate lifesaving intervention as previously described, the patient then meets ESI level-1 criteria (e.g., new onset confusion and difficulty maintaining an airway).

3. Is the Patient Experiencing Severe Pain or Distress?

The third and final question to address when determining whether the patient meets level-2 criteria is, "Is the patient experiencing severe pain or distress?" In 2009, the Emergency Nurses Association (ENA), the American College of Emergency Physicians (ACEP), the American Society of Pain Management Nursing (ASPMN), and the American Pain Society (APS) Board of Directors approved a joint position statement that articulated 14 core principles of optimal pain management for which EDs can strive (ACEP et al. 2010). It has since been replaced with an updated statement (ACEP et al., 2017).

Pain

The patient should be assessed for the presence of severe pain or distress. All patients who have a pain rating of 7/10 or greater should be *considered* for meeting ESI level-2 criteria. This is the second most frequently misinterpreted criteria of ESI. **Not all patients with a pain score of > 7 should be triaged as ESI level 2.** It is up to the discretion of the triage nurse to determine whether the clinical condition and pain rating in combination warrant a rating of ESI level 2. In general, it is helpful to ask, "Can I do anything at triage to help decrease the pain?" For example, a patient who had a heavy metal object fall on his toe may rate the pain a 10/10. Indeed, the patient may have a fracture and be experiencing severe pain. The patient probably has done nothing to try to relieve the pain prior to arrival in the ED. The correct triage level for this patient would be ESI level 4. Only one defined resource (remember, "resources" in the context of ESI triage refers to those items defined as a resource) will be needed (a radiograph). Of course, in addition to the defined resource, good medical care will require adequate pain relief. The triage nurse should implement comfort measures at triage including ice, elevation, and analgesics (if standing orders are in place) to reduce the pain. The triage nurse should believe the patient's pain is 10/10 and address the pain at triage. However, this patient can wait to be seen and you would certainly not use your last open bed for this patient. It is not possible to manage pain at triage for patients with renal colic, cancer, or sickle cell crisis. These patients should be triaged as ESI level 2 and rapid placement should be facilitated whenever possible.

In summary, the triage nurse assesses not only the pain intensity rating provided by the patient but also the chief complaint, past medical history, physiologic appearance of the patient, and what interventions can be provided at triage to decrease pain when determining a triage category.

Examples of patients for whom the triage nurse could use severe pain criteria to justify an ESI level-2 rating include the following:

- A patient with 10/10 rating of flank pain who is writhing at triage
- An 80-year-old female with 7/10 rating of generalized abdominal pain with severe nausea
- A 30-year-old patient in acute sickle cell pain crisis
- An oncology patient with severe pain
- Any full- or partial-thickness burn that will require immediate pain control
- Patient with acute urinary retention, more commonly male.

Table 3-1. Examples of Possible High-Risk Situations

System	Demographics, Chief Complaint	ESI 2: Yes/No Rationale
Abdomen	88-year-old female with severe right lower quadrant abdominal pain, vital signs stable	Yes. High risk for acute abdominal emergency, which is associated with high mortality in the elderly.
	22-year-old male with generalized abdominal pain, nausea, vomiting, and diarrhea for three days, vital signs stable	No. Symptoms are more indicative of gastroenteritis than an acute surgical. emergency. Patient is stable to wait.
	45-year-old female who has been vomiting blood and is tachycardic	Yes. High risk for gastrointestinal bleeding and patient can deteriorate rapidly.
	22- year-old female noticed a spot of blood on toilet paper this a.m. after having a bowel movement. Has a history of hemorrhoids.	No. This patient most likely has a hemorrhoid, and this is not a high-risk situation.
Cardiovascular	35-year-old female with a sudden onset of palpitations, anxious, heart rate of 160 beats/minute, blood pressure of 120/70 mm Hg	Yes. High risk for possible supraventricular tachycardia.
	35-year-old female with sudden onset of palpitations, anxious, heart rate of 90 beats/minute, blood pressure of 120/70 mm Hg	No. This patient may be having an anxiety attack.
	65-year-old female with sudden onset of shortness of breath and discomfort in chest for three hours	Yes. High risk for possible myocardial ischemia.
	45-year-old male with generalized fatigue, chest pain when coughing, productive cough with green sputum, fever, and chills for four days	No. This patient has classic non-cardiac symptoms, despite having chest pain.
	52-year-old male with sudden onset of pain to left foot, a history of diabetes requiring insulin therapy. Left foot is cold to touch, and the nurse is unable to palpate a pulse in the foot	Yes. High risk for acute arterial occlusion.
Eye, ENT	65-year-old female with sudden onset of loss of vision.	Yes. All complaints with sudden loss of vision are high-risk.
	22-year-old male patient with trauma to eye in a bar fight. Unable to open eye.	Yes. High risk for globe rupture or other trauma.
General medicine	40-year-old female diabetic with vomiting for two days	Yes. At high risk for diabetic ketoacidosis, which requires rapid evaluation and management.
	69-year-old male who is weak and dizzy and undergoes regular kidney dialysis	Yes. High risk for hyperkalemia and other electrolyte imbalances.
	29-year-old female with a recent history of headaches, blood pressure of 210/120 mm Hg, and no known history of high blood pressure	Yes. High risk for hypertensive emergency.
	55-year-old male with a laceration to the thumb. Blood pressure of 204/102 mm Hg, known history of high blood pressure, and admits to skipping a few doses of blood pressure medication. Denies other complaints.	No. Patient will not require emergent treatment of his blood pressure. Will require re-evaluation of his anti-hypertensive dose and agents.
Genitourinary	22-year-old male with sudden onset of severe left testicle pain	Yes. High risk for testicular torsion versus epididymitis.
	29-year-old female with a three-day history of urinary frequency and voiding in small amounts	No. This patient most likely has a tract infection, which does not require rapid evaluation.
Gynecological	24-year-old female, eight weeks pregnant, left lower quadrant abdominal pain and spotting.	Yes. High risk for possible ectopic pregnancy.
	24-year-old female with severe left lower quadrant pain abdominal. Denies vaginal bleeding.	Yes. High risk for ectopic pregnancy unless the triage nurse can confirm the absence of pregnancy.
	32-year-old female with generalized abdominal cramping and vaginal bleeding, 14 weeks pregnant, vital signs stable	No. Most likely this is a threatened abortion, which does not require emergent evaluation because of the stable vital signs.
Mental Health	19-year-old female who is combative and hostile	Yes. High risk for safety, and this patient should not be left in the waiting room.
	22-year-old male with suicidal thoughts	Yes. High risk for patient injury if left alone.

Table 3-1. Examples of Possible High-Risk Situations

System	Demographics, Chief Complaint	ESI 2: Yes/No Rationale
	35-year-old female who was brought in by the police, alcohol on breath, unsteady gait, a large laceration to head, slurred speech but oriented	Yes. High risk for a serious head injury.
	52-year-old female feeling overwhelmed and requesting a referral to counseling. Denies homicidal or suicidal thoughts. Alert, oriented, and cooperative.	No. This patient is not at high risk.
Neurological	35-year-old female with a severe headache, stiff neck, rash, temperature 38.9°C (102°F)	Yes. High risk for possible meningitis. Rapid deterioration is common.
	55-year-old male with sudden onset of worst headache of life after stressful activity	Yes. High risk for subarachnoid hemorrhage.
	52-year-old male with sudden onset of slurred speech	Yes. High risk for acute stroke.
	33-year-old male with “pins and needles” feeling to right first and second fingers for several weeks	No. Does not require rapid evaluation.
Oncologic	40-year-old female with lymphoma, currently receiving chemotherapy, temperature 39°C (102.2°F)	Yes. High risk for neutropenia and infection.
	66-year-old male with lung cancer, reports increasing shortness of breath over the past few days. Just completed chemotherapy two weeks ago.	Yes. High risk for pleural effusion, pulmonary embolus, and other emergent conditions.
	60-year-old female who cut finger while slicing a bagel. Currently receiving radiation for breast cancer.	No. Not a high-risk situation.
Pediatric	9-month-old baby with vomiting and diarrhea. She is able to drink, has a wet diaper, and is fussy and crying tears during triage.	No. While the baby may be dehydrated, this does not appear to be a high-risk situation.
	9-month-old baby with vomiting and diarrhea. She is unable to drink, has not wet a diaper for several hours, is unable to hold anything down, and has very dry mucous membranes.	Yes. This baby is at high-risk.
	6-year-old male with a sudden onset of wheezing that is audible during triage without auscultation, oxygen saturation of 97% on room air, and is in moderate respiratory distress	Yes. Moderate respiratory distress indicates a possible high risk for deterioration.
	14-day-old baby with a fever of 38.2°C (100.8°F)	Yes. Infants in the first 30 days of life with a fever greater than 38°C (100.4°F) are at high risk for bacteremia.
Respiratory	5-year-old female presents with drooling and difficulty swallowing	Yes. High risk for an airway management problem such as epiglottitis, peri-tonsillar abscess, foreign body, angioedema.
	25-year-old male with mild wheezing, oxygen saturation of 98% on room air, no obvious respiratory distress. Recent upper respiratory infection.	No. This is not a high-risk situation.
	20-year-old tall thin male with sudden onset of severe shortness of breath after coughing	Yes. Tall thin young males are at risk for spontaneous pneumothorax.
Trauma	45-year-old male involved in a motor vehicle crash immediately prior to arrival. Unable to remember the events. Moderately severe headache.	Yes. At high-risk for a traumatic brain injury and possible epidural hematoma.
	17-year-old male with a stab wound to groin, bleeding controlled.	Yes. High risk for vascular injury.
	34-year-old female involved in a low speed motor vehicle crash while driving. 32 weeks pregnant, denies complaints.	Yes. High risk for maternal and fetal injuries.
	6-year-old male fell from the top of the monkey bars today. Reports a 1- minute loss of consciousness at the time. Patient is vomiting and was sent by pediatrician for head scan.	Yes. High-risk situation.

All ED patients are to be assessed for pain and asked to rate their pain using a scale such as the visual analog scale. Many triage nurses are uncomfortable with documenting a patient's pain rating and then having the patient wait to be seen. It is important for the triage nurse to understand that the patient's self-reported pain rating is only one piece of the pain assessment. Triage nurses should assign ESI level 2 if the patient reports a pain rating of 7/10 or greater and the triage nurse's subjective and objective assessment confirms that the patient's pain requires interventions that are beyond the scope of triage. The triage nurse concludes that it would be inappropriate for this patient to wait and would assign this patient to the last open bed.

Distress

Finally, in determining whether a patient meets ESI level-2 criteria, the triage nurse must assess for severe distress, which is defined as either physiological or psychological. In addition to pain, patients experiencing severe respiratory distress meet criteria for ESI level 2 for physiological disturbances.

Examples of severe psychological distress include patients who are:

- Distraught after experiencing a sexual assault
- Exhibiting behavioral outbursts at triage
- Combative
- Victims of domestic violence
- Experiencing an acute grief reaction
- Suicidal and a flight risk (this patient also meets high-risk criteria)

These are patients that the triage nurse usually prefers to have placed in the treatment area immediately to address the acute issue expeditiously. Additionally, this will serve to prevent persons in the waiting room from becoming agitated.

Special Situations

Many EDs now have special alert processes that initiate a team approach to a specific time-sensitive problem. Clinical syndrome responses therefore may include immediate activation of alerts such as myocardial infarction alert, stroke alert, sepsis alert, and trauma alert. These are hospital-specific, protocol-driven responses. Patients that qualify for alert activation are automatically high-risk and therefore classified at least ESI 2. For example, a patient may present to triage awake, alert, and oriented, complaining of left-sided weakness. the patient does not meet ESI level-1 criteria but is at high risk for a stroke. This patient meets ESI level-2 criteria. If deteriorating or in extremis, the patient would be classified as ESI level 1.

Summary

We have reviewed the key components and questions that need to be answered to determine whether a patient meets ESI level-2 criteria. It is critical that the triage nurse consider these questions as he or she triages each patient. Missing a high-risk situation may result in an extended waiting period and potentially negative patient outcomes. Many high-risk situations have not been discussed and are beyond the scope of this handbook. With ESI level 2, the role of the triage nurse is to gather subjective and objective information from the patient, analyze it, and decide whether this patient has a high-risk situation.

NOTE Appendix A of this handbook includes frequently asked questions and posttest assessment questions for Chapters 2 through 8. These sections can be incorporated into a locally developed ESI training course.

References

American College of Emergency Physicians, American Academy of Emergency Nurse Practitioners, Emergency Nurses Association, & Society of Emergency Medicine Physician Assistants. (2017). *Optimizing the treatment of acute pain in the emergency department* [Joint Policy Statement]. https://www.acep.org/globalassets/new-pdfs/policy-statements/optimizing-the-treatment-of-acute-pain-in-the-ed.pdf

American College of Emergency Physicians, American Pain Society, American Society for Pain Management Nursing, & Emergency Nurses Association. (2010). Optimizing the treatment of pain in patients with acute presentations [Joint Position Statement]. *Annals of Emergency Medicine*, *56*(1), 77–79. https://doi.org/10.1016/j.annemergmed.2010.03.035

Sweet, V. (Ed.). (2018). *Emergency Nursing Core Curriculum* (7th ed.). Elsevier.

4

ESI Levels 3–5 and Expected Resource Needs

Traditionally, comprehensive triage has been the dominant model for triage acuity assignment in U.S. emergency departments (Gilboy, 2010; Gilboy et al., 1999). Triage acuity rating systems have been based solely on the acuity of the patient, determined through the nurse's assessment of vital signs, subjective and objective information, past medical history, allergies, and medications. Such systems require the nurse to assign an acuity level by making a judgment about how sick the patient is and how long the patient can wait to be seen by a provider.

The ESI triage system uses a novel approach that includes not only the nurse's judgments about who should be seen first but also, for less acute patients (those at ESI levels 3 through 5), calling on the nurse to add predictions of the resources that are likely to be used to make a disposition for the patient.

This chapter includes background information on the inclusion of resource predictions in the ESI and a description of what constitutes a resource. Examples are given of patients rated as levels 3, 4, and 5 and the resources that each patient is predicted to need.

Resource Predictions

Estimation of resource needs begins only after it has been determined that the patient does not meet ESI level 1 or 2 criteria. The nurse then predicts the number of resources a patient will need in order for a disposition to be reached. When Wuerz and Eitel created the ESI triage system, they included resource utilization to provide additional data and enable a more accurate triage decision. They believed that an experienced emergency department (ED) triage nurse would be able to predict the nature and number of tests, therapeutic interventions, and consultations that a patient would need during his/her ED stay. Studies of ESI implementation and validation have verified that triage nurses are able to predict ED patients' resource needs (Eitel et al., 2003; Tanabe, Gimbel Yarnold, & Adams, 2004). One study was conducted at seven EDs representing varied regions of the country, urban and rural areas, and academic and community hospitals. Nurses were able to predict how many ESI-defined resources the ED patients required 70 percent of the time. That is, experienced triage nurses can reasonably predict at triage how many resources patients will require to reach ED disposition; more importantly, they can discriminate at presentation low versus high intensity patients. This differentiation by resource requirements enables much more effective streaming of patients at ED presentation into alternative operational pathways within the ED, that is, the parallel processing of patients. Research has also established that ESI triage levels correlate with important patient outcomes, including admission and mortality rates (Eitel et al., 2003).

Figure 4-1. ESI Triage Algorithm, v4

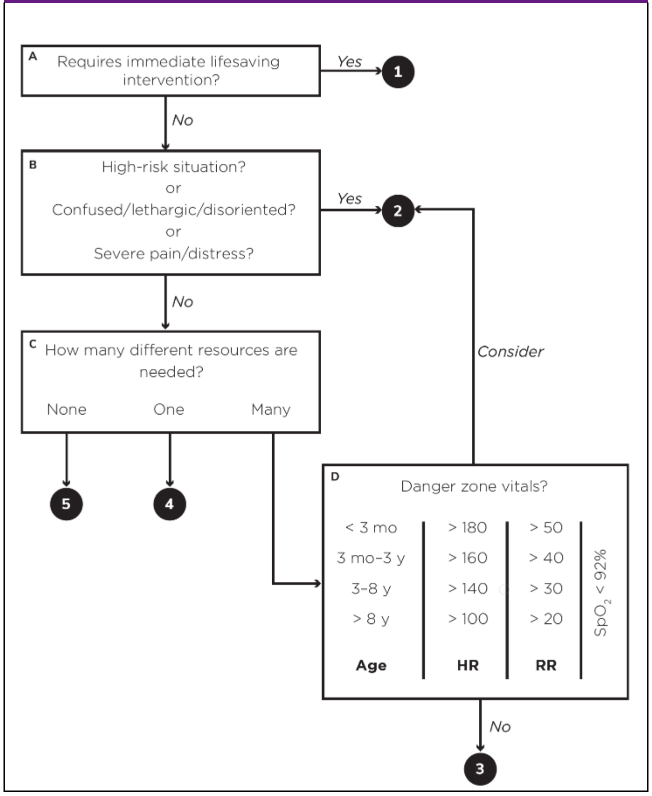

Again, it is important to note that *resource prediction is only used for less acute patients*. At decision points A and B on the ESI

algorithm (Figure 4-1), the nurse decides which patients meet criteria for ESI levels 1 and 2 based only on patient acuity.

However, at decision point C, the nurse assigns ESI levels 3 to 5 by assessing both acuity and predicted resource needs. Thus, the triage nurse only considers resources when the answers to decision points A and B are "no."

To identify ED patients' resource needs, the triage nurse must have familiarity with general ED standards of care and specifically with what constitutes prudent and customary emergency care. An easy way to think about this concept is to ask the question, "Given this patient's chief complaint, what resources are the emergency providers likely to utilize?" Another way to look at this is to consider, "What is it going to take for a disposition to be reached?" Disposition can be admission, discharge, or transfer.

The triage nurse uses information from the brief subjective and objective triage assessment – as well as past medical history, medications, age, and gender – to determine how many different resources will be needed for the ED provider to reach a disposition. For example, a healthy teenage patient with a simple leg laceration and no prior medical history would need only one resource: suturing. On the other hand, an older adult with multiple chronic medical problems and no history of dizziness who presents with a head laceration from a fall will clearly need multiple resources: suturing, blood/urine tests, ECG, head CT, or consultations with specialists. Accurate use of ESI triage is contingent on the nurse's ability to accurately predict resources and as such is best performed by an experienced emergency nurse.

What Constitutes a Resource?

Guidelines for the categorization of resources in the ESI triage system are shown in Table 4-1. ESI levels 3, 4, and 5 are differentiated by the nurse's determination of how many different resources are needed to make a patient disposition. On the basis of the triage nurse's predictions, patients who are expected to consume no resources are classified as ESI level 5, those who are likely to require one resource are ESI level 4, and those who are expected to need two or more resources are designated as ESI level 3. Patients who need two or more resources have been shown to have higher rates of hospital admission and mortality and longer lengths of stay in the ED (Eitel et al., 2003; Tanabe, Gimble, Yarnold, Kyriacou, & Adams, 2004).

Though the list of resources in Table 4-1 is not exhaustive, it provides general guidance on the types of diagnostic tests, procedures, and therapeutic treatments that constitute a resource in the ESI system. Emergency nurses who use the ESI are cautioned not to become overly concerned about the definitions of individual resources. It is important to remember that ESI requires the triage nurse to merely estimate resources

Table 4-1. Resources for the ESI Triage System

Resources	Not Resources
• Labs (blood, urine)<br>• Electrocardiogram, radiographs<br>• Computed tomography, magnetic resonance imaging, ultrasound, angiography	• History and physical exam (including pelvic)<br>• Point-of-care testing
• Intravenous fluids (hydration)	• Saline or heparin lock
• Intravenous, intramuscular, or nebulized medications	• Oral medications<br>• Tetanus immunization<br>• Prescription refills
• Specialty consultation	• Phone call to primary care physician
• Simple procedure = 1 (laceration repair, urinary catheter)<br>• Complex procedure = 2 (procedural sedation)	• Simple wound care (dressings, recheck))<br>• Crutches, splints, slings

that ESI requires the triage nurse to merely estimate resources that the patient will need while in the ED.

The most common resources are listed in Table 4-1. However, a comprehensive list of every possible ED resource is neither practical nor necessary. In fact, all that is really necessary for accurate ESI rating is to predict whether the patient will need no resources, one resource, or two or more resources. Once a triage nurse has identified two probable resources, there is no need to continue to estimate resources. Counting beyond two resources is not necessary.

The Utility of Estimating ESI Resources

The essence of the ESI resource component is to separate more complex (resource-intensive) patients from those with simpler problems. The interventions considered as resources for the purposes of ESI triage are those that indicate a level of assessment or procedure beyond an exam or brief interventions by ED staff and/or involve personnel outside of the ED. Resources that require significant ED staff time (such as intravenous medication administration or chest tube insertion) and those that require staff or resources outside the ED (such as radiographs by the radiology staff or surgical consults) increase the patient's ED length of stay and indicate that the patient's complexity, and, therefore, triage level is higher.

Common Questions

There are some common questions about what is considered an ESI resource. One question often asked is about the number of blood or urine tests and radiographs that constitute a resource. In the ESI triage method, the triage nurse should count the

number of different types of resources needed to determine the patient's disposition, not the number of individual tests:

- A complete blood count and electrolyte panel comprise one resource (lab test).
- A complete blood count and chest radiograph are two resources (lab test, radiograph).
- A complete blood count and a urinalysis are both lab tests and together count as only one resource.
- A chest radiograph and abdominal radiograph are one resource (radiograph).
- Cervical-spine films and a computed tomography scan of the head are two resources (radiograph and computed tomography scan).

It is important for emergency nurses to understand that not every intervention they perform can be counted as a resource. For example, crutch walking education, application of a sling and swath, or application of a knee immobilizer all take time but do not count as a resource. If, for example, a splint did count, patients with sprained ankles would be triaged as ESI level 3 (radiograph and splint application). While the application of a splint can certainly take time, it is important to remember the only purpose of resource prediction with ESI is to sort patients into distinct groups and help get the right patient to the right area of the ED. Another example is a patient with a laceration who may require suturing and a tetanus booster. If a tetanus booster (intramuscular medication) "counted," any patient with a laceration who needed suturing and a tetanus booster would meet ESI level 3 criteria. In many EDs, ESI level-3 patients are not appropriate for a fast track or urgent care area. *Remember, triage level is not a measure of total nursing workload intensity; it is a measure of presentational acuity*.

Another common question about ESI resources relates to the fact that eye irrigation is also considered a resource. Patients with a chemical splash usually meet ESI level-2 criteria because of the high-risk nature of the splash, so eye irrigation is not a key factor in their ESI rating. However, if the eye problem were due to dust particles in the eye, the patient would not necessarily be high risk. In this type of patient, the eye irrigation would count as a resource and the patient would meet ESI level-4 criteria. The eye exam does not count as a resource because it is considered part of the physical exam.

Another frequent question posed by clinicians is related to the items listed as "not resources" in Table 4-1. The purpose of the list is to assist triage nurses with quick, accurate sorting of patients into five clinically distinct levels (Wuerz et al., 2000). As such, items listed as not being resources include physical exams, point-of-care tests, and interventions that tend not to lead to increased length of stay in the ED or indicate a higher level of complexity. Since the standard of care is that all ED patients undergo a basic history and physical exam, an exam does not constitute a resource for ESI classification. For the female patient with abdominal pain, a pelvic exam would be part of the basic physical exam. A patient with an eye complaint would need a slit lamp exam as part of the basic physical exam. The strength of the ESI is its simplicity; the true goal of the resource determination is to differentiate the more complicated patients needing two or more resources (level 3 or above) from those with simpler problems who are likely to need fewer than two resources (level 4 or 5). Emergency nurses should not try to complicate ESI by concentrating overly on resource definitions. Usually, a patient requires either no resources, one resource, or two or more resources.

Resources in Context

Though resource consumption may vary by site, provider, and even individual patient, triage nurses are urged to make the ESI resource prediction by thinking about the common approaches to the most common presenting problems. Ideally, a patient presenting to any emergency department should consume the same general resources. For example, a provider seeing a hemodynamically stable 82-yearold nursing home resident who has an in-dwelling urinary catheter and a chief complaint of fever and cough will most likely order blood and urine tests and a chest radiograph. The triage nurse can accurately predict that the patient needs two or more resources and therefore classify the patient as ESI level 3.

There may be minor variations in operations at different EDs, but this will rarely affect the triage rating. For example, some departments do pregnancy tests in the ED (point of care testing is not a resource according to the ESI) and others send them to the lab (a resource according to the ESI). However, patients rarely have the pregnancy test as their only resource, so most of those patients tend to have two or more resources in addition to the pregnancy test. One ED practice variation that may result in different ESI levels for different sites is the evaluation of patients with an isolated complaint of sore throat. At some hospitals it is common practice to obtain throat cultures (one resource, ESI level 4), while at others it is not (no resources, ESI level 5). Evidence-based practice guidelines are being used more and more to determine the need for radiographs or other interventions. One example is the use of the Ottawa Ankle Rules. These are validated rules used to determine the need for a radiograph of the ankle for patients that present with ankle injuries. Institutional adoption of these rules into practice varies. Institutions that use these rules at triage may obtain fewer radiographs when compared with institutions that do not routinely use these rules.

When counting resources the triage nurse should not consider which physician, nurse practitioner, or physician's assistant is working. There are practice differences among providers, but the triage nurse has to focus on what is prudent and customary.

Temperature is an important assessment parameter for determining the number of resources for very young children.

Table 4-2. Examples of Resources for ESI Levels 3–5

Scenario	Predicted Resources *	ESI Triage Category
Right lower quadrant pain:<br>22-year-old male, right lower quadrant abdominal pain since early this morning, also nausea, and no appetite.	*ESI Resources = 2 or more*<br>Exam<br>*Laboratory studies*<br>*Intravenous fluid*<br>*Abdominal computed tomography scan*<br>*Surgery consult (possible)*	3
Left lower leg pain<br>45-year-old obese female with left lower leg pain and swelling which started two days ago after driving in a car for 12 hours.	*ESI Resources = 2 or more*<br>Exam<br>*Laboratory studies*<br>*Lower extremity non-invasive vascular studies*<br>*Anticoagulant therapy (possible)*	3
Ankle injury:<br>Healthy, 19-year-old female who twisted her ankle playing soccer. Edema at lateral malleolus, hurts to bear weight.	*ESI Resources = 1*<br>Exam<br>*Ankle radiograph*<br>Ace wrap<br>Crutch-walking instruction	4
Urinary tract infection symptoms:<br>Healthy, 29-year-old female with urinary symptoms, appears well, afebrile, denies vaginal discharge.	*ESI Resources = 1*<br>Exam<br>*Urine and urine culture*<br>*Urine hCG (possible)*<br>Prescriptions	4
Poison ivy:<br>Healthy 10-year-old child with rash on extremities.	*ESI Resources = none*<br>Exam<br>Prescription	5
Prescription refill:<br>Healthy 52-year-old who ran out of blood pressure medication yesterday. BP 150/84 mm Hg. No acute complaints	*ESI Resources = none*<br>Exam<br>Prescription	5

* ESI resources are listed in italic

This subject will be covered in Chapter 5, "The Role of Vital Signs in ESI Triage."

From a clinical standpoint, ESI level 4 and 5 patients are stable and can wait several hours to be seen by a provider. However, from a customer service standpoint, these patients are perhaps better served in a fast-track or urgent care area. Mid-level practitioners with the appropriate skills mix and supervision can care for level-4 and level-5 patients. With ESI, level-5 patients can sometimes be "worked in" for a quick exam and disposition by the provider, even if the department is at capacity. Often triage policies clearly state ESI level-4 or level-5 patients can be triaged to an urgent care or fast-track area.

Summary

In summary, the ESI provides an innovative approach to ED triage with the inclusion of predictions about the number of resources needed to make a patient disposition. Consideration of resources is included in the triage level assignment for ESI level-3, -4, and -5 patients, while ESI level-1 and -2 decisions are based only on patient acuity. Examples of ESI level-3, -4, and -5 patients are presented in Table 4-2. Practical experience has demonstrated that resource estimation is very beneficial in helping sort the large number of patients with non-acute presentations. Common questions about resources are addressed in the "Frequently Asked Questions" subsection of the "Chapter 4" section of Appendix A, "Frequently Asked Questions and Post-Quiz Materials for Chapters 2–8."

> **NOTE** Appendix A, "Frequently Asked Questions and Post-Quiz Materials for Chapters 2–8," includes frequently asked questions and posttest assessment questions for Chapters 2 through 8. These sections can be incorporated into a locally developed ESI training course.

References

Eitel, D. R., Travers, D. A., Rosenau, A., Gilboy, N., & Wuerz, R. C. (2003). The Emergency Severity Index version 2 is reliable and

valid. *Academic Emergency Medicine, 10*(10), 1079–1080. https://doi.org/10.1197/S1069-6563(03)00350-6

Gilboy, N. (2010). Triage. In P. K. Howard & R. A. Steinmann (Eds.). *Sheehy's Emergency Nursing: Principles and Practice* (6th ed., 59–72). Mosby.

Gilboy, N., Travers, D. A., & Wuerz, R. C. (1999). Re- evaluating triage in the new millennium: A comprehensive look at the need for standardization and quality. *Journal of Emergency Nursing, 25*(6), 468 –473. https://doi.org/10.1016/s0099-1767(99)70007-3

Tanabe, P., Gimbel, R., Yarnold, P. R., & Adams, J. G. (2004a). The Emergency Severity Index (version 3) 5-level triage system scores predict ED resource consumption. *Journal of Emergency Nursing*, *30*(1), 22–29. https://doi.org/10.1016/j.jen.2003.11.004

Tanabe, P., Gimbel, R., Yarnold, P. R., Kyriacou, D. N., & Adams, J. G. (2004b). Reliability and validity of scores on the emergency severity index version 3. *Academic Emergency Medicine*, *11*(1), 59–65. https://doi.org/10.1197/j.aem.2003.06.013

Wuerz, R., Milne, L. W., Eitel, D. R., Travers, D., & Gilboy, N. (2000). Reliability and validity of a new five-level triage instrument. *Academic Emergency Medicine*, *7*(3), 236–242. https://doi.org/10.1111/j.1553-2712.2000.tb01066.x

5

The Role of Vital Signs in ESI Triage

In this chapter, we focus on decision point D – the patient's vital signs. To reach this point in the ESI algorithm, the triage nurse has already determined that the patient does not meet ESI level-1 or level-2 criteria and that he or she will require two or more resources. Since the patient requires two or more resources, he or she meets the criterion for at least an ESI level 3. It is at this point in the algorithm that vital signs data are considered and the triage nurse must assess the patient's heart rate, respiratory rate, oxygen saturation, and, for children under age 3, temperature (see Chapter 6, "Use of the ESI for Pediatric Triage," for more detailed information concerning the use of ESI for pediatric triaging). If the danger zone vital sign limits (as illustrated in decision point D, Figure 5-1) are exceeded, the triage nurse must strongly consider uptriaging the patient from a level 3 to a level 2.

It is up to the experienced triage nurse to determine whether the patient meets criteria for ESI level 2, based on his or her past medical history, current medications, and subjective and objective assessment that includes general appearance. This decision is based on the triage nurse's clinical judgment and knowledge of normal vital sign parameters for all ages and the influence of other factors such as medications, past medical history, and pain level.

Are Vital Signs Necessary at Triage?

Prior to the advent of five-level triage in the United States, tradition dictated that every patient presenting to an emergency department should have a set of vital signs taken before triage-level assignment. Vital signs were considered an integral component of the initial nursing assessment and were often used as a decision-making tool. In a traditional three-level triage system, vital signs helped determine how long a patient could wait for treatment (i.e., if no abnormal vital signs were present, in many cases, the patient could wait a longer period of time). Essentially, ESI level-1 and level-2 patients often are taken to an area where they receive immediate staff attention prior to the point in triage when vital signs would normally be taken.

Figure 5-1. Danger Zone Vital Signs

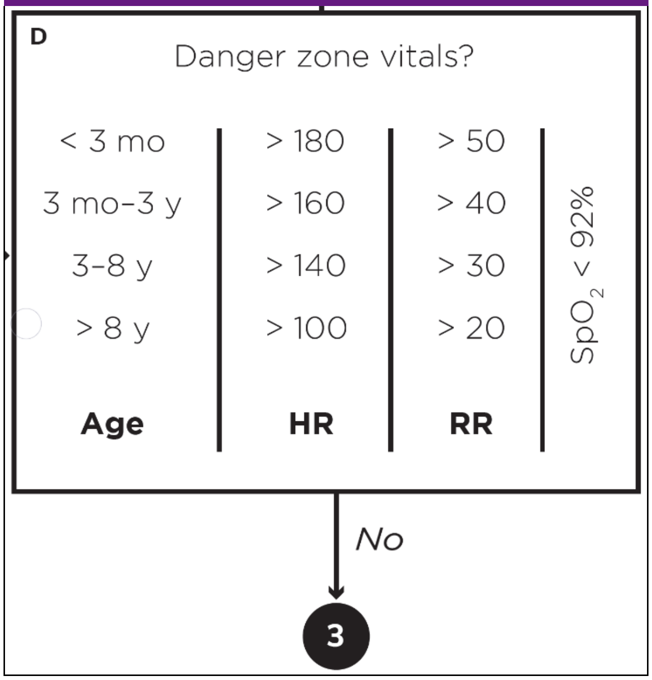

There is frequently discussion about why vital signs are not a more important part of ESI criteria. Vital signs are important; however, they are not always helpful in determining an initial triage level. An objective assessment of the patient, including the patient's chief complaint, is often sufficient to categorize the patient as a high-acuity patient (ESI level 1 or 2), or low-acuity patient (ESI level 3, 4, or 5). However, the ESI Triage Research Team recommends obtaining a full set of vital signs at triage, including temperature, heart and respiratory rates, and blood pressure. Nurses are accustomed to this practice, and we have found that when vital signs are not obtained at triage, in particular for lower acuity chief complaints, they may never be obtained during the ED stay. Furthermore, automatic blood pressure cuffs and pulse monitors rapidly accomplish this task in most emergency departments.

At the turn of the century, newer triage models, including ESI, began to advocate selective use of vital signs at triage (Gilboy et

al., 1999). Initial vital signs are not a mandatory component of other five-level triage systems and in general are not reported during the triage phase of a level-1 or level-2 patient (i.e., those patients with the highest acuity). For example, the *Guidelines for Implementation of the Australasian Triage Scale in Emergency Departments* states that "Vital signs should only be measured at triage if required to estimate urgency, or if time permits" (Australasian College for Emergency Medicine, 2016, p. 2). Similarly, the Canadian Triage and Acuity Scale upholds the need for vital signs if, and only if, they are necessary to determine a triage level (in the cases of levels 3, 4, and 5) as time permits (Beveridge et al., 1998). The Manchester Triage Group uses specific vital-sign parameters as discriminators within a presentational flow chart. The vital-sign parameter is one of the factors that help the triage nurse assign an acuity level.

Vital signs are not always the most informative method for determining triage acuity. At least one study has suggested that vital signs are not always necessary in the initial assessment of the patient at triage. In 2002, Cooper et al. examined the use of vital signs to determine a patient's triage status. They considered age and communication ability as factors. Twenty-four different U.S. emergency departments and more than 14,000 patients participated in that study. Final results demonstrated that vital signs changed the level of triage acuity status in only eight percent of the cases. When further examining individual age groups, pediatric patients aged 2 or younger showed the largest variation in triage decision with an 11.4 percent change once vital signs were collected.

Using Vital Signs with ESI Triage

Using ESI triage, the only absolute requirement for vital signs assessment at triage is for patients who meet level-3 criteria. Vital sign assessment at triage is optional for patients triaged as ESI level 1, 2, 4, or 5. While the ESI system does not require vital signs assessment on all patients who present to triage, local policies may dictate a different procedure. Factors such as staffing levels, case mix, and local resources influence individual hospital policies regarding vital signs at triage and are beyond the scope of this handbook. In general, when triaging a stable patient, it is never wrong to obtain a set of vital signs, unless you delay placement to obtain vital signs. The developers of the ESI and the current ESI research team believe that experienced ED nurses can use vital sign data as an adjunct to sound clinical judgment when rating patients with the ESI. There is limited evidence that abnormal vital signs predict serious illness. The ESI has been revised over time to reflect changes in the available evidence and recommendations from the literature. The ESI working group initially used the systemic inflammatory response syndrome (SIRS) literature (Rangel-Frausto et al., 1995) in developing the danger zone vital sign box and accompanying footnotes.

The first version of the ESI used the SIRS criteria to include a heart rate of greater than 90 (for adults) as an absolute indicator to uptriage from ESI level 3 to level 2 (Wuerz, Milne, Eitel, Travers, Gilboy, 2000). The SIRS research was based on predictors of mortality in an intensive care unit population. Based on an excess of false positives using these criteria for ED patients at the initial ESI hospitals, the heart rate cutoff was changed to 100 in ESI version 2, and nurses were instructed to consider uptriage to ESI 2 for adult patients with heart rates greater than 100 (Gilboy et al., 2003; Wuerz et al., 2001). Additionally, pediatric vital signs were added to the danger zone vital signs box (American College of Emergency Physicians [ACEP], 2003).

When using ESI as a triage system, vital signs assessment is not necessary in the triage area for patients who are immediately categorized as level 1 or 2. If the patient appears unstable or presents with a chief complaint that necessitates immediate treatment, then transport of the patient directly to the treatment area should be expedited. For these patients, the resuscitation team is responsible for obtaining and monitoring vital signs at the bedside. This would include patients that have clinical appearances that indicate high risk or need for immediate cardiovascular or respiratory intervention. These patients may appear pale, diaphoretic, or cyanotic. The triage nurse always has the option to perform vitals in the triage area if an open bed is not immediately available or if he or she feels that the vital signs may assist in confirming the triage acuity level.

Some patients may not be identified initially as ESI level 1 until vital signs are taken. For example, an awake, alert elderly patient who complains of dizziness might be found to have a life-threatening condition when a heart rate of 32 or 180 is discovered during vital sign measurement. In this case, the patient should be assigned ESI level 1 no matter how "good" the patient appears.

As shown in the ESI algorithm in Chapter 2, "Overview of the Emergency Severity Index," if patients do not meet ESI level-1 or level-2 criteria, the triage nurse comes to decision point C. The nurse then determines how many resources the patient is expected to need in the ED. If the patient is expected to need one or no defined resources, he or she can be assigned an ESI level of 4 or 5 and no vital sign assessment is necessary. But if the patient is expected to need two or more resources, then the nurse comes to decision point D and vital signs should be assessed.

Vital signs can play a more important role in the evaluation of some patients at triage, especially those triaged as ESI level 3. The range of vital signs may provide supporting data for potential indicators of serious illness. *If any of the danger zone vital signs are exceeded, it is recommended that the triage nurse consider up-triaging the patient from level 3 to level 2.*

Figure 5-2. Danger Zone Vital Signs

E. Danger Zone Vital Signs

Consider uptriage to ESI 2 if any vital sign criterion is exceeded.

Pediatric Fever Considerations

1–28 days of age: Assign at least ESI 2 if temp > 38°C (100.4°F).

1–3 months of age: Consider assigning ESI 2 if temp > 38°C (100.4°F).

3 months–3 years of age: Consider assigning ESI 3 if temp > 39°C (102.2°F), incomplete immunizations, or no obvious source of fever.

Table 5-1. ESI Pediatric Temperature Criteria

Age	Temperature	ESI level
1–28 days	Fever over 38°C (100.4°F)	2
1–3 months	Fever over 38°C (100.4°F)	Consider 2
3–36 months	Fever over 39°C (102.2° F)	Consider 3 (see text)

Vital signs explicitly included in ESI triage include heart rate, respiratory rate, and oxygen saturation (for patients with potential respiratory compromise). *Temperature is specifically used in ESI triage for children under age 3.* It is important to note that when considering abnormal vital signs, blood pressure is not included in the ESI algorithm. This does not mean that the triage nurse should not take a blood pressure or a temperature on older children or adults but that these vital signs are not necessarily helpful in selecting the appropriate triage acuity level.

Vital Signs and Pediatric Fever

As shown in Figure 5-2, note D of the ESI algorithm addresses pediatric fever considerations for ESI triage. This section incorporates recommendations from the American College of Emergency Physicians' *Clinical Policy for Children Younger Than Three Years Presenting to the Emergency Department with Fever* (ACEP, 2003), reapproved in 2009 by ACEP Board of Directors.

The ESI Triage Research Team recommends that vital signs in all patients under age 3 be assessed at triage. For patients in this age group, vital sign evaluation, including temperature measurement, is essential to the overall assessment (Baraff, 2000). This helps to differentiate ESI level-2 and level-3 patients and minimize the risk that potentially bacteremic children will be sent to an express care area or otherwise experience an inappropriate wait. Remember, if a patient is in immediate danger or high risk, he or she will be assigned to either ESI level 1 or 2.

Table 5-1 provides direction for the triage nurse in using the ESI to assess the febrile child and determine the most appropriate triage level. The generally accepted definition of fever is a rectal temperature greater than 38°C (100.4°F) (ACEP, 2003; Baraff et al., 1993). The infant less than 28 days old with a fever should be considered high risk and assigned to at least ESI level 2. There are no clear guidelines for the infant between 28 days and 3 months of age. The ESI research team recommends triage nurses rely on local hospital guidelines. We suggest that the nurse consider assigning at least an ESI level 2 for such patients.

Version 4 of the ESI incorporates a different set of pediatric fever guidelines for children ages 3–36 months. These pediatric fever considerations pertain to highly febrile children, defined as those with a fever greater than 39°C (102.2° F) (ACEP, 2003). When triaging a child between 3 and 36 months of age who is highly febrile, it is important for the triage nurse to assess the child's immunization status and whether there is an identifiable source for the fever.

The patient with incomplete immunizations or with no identifiable source for the fever should be assigned to at least ESI level 3. If the patient has an identifiable source for the fever and his or her immunizations are up to date, then a rating of 4 or 5 is appropriate. For example, a 7-month-old who is followed by a pediatrician, has had the Haemophilus influenza type b vaccine, and presents with a fever and pulling on his ear could be assigned to an ESI level 5.

Case Examples

The following cases are examples of how vital signs data are used in ESI triage.

"My doctor told me I am about six weeks pregnant, and now I think I am having a miscarriage," reports a healthy looking 28-year-old female. "I started spotting this morning, and now I am cramping." No allergies; no past medical history; medications: prenatal vitamins. Vital signs: T 36.7°C (98°F), HR 112 beats/minute, RR 22 breaths/minute, BP 90/60 mm Hg.

This patient meets the criteria for being uptriaged from level 3 to level 2 based on her vital signs. Her increased heart rate, respiratory rate, and decreased blood pressure are a concern. These factors could indicate internal bleeding from a ruptured ectopic pregnancy.

"The baby has had diarrhea since yesterday. The whole family has had that stomach bug that is going around," reports the mother of a 15-month-old. She tells you the baby has had a decreased appetite, a low-grade temperature, and numerous liquid stools. The baby is sitting quietly on the mother's lap. The triage nurse notes signs of dehydration. No past medical history, no known drug allergies, no medications. Vital signs: T 38°C (100.4° F), HR 178 beats/minute, RR 48 breaths/minute, BP 76/50 mm Hg.

Prior to vital sign assessment, this baby meets the criteria for ESI level 3. Based on vital sign assessment, the triage nurse should uptriage him to an ESI level 2. For a baby this age, both heart rate and respiratory rate criteria are met.

"I need to see a doctor for my cough. I just can't seem to shake it. Last night I didn't get much sleep because I was coughing so much. I am just so tired," reports a 57-year-old female. She tells you that she had a temperature of 101°F (38.3°C) last night and that she is coughing up this yellow stuff. Her history includes a hysterectomy three years ago; she takes no medications but is allergic to penicillin. Vital signs: T 38.5°C (101.4°F), RR 36 breaths/minute, HR 100 beats/minute, SpO_2 90%.

At the beginning of her triage assessment, this patient sounds as though she could have pneumonia. She will need two or more resources, but her low oxygen saturation and increased respiratory rate are a concern. After assessing vital signs, the triage nurse should uptriage the patient to an ESI level 2.

A 34-year-old obese female presents to triage complaining of generalized abdominal pain (pain scale rating: 6/10) for two days. She has vomited several times and states her last bowel movement was three days ago. She has a history of back surgery, takes no medications, and is allergic to peanuts. Vital signs: T 36.5°C (97.8°)F, HR 104 beats/minute, RR 16 breaths/minute, BP 132/80 mm Hg, SpO_2 99%.

This patient will need a minimum of two or more resources: lab, intravenous fluids, perhaps intravenous medication for nausea, and a CT scan. The triage nurse would review the patient's vital signs and consider the heart rate. The heart rate falls just outside the accepted parameter for the age of the patient but could be due to pain or exertion. In this case, the decision should be to assign the patient to ESI level 3.

A tearful 9-year-old presents to triage with her mother. She slipped on an icy sidewalk and injured her right forearm. The forearm is obviously deformed but has good color, sensation, and movement. The mother reports she has no allergies, takes no medications, and is healthy. Vital signs: BP 100/68 mm Hg, HR 124 beats/minute, RR 32 breaths/minute, and SpO_2 99%.

This child is experiencing pain from her fall and is obviously upset. She will require at least two resources: radiographs and an orthopedic consult, and perhaps procedural sedation. Her heart rate and respiratory rate are elevated, but the triage nurse should feel comfortable assigning this patient to ESI level 3. Her vital sign changes are likely due to pain and distress.

A 72-year-old patient presents to the ED with oxygen via nasal cannula for her advanced chronic obstructive pulmonary disease. She informs the triage nurse that she has an infected cat bite on her left hand. The hand is red, tender, and swollen. The patient has no other medical problems, uses albuterol as needed, takes an aspirin daily, and has no known drug allergies. Vital signs: T 37.5°C (99.6°F), HR 88 beats/minute, RR 22 breaths/minute, BP 138/80 mm Hg, SpO_2 91%. She denies respiratory distress.

This patient will require two or more resources: labs and intravenous antibiotics. She meets the criteria for ESI level 3. The triage nurse notices that her oxygen saturation and respiratory rate are outside the accepted parameters for the adult, but this patient has advanced chronic obstructive pulmonary disease. These vital signs are not a concern, so the patient should not be uptriaged but will stay at ESI level 3. If this patient had any type of respiratory complaint, she should be uptriaged to ESI level 2 due to the low SpO_2, which may or may not be normal for this particular patient.

A 25-year-old patient presents to the ED triage nurse with a chief complaint of nausea, fever, chills, and sore throat for several days, associated with decreased ability to take fluids. He denies any past medical history or taking any medications. Vital signs: T 39°C (102.3°F), HR 124 beats/minute, RR 20 breaths/minute, BP 125/80 mm Hg, SpO_2 99% on room air.

This patient will require two or more resources: intravenous fluids and medications. His HR exceeds vital sign parameters; however, this is most likely due to his fever. He should not be uptriaged and should be assigned ESI level 3. The triage nurse should administer an antipyretic at triage if the ED has such a policy.

A 19-year-old patient arrives by ambulance having an anxiety attack. She was in court and began to feel lightheaded and dizzy; the paramedics were called. Upon arrival she is hyperventilating, crying, and unable to speak in sentences. She also states she has not felt well recently and has nausea and vomiting. She denies any past medical history. Vital signs: T 37°C (98.6°F), HR 108 beats/minute, RR 40 breaths/minute, BP 130/80 mm Hg, SpO_2 100% on room air.

This patient may require two or more resources: intravenous fluids and medications. While her HR and RR exceed vital sign criteria, she should be triaged as ESI level 3. The triage nurse

would not give this patient the last monitored bed as she is stable to wait. The nurse should assist the patient in slowing down her breathing.

Summary

The information in this chapter provides a foundation for understanding the role of vital signs in the Emergency Severity Index triage system. We addressed the special case of patients under 36 months of age. Further research is necessary to clarify the best vital sign thresholds used in emergency department triage.

> **NOTE** Appendix A, "Frequently Asked Questions and Post-Quiz Materials for Chapters 2–8," includes frequently asked questions and post-test assessment questions for Chapters 2 through 8. These sections can be incorporated into a locally developed ESI training course.

References

American College of Emergency Physicians. (2003). Clinical policy for children younger than 3 years presenting to the emergency department with fever. *Annals of Emergency Medicine*, *43*(4), 530–545. https://doi.org/10.1067/s0196-0644(03)00628-0

Australasian College for Emergency Medicine. (2016). *Guidelines on the implementation of the Australasian Triage Scale in emergency departments*. https://acem.org.au/getmedia/51dc74f7-9ff0-42ce-872a-0437f3db640a/G24_04_Guidelines_on_Implementation_of_ATS_Jul-16.aspx

Baraff, L. J. (2000). Management of fever without source in infants and children. *Annals of Emergency Medicine*, *36*(6), 602–614. https://doi.org/10.1067/mem.2000.110820

Baraff, L. J., Bass, J. W., Fleisher, G. R., Klein, J. O., McCracken, G. H., Powell, K. R., & Schriger, D. L. (1993). Practice guideline for the management of infants and children 0 to 36 months of age with fever without source. *Annals of Emergency Medicine*, *22*(7), 1198–1210. https://doi.org/10.1016/s0196-0644(05)80991-6

Beveridge, R., Clarke, B., Janes, L., Savage, N., Thompson, J, Dodd G, Murray, M., Nijssen Jordan, C., Warren, D, & Vadeboncoeur, A. (1998). *Implementation guidelines for the Canadian Emergency Department Triage and Acuity Scale* (CTAS). http://ctas-phctas.ca/wp-content/uploads/2018/05/ctased16_98.pdf

Cooper, R. J., Schriger, D. L., Flaherty, H. L., Lin, E. J., & Hubbell, K. A. (2002). Effect of vital signs on triage decisions. *Annals of Emergency Medicine*, *39*(3), 223–232. https://doi.org/10.1067/mem.2002.121524

Gilboy, N., Travers, D. A., & Wuerz, R. C. (1999). Re-evaluating triage in the new millennium: A comprehensive look at the need for standardization and quality. *Journal of Emergency Nursing, 25*(6), 468–473. https://doi.org/10.1016/s0099-1767(99)70007-3

Gilboy, N., Tanabe, P., Travers, D. A., Eitel, D. R, & Wuerz, R. C. (2003). *The Emergency Severity Index Implementation handbook: A five-level triage system*. Emergency Nurses Association.

Rangel-Frausto, M. S., Pittet, D. M, Costigan, M., Hwang, T., Davis, C. S., & Wenzel, R. P. (1995). The natural history of the systemic inflammatory response syndrome (SIRS): A prospective study. *JAMA*, *273*(2),117–123. https://doi.org/10.1001/jama.1995.0352026003903

Wuerz, R., Milne, L. W., Eitel, D. R., Travers, D., & Gilboy, N. (2000). Reliability and validity of a new five-level triage instrument. *Academic Emergency Medicine*, *7*(3), 236–242. https://doi.org/10.1111/j.1553-2712.2000.tb01066.x

Wuerz, R., Travers, D., Gilboy, N., Eitel, D. R., Rosenau, A., & Yazhari, R. (2001). Implementation and refinement of the Emergency Severity Index. *Academic Emergency Medicine*, *8*(2), 170–176. https://doi.org/10.1111/j.1553-2712.2001.tb01283.x

6

Use of the ESI for Pediatric Triage

This chapter addresses the use of the Emergency Severity Index (ESI) algorithm for triage of patients less than 18 years of age. The chapter incorporates issues identified during a study conducted by the Pediatric ESI Research Consortium (Travers et al., 2006) and from a review of the pediatric triage literature (Hohenhaus et al., 2008), both of which were funded by the Health Services and Resources Administration. The chapter can help general hospital and pediatric nurses quickly and accurately assess children in the emergency department (ED) triage setting. The chapter is meant to serve as guidance for all hospitals regarding use of the ESI for pediatric triage. It is not intended to serve as a substitute for a course on pediatric triage or pediatric emergency care, nor for local policies regarding triage (e.g., whether or not febrile children are treated with antipyretics at triage if they go to the waiting room).

Background and Research

In the current environment of ED crowding, emerging infectious diseases, and natural disasters, it is important to have a reliable triage system in place that enables rapid and accurate assessment of patients. This is particularly important for the most vulnerable ED populations, which include children. Nationwide, there are an estimated 30 million ED visits per year for patients under 18 years of age, accounting for one-fourth of all ED visits (Middleton & Burt, 2006; McDermott et al., 2018). Children's physiological and psychological responses to stressors are not the same as those of adults, and they are more susceptible to a range of injuries and illnesses, from viruses to dehydration to radiation sickness. Given their often-limited ability to communicate with care providers, children can be more difficult to rapidly and accurately assess than their adult counterparts.

Triage tools such as the ESI algorithm are designed to prioritize ED patients for treatment. The earliest version of the ESI was intended for use only with patients older than age 14 (Wuerz et al., 2000). In 2000, specific pediatric vital sign criteria were added to the ESI version 2; this version is intended for triage of patients of any age (Wuerz et al., 2001). While the ESI has been shown to produce valid, reliable triage of the general ED population, later studies of its utility for pediatric patients indicated room for improvement. Hinrichs and colleagues (2005) found low intra- and inter-rater reliability among nurses using the ESI for infant triage. In another single-site study of ESI version 3, researchers found good agreement for ratings conducted prospectively on patients presenting to triage, but poor to good for ratings based on retrospective chart review (Baumann et al., 2005).

The information in this chapter is based on the results of the multi-center study of pediatric triage and a comprehensive review of the pediatric literature. The Pediatric ESI Research Consortium conducted a large, multi-center study of the ESI for pediatric triage and found that, while the overall reliability of ESI version 4 was good, pediatric cases were more often mistriaged than adult cases (Travers et al., 2006). The study evaluated both reliability and validity of the ESI for children, enrolling 155 nurses and 498 patients in the reliability evaluation and 1,173 patients in the validity evaluation across 7 hospitals in 3 states. The sites included urban, rural, suburban, academic, and community hospitals and two dedicated pediatric EDs. The researchers found that nurses make more accurate ESI ratings for trauma cases than medical cases (Katznelson et al., 2006) and that certain types of pediatric patients are harder to triage, including infants, psychiatric patients, and those with fever, rashes, or respiratory problems (Rosenau et al., 2006).

The Pediatric ESI Research Consortium's comprehensive review of the pediatric literature included 15 emergency courses and pediatric emergency textbooks, and the goal of the review was to identify best practices and best evidence relevant to ED triage of children (Hohenhaus et al., 2008). The review noted both strengths and areas for improvement in the existing literature. Strengths included the use of case scenarios for teaching and the existence of many courses that facilitate education on pediatric assessment during emergencies. Areas for improvement included a lack of evidence-based normal pediatric vital sign parameters; the need for a standardized, interdisciplinary approach to assessment and history taking; and

the need for more pediatric triage-specific case scenarios for educational use.

Pediatric Triage Assessment: What Is Different for Pediatric Patients?

The goal of the triage nurse is to assess an ill child rapidly and accurately in order to assign a triage level to guide timely routing to the appropriate emergency department area for definitive evaluation and management. Triage is not a comprehensive assessment of the pediatric patient. The ESI version 4 requires that the triage nurse follow the same algorithm on all patients, pediatric and adult. While the algorithm is the same regardless of age, the decision process in the pediatric patient must include age dependent differences in development, anatomy, and physiology.

The triage nurse needs a good sense of what constitutes "normal" for children of all ages. This knowledge will make it easier to recognize things that should be concerning (e.g., the 6-month-old who is not interested in his or her surroundings or the 2-week-old who is difficult to arouse to feed). The triage nurse must be comfortable interacting with children across the age spectrum and must be well versed in the anatomic and physiologic issues that may put a child at increased risk, as well as certain age-dependent "red flags" that should not be overlooked. The importance of adequate education in pediatrics prior to undertaking the triage of pediatric patients cannot be overemphasized. The following are key points that the triage nurse should keep in mind when assessing a child:

1. Use a standardized approach to triage assessment of the pediatric patient, such as the six-step approach described in the next section. Observe skin color, respiratory pattern, and general appearance. Infants and children cannot be adequately evaluated through layers of clothing or blankets.
2. Infants must be observed, auscultated, and touched in order to get the required information. Their caregivers are critical to their assessment. Using a warm touch and a soft voice will help with the assessment.
3. Infants over about nine months of age and toddlers often have a significant amount of "stranger anxiety." Approaching them in a nonthreatening manner, speaking quietly, getting down to the child's eye level, and allowing them to have a trusted caregiver with them at all times will make the assessment easier. Allowing the child to remain on the caregiver's lap and enlisting that person's help in things like removing clothing and attaching monitors can help ease the child's fears.
4. Elementary school-age and older children can usually be relied on to present their own chief complaint. Some preschoolers may have the verbal skills necessary to do so, but many do not or are simply too shy or frightened. In these cases, the chief complaint and other pertinent information must be ascertained from the child's caregiver.
5. When assessing school-aged children, speak with them and then include the caregiver. Explain procedures immediately before doing them. Do not negotiate.
6. Do not mistake an adolescent's size for maturity. Physical assessment can proceed as for an adult, remembering that they may be as afraid as a smaller child and have many fears and misconceptions. Pain response may be exaggerated.
7. The signs of severe illness may be subtle and easily overlooked in the neonate and young infant. For example, poor feeding, irritability, or hypothermia in an otherwise well appearing neonate are all reasons to be concerned.
8. Cardiac output in the infant and small child is heart-rate dependent: bradycardia can be as dangerous, if not more dangerous, than tachycardia.
9. Infants, toddlers, and preschoolers have a relatively greater body surface area than adults. This puts them at increased risk for both heat and fluid loss. This is compounded in the neonate, who does not have the fully developed ability to thermoregulate. These patients should not be kept undressed any longer than absolutely necessary and should have coverings replaced after a specific area is examined.
10. Hypotension is a late marker of shock in prepubescent children. A hypotensive child is an ESI level 1, requiring immediate lifesaving intervention.
11. Weights should be obtained on all pediatric patients in triage or treatment area. The actual, not estimated, weight (in kilograms) is important to the safe care of a child. Methods for estimating a child's weight may be used for critically ill/injured children (e.g. length-based tape). Weights should not be guessed by the nurse, parent, or caregivers.
12. A hands-on approach to pediatric assessment should accompany the use of technical equipment. As you obtain a child's vital signs, assess skin color, temperature, and turgor. As you auscultate the child's chest with a stethoscope, note the rate and quality of respirations, as well as chest and abdominal movements.
13. Use appropriately-sized equipment to measure children's vital signs.

A Standardized Approach to Pediatric Triage Assessment

It is helpful to think about pediatric assessment in a standardized manner. A general approach to pediatric triage is suggested:

Step 1. Appearance/work of breathing/circulation – quick assessment

Step 2. Airway/breathing/circulation/disability/exposure-environmental control (ABCDE)

Step 3. Pertinent history

Step 4. Vital signs

Step 5. Fever?

Step 6. Pain?

Step 1. Appearance, Work of Breathing, Circulation – Quick Assessment

Most triage nurses are comfortable with an "Airway, Breathing, Circulation, and Disability" (ABCD) assessment approach to help determine whether a child is "sick" or "not sick." In each of the standardized national pediatric emergency education courses, the ABCD approach is preceded by the Pediatric Assessment Triangle (PAT) (American Academy of Pediatrics [AAP], 2016). The PAT uses visual and auditory cues and is performed at the first contact with a pediatric patient. It can be completed in less than 60 seconds. The PAT is an assessment tool, not a diagnostic tool and assists the nurse with making quick life support decisions using appearance, work of breathing, and circulation to skin. A child's appearance can be assessed from across the room and includes tone, level of interactivity, consolability, look/gaze, and speech/cry. A child's work of breathing is characterized by the nature of airway sounds, positioning, retractions, and flaring. Circulation to skin is assessed by observing for pallor, mottling, or cyanosis. By combining the three parameters of the PAT, the nurse can get a quick idea of the physiological stability of a child and, in conjunction with the chief complaint, make decisions regarding the need for life support. Some patients may need to be taken immediately to the treatment area to address abnormalities found in the quick assessment. For more stable patients, the nurse will proceed to the next step in the assessment, ABCDE.

Step 2. Airway, Breathing, Circulation, Disability, Exposure/Environmental Control (ABCDE)

Following the urgency decision made with the PAT, a primary assessment using the ABCDE process can then be followed (Emergency Nurses Association, 2020). This assessment must be done in order and includes assessing for airway patency, respiratory rate and quality, heart rate, skin temperature and capillary refill time, blood pressure (where clinically appropriate, such as a child with cardiac or renal disease), and an assessment for disability or neurological status. A child's neuro logical status can be obtained by assessing appearance, level of consciousness, and pupillary reaction. Exposure involves undressing the patient to assess for injury or illness and addressing any immediate environmental needs such as treating fever or providing warmth. Exposure and environmental control may happen at triage or in the treatment area, depending on the patient's condition and factors such as treatment room availability. Any serious finding in the ABCDE assessment indicates a need for immediate treatment and may require deferral of the next steps in the assessment.

Table 6-1. SAMPLE History Mnemonic

Initials	Terms
S	Signs/symptoms
A	Allergies
M	Medications
P	Past medical problems
L	Last food or liquid
E	Events leading to injury/illness

Brecher, D. (Ed.) (2020). *Emergency Nursing Pediatric Course provider manual.* Emergency Nurses Association.

Step 3. Pertinent History

Following performance of the initial assessment of a child at triage, a standardized history should be obtained. Table 6-1 presents one system for doing so. The history may be deferred to the primary nurse if the triage nurse identifies the need for any lifesaving interventions or a high-risk situation.

Which method is chosen is not nearly as important as using a consistent method to avoid missing important information.

Step 4. Vital Signs

Hohenhaus et al. pointed out in 2008 that there was a lack of rigorous studies to support the various vital sign parameters that are included in the major pediatric emergency texts and courses such as Pediatric Advanced Life Support (AHA, 2016), Advanced Pediatric Life Support (AAP, 2015), Pediatric Education for Prehospital Professionals (AAP, 2016), and Emergency Nursing Pediatric Course (Brecher, 2020). Subsequent studies (e.g., Van Kuiken & Huth, 2013; Sepanski et al., 2018) as yet have not provided any more definitive conclusions. The major courses and texts appear to represent consensus recommendations for normal vital sign parameters and include various age groupings and parameters. Version 4 of the ESI includes parameters drawn from the literature (Wuerz et al., 2000).

The following are recommendations regarding the use of blood pressure and oxygen saturation measurements for ESI decisions (Keddington, 1998):

- Blood pressure measurement is not a critical factor in assigning acuity, and its measurement should be left to the judgment of the triage nurse.

- Oxygen saturation should be measured in infants and children with respiratory complaints or symptoms of respiratory distress.

Pulse oximetry values may be interpreted differently at high altitude; EDs in such settings may need to develop local protocols to address this (Gamponia et al., 1998).

It is essential that equipment used in pediatric physical assessment is the correct size. Some research has shown that nurses often use adult-sized equipment for children, which may result in errors in vital signs measurements (Hohenhaus, 2006).

Step 5. Fever?

Unlike in adult patients, decision-making regarding the febrile child must consider both the clinical picture and the child's age. Note D on the ESI version 4 addresses pediatric fever considerations. These considerations are based on published guidelines from emergency physicians (American College of Emergency Physicians [ACEP], 2003, 2016). However, since those recommendations were published, the heptavalent conjugate pneumococcal vaccine has become a routine part of the infant immunization series. With this in mind, many physicians are changing their practice and not routinely ordering blood work (including cultures) on febrile children who do not appear toxic and have completed this immunization series. Thus, the current Pediatric Fever Considerations in the ESI version 4 reflect the fact that the fever criteria continue to evolve. The guidelines for children with fever (38°C or 100.4°F or greater) who are in the first 28 days of life are clear – these patients must be rated ESI level 2 as they may have serious infections. The ESI guidelines recommend that triage nurses consider assigning ESI level 2 for infants 1–3 months of age with fever, while taking into consideration practices in their institution. Nurses may have to adjust their fever considerations according to those practices for 1- to 3-month-olds.

Other considerations include exposure to known significantly sick contacts (e.g., diagnosed with influenza, meningococcal meningitis) and immunization status. An immunization history should be ascertained at the time of triage. It may be helpful to post a copy of the Recommended Immunization Schedule for Persons Aged 0–6 Years (Robinson et al., 2019) at triage. Febrile children over the age of 2 who have not completed their primary immunization series should be considered higher risk than their immunized counterparts with similar clinical presentations. The triage nurse should consider making these patients at least an ESI level 3 if there is no obvious source of fever.

Step 6. Pain?

Section B on the ESI version 4 defines severe pain/distress as determined either by clinical observation or a patient rating of ≥ 7 on a 0–10 pain scale. Pain assessment for children should be conducted using a validated pediatric pain scale. Pediatric patients who meet the ≥ 7 criterion should be considered for triage as an ESI level 2. The triage nurse is not required to assign these patients an ESI level 2 rating and should use sound clinical judgment in making the final decision. For example, a child who reports his pain as an 8/10 but is awake, alert, smiling, and in no apparent distress may not warrant triage as a level 2. Neither does the young child with a minor injury simply because they are screaming loudly. There are several validated pediatric pain scores. For example, the FLACC (Face, Legs, Activity, Consolability) scale for infants and nonverbal children and the FACES scale for those who are not able to understand the 0–10 scale are both validated, easy-to-use scoring systems (Bieri et al., 1990; Keck et al., 1996; Luffy & Grove, 2003; Merkel et al., 1997).

Each institution should decide for itself which pain scale(s) to use for pediatric patients. What is important is that a validated pediatric pain scale be available and used correctly and consistently by the triage nurse. This may require additional education in pain scales that is outside the scope of this handbook but should be part of an institution's in-service program.

Assessment of Rashes

Analysis of nurses' ratings of pediatric patients with the ESI has found that triage nurses both under- and overtriage rash patients (Travers et al., 2009). During this study, nurses gave feedback that it is sometimes hard to differentiate high-risk rashes (e.g., meningococcemia) from low-risk rashes (e.g., contact dermatitis). When triaging the patient with a rash, the nurse should obtain a thorough history and complete set of vital signs. Other associated symptoms should be ascertained, and the overall appearance of the child should be considered. The child should be undressed if necessary to adequately visualize the rash. Rashes that should raise an immediate "red flag" and warrant an ESI level 2 include vesicular rashes in the neonate and petechial and purpuric rashes in children of any age. If a child has a petechial rash with altered mental status, they should be rated as ESI level 1; they are at risk of meningococcemia and may be in shock. They will likely need significant intravenous fluid resuscitation and antibiotics.

Infant Triage

For the purposes of ESI triage, an "infant" is defined as any child who has not yet reached his or her first birthday. This definition is consistent with the ACEP definitions, as well as the PALS guidelines regarding equipment size and cardiopulmonary resuscitation standards (ACEP, 2003, 2016; AHA, 2016). Of all the patients who present to the emergency department, infants may be the most difficult for the triage nurse to evaluate. These patients' lack of verbal skills and often subtle signs of serious illness can make an accurate assessment difficult. Parental con-

Table 6-2. Examples of ESI Level-1 Conditions
Respiratory arrest
Cardiopulmonary arrest
Major head trauma with hypoventilation
Active seizures
Unresponsiveness
Petechial rash in a patient with altered mental status (regardless of vital signs)
Respiratory failure<br>• Hypoventilation<br>• Cyanosis<br>• Decreased muscle tone<br>• Decreased mental status<br>• Bradycardia (late finding, concerning for impending cardiopulmonary arrest)
Shock/sepsis with signs of hypoperfusion<br>• Tachycardia<br>• Tachypnea<br>• Alteration in pulses: diminished or bounding<br>• Alteration in capillary refill time > 3 to 4 seconds<br>• Alteration in skin appearance: cool/mottled or flushed appearance<br>• Widened pulse pressure<br>• Hypotension (often a late finding in the prepubescent patient)
Anaphylactic reaction (onset in minutes to hours)<br>• Respiratory compromise (dyspnea, wheeze, stridor, hypoxemia)<br>• Reduced systolic blood pressure<br>• Hypoperfusion (e.g., syncope, incontinence, hypotonia)<br>• Skin and/or mucosal involvement (hives, itch-flush, swollen lips, tongue, or uvula)<br>• Persistent gastrointestinal symptoms

cerns about signs and symptoms, even those not witnessed by the triage nurse, must be taken seriously. Whether the report is of a physical sign (e.g., fever) or an abnormal behavior (e.g., fussy/irritable), parents are the best judges of their infant, and if they are concerned, they often have a good reason to be.

Of all the patients who present to the emergency department, infants may be the most difficult for the triage nurse to evaluate. These patients' lack of verbal skills and often subtle signs of serious illness can make an accurate assessment difficult. Parental concerns about signs and symptoms, even those not witnessed by the triage nurse, must be taken seriously. Whether the report is of a physical sign (e.g., fever) or an abnormal behavior (e.g., fussy/irritable), parents are the best judges of their infant, and if they are concerned, they often have a good reason to be.

When assessing an infant, the triage nurse must pay close attention to the history offered by the parents as this may be the only real clue to the problem. The infant's state should be assessed prior to handling. Vital signs must be assessed using appropriate-sized equipment and need to be part of the triage process of any infant who does not immediately fall into the ESI levels 1 or 2. Vital sign abnormalities may be the only outward signs of a serious illness. Infants must be unwrapped and undressed for a hands-on assessment of perfusion and respiratory effort, remembering that they can rapidly lose body heat in a cool environment and should be rewrapped as soon as possible.

Fever guidelines for infants are discussed above. Specific practices for the evaluation of febrile older infants may differ from institution to institution. However, it is universally accepted that neonates (< 28 days of age) with a rectal temperature of 38°C (100.4°F) or greater are considered high risk for a serious bacterial infection and should be triaged accordingly (at least at an ESI level 2). Infants with rectal temperatures of 38°C (100.4°F) or higher are likely to need a full sepsis workup (including blood, urine, and cerebrospinal fluid cultures) and parenteral antibiotic administration.

Assigning ESI Levels for Pediatric Patients

This section focuses on assigning ESI levels to pediatric patients.

ESI Level 1

ESI level-1 patients are the highest acuity patients that present to the ED. Because ESI level-1 patients are clinically unstable, decisions about resources needed during the ED stay are not considered. These patients require a physician and a nurse at the bedside to provide lifesaving critical care interventions. They

Table 6-3. Examples of ESI Level-2 Conditions
Syncope
Immunocompromised patients with fever
Hemophilia patients with possible acute bleeds<br>• Joint pain or swelling<br>• History of fall or injury<br>• Vital signs and/or mental status outside of baseline
Febrile infant < 28 days of age with T ≥ 38°C (100.4°F) rectal
Hypothermic infants < 90 days of age with T < 36.5°C (97.7°F) rectal
Suicidality
Possible meningitis (headache/stiff neck/fever/lethargy/irritability)
Seizures – prolonged postictal period (altered level of consciousness)
Moderate to severe croup
Lower airway obstruction (moderate to severe)<br>• Bronchiolitis<br>• Reactive airway disease (asthma)<br>• Respiratory distress<br>• Tachypnea<br>• Tachycardia<br>• Increased effort (nasal flaring, retractions)<br>• Abnormal sounds (grunting)<br>• Altered mental status

cannot wait, even a brief time, for initiation of treatment. Research has found that the ESI level-1 rating is under-utilized by nurses triaging critically ill children, except for those children who are intubated or in cardiac arrest (Travers et al., 2009). In response to findings from an all-age study, the ESI was modified in version 4 to classify any patient in need of immediate, life-saving interventions as ESI level 1; formerly, these patients were often thought of as "sick level 2s" (Tanabe et al., 2005). Table 6-2 provides examples of ESI level-1 conditions. This is not an exhaustive list.

ESI Level 2

As with assigning an ESI level-1 acuity, assigning an ESI level-2 acuity is based on the clinical condition of the patient, and it is not necessary to consider resource utilization in the decision. ESI level-2 decisions are based on the history and assessment findings indicative of sentinel symptom complexes that signal a high-risk, or potentially high-risk, situation. Table 6-3 provides examples of patient problems that warrant ESI level-2 ratings. This is not an exhaustive list.

Resource Considerations When Using the ESI for Pediatrics

As with use of the ESI for adult patients, its use for children includes resource prediction as a way of differentiating the three lower acuity levels, ESI levels 3 4, and 5. It is sometimes a challenge to predict resource needs for pediatric patients. The triage nurse may find it especially challenging to differentiate pediatric patients predicted to need two resources (ESI level 3), versus one resource (ESI level 4) or no resources (ESI level 5).

One reason for this is that some conditions require different numbers of resources in children than in adults. Research has shown that ESI level 5 is underutilized for pediatric patients (Travers et al., 2009). These issues will be explored in this section.

Pediatric patients may occasionally warrant a different ESI level than an adult for a comparable problem. For example, adults with lacerations that necessitate suturing are typically classified as ESI level 4. However, some pediatric patients may require sedation for a laceration repair, particularly if they are below school age or appear to be especially agitated or uncooperative. Sedation includes the establishment of intravenous access, administration of intravenous medications, and close monitoring; thus, all sedation patients are classified as at least ESI level 3 based on their need for more than one resource. Table 6-4 lists examples of children who are candidates for sedation.

Table 6-4. Examples of Situations That May Warrant Sedation in Pediatric Patients
Fracture/dislocation repair in ED
Complicated lacerations, such as:<br>• Complex facial/intraoral lacerations<br>• Lacerations across the vermillion border<br>• Lacerations requiring a multilayered closure<br>• Extremely dirty or contaminated wounds
Computed tomography/magnetic resonance imaging procedures or image-guided procedures (e.g., joint aspirations under bedside ultrasound, fluoroscopy)
Lumbar punctures (except in infants)
Chest tube insertions

It is important to remember that the ESI is not a nursing workload measure. Rather, resources are used in the ESI as a proxy for acuity. A child with a small abrasion (ESI level 5) who gets the wound cleansed and a tetanus shot is less acute than a patient with a sprained ankle (ESI level 4) who gets a radiograph, ace wrap, and crutch-walking instruction. In turn, such a patient is less acute than a child with a complex laceration (ESI level 3) who gets suturing and sedation. While the tetanus injection, ace wrap and crutch-walking instruction all require nursing time, they are not considered ESI resources. The purpose of the ESI resource assessment is to sort patients into 5 meaningful acuity categories, not to estimate the nursing workload intensity. EDs are encouraged to use appropriate workload measures to capture nursing resource needs. Table 6-5 lists conditions of patients who need no resources and can be classified as ESI level 5.

Table 6-5. Examples of ESI Level 5
Medication refills
Ear pain in healthy school-age children
Contusions and abrasions
Upper respiratory infection symptoms with normal vital signs
A 2-year-old with runny nose, mild cough, and temp of 38°C (100.4°F), active and drinking during triage
Poison ivy on extremities

Special Populations

The special populations discussed in this section include trauma and psychiatric pediatric patients, as well as children with comorbidities.

Trauma

Trauma patients can be challenging to triage, especially if they have suffered internal injuries without visible external signs of injury. Pediatric trauma patients may be difficult to assess due to compensatory mechanisms that produce vital signs with the appearance of stability. The nurse must be proactive when providing care to the pediatric trauma patient to prevent deterioration and rapid decompensation. Children who suffer traumatic injuries must be assessed and assigned a triage level based on the mechanism of injury and presenting signs and symptoms, as opposed to basing the ESI rating on the practices of individual triage nurses or mode of arrival to the ED. For example, children should not be assigned an ESI level based on the fact that their arrival was by ambulance or that back boards and cervical collars were used. Any patient with a high-risk mechanism of injury should be classified as ESI level 2, unless their condition requires immediate lifesaving interventions that warrant classification as ESI level 1. Vital signs and estimation of resource needs are not needed for ESI level-1 or level-2 determinations. Examples of pediatric trauma patients and ESI ratings are provided in Table 6-6.

Psychiatric

Psychiatric emergencies among children present a unique challenge for the triage nurse, who will be required to make a complex clinical decision as to the degree of danger the patient may pose to themselves or others. Patients at high risk may exhibit a variety of symptoms including violent or combative behavior, paranoia, hallucinations, delusions, suicidal/homicidal ideation, acute psychosis, anxiety, and agitation and should be rated ESI level 2. The Mental Health Triage Scale can be used in the assessment of the pediatric psychiatric patient (Smart, Pollard, & Walpole, 1999). Any child presenting as confused, disorganized, disoriented, delusional, or hallucinating should be rated as an ESI level 2. These altered mental states may be attributed to the patient's mental health or medical or neurological complications (Brecher, 2020). The amount of distress a child appears to be in, or has reportedly been in, can also classify them as an ESI level 2. The triage nurse should be alert for any behaviors that may indicate the patient is a high risk and needs treatment immediately. A patient's distress should not be limited to physical symptoms but may include situational triggers as well. Therefore, it is important to be aware of the circumstances underlying the current psychological event. In addition to establishing the reason for the exhibited behavior, it is important to capture the type, severity, frequency, and focus (is the behavior directed toward something or someone) of the behavior. In some cases, it may be beneficial to interview older children and adolescents alone. They may be more likely to offer information on sensitive subjects such as risky behaviors, abusive relationships, and drug or alcohol use without the presence of their parents.

Resources will determine whether the patient will fall into ESI level 3, level 4, or level 5. Resources will be somewhat different for the pediatric mental health patient than for the pediatric medical patient and are likely to include things such as psychiatric and social work consults. Table 6-7 provides examples of pediatric psychiatric patients.

Children with Comorbid Conditions

Research has found that children with comorbid conditions are both over-triaged and undertriaged (Travers et al., 2009). Patients with chronic conditions (e.g., spina bifida, seizures, metabolic syndromes, short gut) may require more extensive evaluation and workup than otherwise healthy children with similar complaints. At the same time, children should not be automatically triaged at a higher level due to a comorbid condition. A good history and input from the child's caregiver can help greatly in this determination. For example, the child with a known seizure disorder who presents with breakthrough seizures needs to be triaged at a higher level than the same child who presents for a medication refill. The febrile 10-year-old with a ventriculoperitoneal shunt is going to need more extensive evaluation than the otherwise healthy and non-toxic appearing 10-year-old with an isolated fever. However, a child with a sprained ankle likely does not need a higher acuity level simply because the child has a history of congenital heart disease.

Pediatric Patient Case Studies

In addition to this pediatric chapter of the ESI Handbook, several sets of pediatric case studies are available to support pediatric-specific ESI education in locally developed ESI educational programs. Pediatric case studies are included in Chapter 9, "Practice Cases" and Chapter 10, "Competency Cases" of this handbook. There are also additional cases available in Gilboy et al. (2005).

Summary

Assessing the pediatric patient can be a daunting task for both the novice and the experienced triage nurse. Remembering some key developmental differences between pediatric and adult

Table 6-6. Examples of Pediatric Trauma and ESI Levels

Patient Presentation	Resources	ESI	Rationale
A 7-year-old male who was hit by a car arrives by ambulance. The child is somnolent and appears pale, with nonlabored respirations.	Lifesaving intervention, no need to assess for number of resources	ESI level 1	Life threatening injury
A 14-year-old female is brought in by ambulance after diving into the pool and hitting her head. She is awake, alert, and moving all extremities. She is currently on a back board with cervical collar in place. VS: BP 118/72 mm Hg, HR 76 beats/minute, RR 14 breaths/minute.	High risk injury, no need to assess for number of resources	ESI level 2	High risk injury due to the mechanism
A 14-year-old male who was tackled while playing football arrives by ambulance. He has an obvious deformity to his right lower leg. He has +2 pedal pulses and his toes are warm and dry. He is able to wiggle his toes. No head/neck injuries reported. VS: BP 118/78 mm Hg, HR 88 beats/minute, RR 18 breaths/minute, T 36.7°C (98.2°F), pain 6/10.	More than one resource	ESI level 3	Fracture will need reduction. He will need radiographs, labs, intravenous antibiotics, and pain medication.
A 12-year-old female is brought into the ED by her mother. States she cut her thumb while washing dishes. She has a 2 cm superficial laceration to her right thumb. VS: BP 110/70, HR 72, RR 14, T 36.6°C (98°F.)	One resource	ESI level 4	Will require suturing

Table 6-7. Examples of Pediatric Psychiatric Patients and ESI Levels

Patient Presentation	Resources	ESI	Rationale
A 17-year-old male, history of suicidality, found unresponsive by parents. There are several bottles of liquor and a number of unidentified empty pill bottles next to bed.	Lifesaving intervention. No need to assess for resources.	ESI level 1	Life threatening situation – unresponsive
A 16-year-old male brought in by parents who report patient was out of control, screaming obscenities, and threatening to kill the family. He is cooperative in triage and answers your questions calmly.	High-risk situation. No need to assess for number of resources.	ESI level 2	High risk situation – danger to self and others
A 15-year-old female presents to the ED with her boyfriend claiming, "I think I'm pregnant. When I told my mom, she threw me out of the house and told me never to come back. I have no place to live, and now I might have a baby." VS: BP 126/85, HR 100, RR 16, T 37.1°C (98.7°F).	More than one resource	ESI level 3	Will require labs and possibly more than one specialty consult
A 10-year-old female presents to the ED with mother who states that she received a call from her teacher because the child has been disrupting the class with sudden outbursts. Mom has never witnessed this behavior, but she does state that she becomes very defiant when she does not get her own way. Currently, the child is laughing and playing with her little sister. VS: BP 98/72 mm Hg, HR 82 beats/minute, RR 22 breaths/minute, T 36.7°C (98.2°F).	One resource	ESI level 4	Will require a consult
A 13-year-old male walks into the ED with his mother on a Friday night. Mom states, "I didn't realize he was out of his medications for his ADHD, and I don't want him to miss a day." The patient is cooperative and pleasant. VS: BP 108/72 mm Hg, HR 78 beats/minute, RR 14 breaths/minute, T 37°C (98.6°F).	No resources	ESI level 5	Will require a prescription filled

patients can help make the process significantly less stressful for ill or injured children and their caregivers. Applying the ESI algorithm consistently on patients of all ages, while keeping in mind key anatomical and physiological differences in the pediatric population, can simplify the process for the triage nurse.

In order to triage pediatric patients most effectively, the triage nurse must be experienced in caring for the youngest patients.

This chapter highlights important factors to keep in mind when triaging the pediatric patient, including the value of using a standardized approach to assessment such as PAT, keeping special populations in mind when determining which patients are high risk, and the importance of communication with the accompanying caregiver.

This chapter was made possible by a grant (# H34MC04371) through the Health Resources and Services Administration, Maternal Child and Health Bureau, Emergency Medical Services for Children (EMSC) Program. The grant supported the work on this chapter by the Pediatric ESI Research Consortium:

- *University of North Carolina at Chapel Hill (Chapel Hill, NC): Anna Waller (principal investigator) Debbie Travers, Jessica Katznelson*
- *Wellspan Health System (York, PA): David Eitel, Suanne McNiff*
- *Primary Children's Medical Center (Salt Lake City, UT): Nancy Mecham*
- *Lehigh Valley Health Network (Allentown, PA): Alexander Rosenau, Valerie Rupp*
- *WakeMed Health and Hospitals (Raleigh, NC): Douglas Trocinski.*
- *Hohenhaus and Associates, Inc (Wellsboro, PA): Susan McDaniel Hohenhaus*

NOTE Appendix A, "Frequently Asked Questions and Post-Quiz Materials for Chapters 2–8," includes frequently asked questions and post-test assessment questions for Chapters 2 through 8. These sections can be incorporated into a locally developed ESI training course.

References

American Academy of Pediatrics. (2011). *APLS: The Pediatric Emergency Medicine Resource* (5th ed.). Jones & Bartlett Learning.

American Academy of Pediatrics. (2016). *Pediatric Education for Prehospital Professionals* (3rd ed.). Jones & Bartlett Learning.

American College of Emergency Physicians Clinical Policies Committee, Subcommittee on Pediatric Fever. (2003). Clinical policy for children younger than three years presenting to the emergency department with fever. *Annals of Emergency Medicine, 42*(4), 530–545. https://doi.org/10.1067/S0196-0644(03)00628-0

American College of Emergency Physicians Clinical Policies Subcommittee on Pediatric Fever. (2016). Clinical policy for well-appearing infants and children younger than 2 years of age presenting to the emergency department with fever. *Annals of Emergency Medicine*, *67*(5), 625–639. https://doi.org/10.1016/j.annemergmed.2016.01.042

American Heart Association (2016). *Pediatric Advanced Life Support Provider Manual.* Author.

Baumann, M. R., & Strout, T. D. (2005). Evaluation of the Emergency Severity Index (Version 3) triage algorithm in pediatric patients. *Academic Emergency Medicine*, *12*(3), 219–224. https://doi.org/10.1197/j.aem.2004.09.023

Bieri, D., Reeve, R. A., Champion, G. D., Addicoat, L., & Ziegler, J. B. (1990). The Faces Pain Scale for the self-assessment of the severity of pain experienced by children: Development, initial validation, and preliminary investigation for the ratio scale properties. *Pain*, *41*(2), 139–150. https://doi.org/10.1016/0304-3959(90)90018-9

Brecher, D. (Ed.) (2020). *Emergency Nursing Pediatric Course provider manual.* Emergency Nurses Association.

Gamponia, M. J. , Babaali, H., & Gilman, R. H. (1998). Reference values for pulse oximetry at high altitude. *Archive of Disease in Childhood*, *78*(5), 461–465. https://doi.org/10.1136/adc.78.5.461

Gilboy, N., Tanabe, P., & Travers, D. (2005). The Emergency Severity Index Version 4: Changes to ESI level 1 and pediatric fever criteria. *Journal of Emergency Nursing*, *31*(4), 357–362. https://doi.org/10.1016/j.jen.2005.05.011

Hohenhaus, S. M. (2006). Someone watching over me: Observations in pediatric triage. *Journal of Emergency Nursing*, *32*(5), 398–403. https://doi.org/10.1016/j.jen.2006.07.002

Hohenhaus, S. M., Travers, D., & Mecham, N. (2008). Pediatric triage: A review of emergency education literature. *Journal of Emergency Nursing*, *34*(4), 308–313. https://doi.org/10.1016/j.jen.2007.06.022

Katznelson, J., Hohenhaus, S., Travers, D., Agans, R., Trcinski, D., & Waller, A. (2006). Creation of a validated set of pediatric case scenarios for the Emergency Severity Index triage system [Abstract]. *Academic Emergency Medicine*, *13*(S5), S169. https://doi.org/10.1111/j.1553-2712.2006.tb02227.x

Keck, J., Gerkensmeyer, J., Joyce, B., & Schade, J. (1996). Reliability and validity of the FACES and Word Descriptor scales to measure pain in verbal children. *Journal of Pediatric Nursing*, *11*(6), 368–374. https://doi.org/10.1016/s0882-5963(96)80081-9

Keddington, R. (1998). A triage vital sign policy for a children's hospital emergency department. *Journal of Emergency Nursing*, *24*(2), 189–192. https://doi.org/10.1016/S0099-1767(98)90030-7

Luffy, R., & Grove, S. K. (2003). Examining the validity, reliability, and preference of three pediatric pain measurement tools in African-American children. *Pediatric Nursing*, *29*(1), 54–59.

McDermott, K. W., Stocks, C., Freeman, W. J. (2018). *Overview of pediatric emergency department visits, 2015* (Statistical brief no. 242). Healthcare Cost and Utilization Project, Agency for Healthcare Research and Quality. https://www.hcup-us.ahrq.gov/reports/statbriefs/sb242-Pediatric-ED-Visits-2015.pdf

Merkel, S.I., Voepel-Lewis, T., Shayevitz, J. R., & Malviya, S. (1997). The FLACC: A behavioral scale for scoring postoperative pain in young children. *Pediatric Nursing*, *23*(3), 293–297.

Middleton, K. R., & Burt, C. W. (2006). Availability of pediatric services and equipment in emergency departments: United States, 2002–03. *Advance Data.* 367, 1–16.

Robinson, C. L., Bernstein, H., Romero, J. R., & Szilagyi, P. (2019). Advisory Committee on Immunization Practices recommended immunization schedule for children and adolescents aged 18 years or younger — United States, 2019. *MMWR Morbidity and Mortality Weekly Report*, *68*(5), 112–114. https://doi.org/10.15585/mmwr.mm6805a4

Rosenau, A., Waller, A., Trcinski, D., Travers, D., Mecham, N., Katznelson, J. Hosenhaus, S., Eubanks, T., Rupp, V., & Eitel, D. (2006). Is the Emergency Severity Index reliable for pediatric triage? [Abstract]. *Annals of Emergency Medicine*, *48*(4S), 62–63. https://doi.org/10.1016/j.annemergmed.2006.07.657

Sepanski, R. J., Godambe, S. A., & Zaritsky, A. L. (2018). Pediatric vital sign distribution derived from a multi-centered emergency department database. *Frontiers in Pediatrics*, *6*, 66. https://doi.org/10.3389/fped.2018.00066

Smart, D., Pollard, C., & Walpole, B. (1999). Mental health triage in emergency medicine. The *Australian and New Zealand Journal of Psychiatry*, *33*(1), 57-–69. https://doi.org/10.1046/j.1440-1614.1999.00515.x

Tanabe, P., Travers, D., Gilboy. N., Rosenau, A., Sierzega, G., Rupp, V., Martinovich, Z., Adams, J. G. (2005). Refining Emergency Severity Index triage criteria. *Academic Emergency Medicine*, *12*(6), 497–501. https://doi.org/10.1197/j.aem.2004.12.015

Travers, D., Agans, R., Eitel, D., Mecham, N., Rosenau, A., Tanabe, P., Trocinski, D., & Waller, A. (2006). Reliability evaluation of the Emergency Severity Index Version 4 [Abstract]. *Academic Emergency Medicine*, *13*(S5), S126. https://doi.org/10.1111/j.1553-2712.2006.tb02227.x

Travers, D. A., Waller, A. E., Katznelson, J., & Agan, R. (2009). Reliability and validity of the Emergency Severity Index for pediatric triage. *Academic Emergency Medicine*, *16*(9), 843–849. https://doi.org/10.1111/j.1553-2712.2009.00494.x

Van Kuiken, D., & Huth, M. M. (2013). What is 'normal?' Evaluating vital signs. *Pediatric Nursing*, *39*(5), 216–224.

Wuerz, R., Milne, L. W., Eitel, D. R., Travers, D., & Gilboy, N. (2000). Reliability and validity of a new five-level triage instrument. *Academic Emergency Medicine*, *7*(3), 236–242. https://doi.org/10.1111/j.1553-2712.2000.tb01066.x

Wuerz, R., Travers, D., Gilboy, N., Eitel, D. R., Rosenau, A., & Yazhari, R. (2001). Implementation and refinement of the Emergency Severity Index. *Academic Emergency Medicine*, *8*(2), 170–176. https://doi.org/10.1111/j.1553-2712.2001.tb01283.x

7

Implementation of ESI Triage

Up to this point, an in-depth discussion has been provided of the Emergency Severity Index (ESI) algorithm and how to apply it to individual patients. To help ensure successful adoption of the ESI in an emergency department (ED), a well-thought-out implementation plan is critical. Change has become constant, pervasive, and persistent in health care. It is important to keep in mind that implementation of any new system or process takes time, careful planning, and a group of professionals dedicated to a successful transition.

This chapter presents background information on the change process in health care organizations and a step-by-step guide for successful implementation of the ESI. The implementation strategies successfully used by members of the ESI research team and others are presented.

Decision-Making and Planning

The decision to switch from another triage acuity rating system to ESI may be based on multiple reasons. One reason may be the American College of Emergency Physicians (ACEP) and the Emergency Nurses Association (ENA) joint policy statement (ACEP & ENA, 2017) on a standardized triage scale and acuity categorization that supports the adoption of a reliable, valid five-level triage scale such as ESI. In many institutions, a particular event may be the impetus for the change, such as a mistriage or a sentinel event due to prolonged patient waiting time. The clinical or administrative staff may express concerns about patient safety. The nursing staff may find that they are continuously re-triaging patients. In crowded EDs with many urgent patients waiting to be seen, nurses are forced to constantly reprioritize these patients for the scarce ED beds. The implementation of the ESI may be part of a larger plan, but before transitioning to a new triage acuity rating system, the implementation team needs to consider all aspects of the "door to doctor" process.

Revising the system requires understanding of the planned change process. Planned change results from a well-thought-out and conscious effort to improve something. Kurt Lewin's theory of planned change is a frequently used approach in health care organizations (Nelson, 2002). Lewin identified three phases of change:

- Unfreezing
- Movement
- Refreezing

These steps parallel the steps in the nursing process that triage nurses follow. The first step in implementing any change is to recognize that a problem exists and that there is a clear need for change. This **unfreezing phase** is often compared to assessment, the first step of the nursing process. During this phase, data are gathered and the problem or problems are identified. Both informal and formal discussions may occur concerning the problem and the need for change. In the ED, this may occur at nursing and physician meetings or during informal discussions in the clinical area. In many cases, one individual, typically a nurse or physician in a leadership role, drives the push for change. This "champion" should take every opportunity to discuss the problem and explain why a change needs to occur. Hospital and department leadership have to create a sense of urgency regarding the change. Data that show staff that the system they are using is not working can help engender support for changing triage systems. Such data may include mistriages per week, numbers of patients who leave without being seen, and delays in physician evaluation of high-risk patients.

As in the nursing process, during the **movement phase**, those charged with carrying out the change (the change agent or agents) identify, plan, and implement suitable strategies. The last phase, the **refreezing phase**, is similar to the evaluation and reassessment phase of the nursing process. At this stage, the champions of the new system need to ensure that the change has been successfully integrated into the day-to-day operations of the ED.

Once the decision is made to change to the ESI, a multidisciplinary implementation team needs to be identified. The implementation team becomes the change agent. The implementation team leader is a key player in the successful implementation of the ESI and needs to have the respect of the

department as well as strong skills in leadership, communication, problem solving, and decision-making.

Selection of the team members is paramount to the timely success of lasting change. Membership must include management, physicians with a collaborative style, nursing staff with triage expertise, the clinical educator or clinical nurse specialist, and the triage committee if the department has one. Staff in other disciplines, such as registration and information systems, who will be affected by the change, may also be asked to join the team. These members may be invited to attend meetings on an as-needed basis. The group should consider asking one or more of the informal nursing leaders to be staff nurse team members. This will facilitate the informal leaders' buy-in of the change, which will be helpful if staff begins to raise concerns about the change to ESI.

It is important for the implementation team to meet regularly. Department leadership needs to arrange for staff to be available during meeting time. It is well established that without adequate planning, implementation will fail. Implementation is never a single action but involves a well-designed comprehensive plan, a stepwise process, and a variety of strategies and interventions (Grol & Grinshaw, 1999).

The implementation team must decide what needs to be done, who will do it, and what strategies will be used. They must also develop a timeline. Other teams have found flow-charting or using a computer project application helpful. A flow chart identifies the critical tasks that need to occur and links them with completion target dates. The team members can regularly refer to the flow chart to see if they are meeting their target dates.

At Brigham and Women's Hospital in Boston, the team brainstormed to identify who and what would be affected by the change to ESI. The list generated by this process included the following:

- Information systems
- The patient tracking system
- The physician record
- The nursing record
- Triage policies and procedures
- Triage orientation

Visiting other EDs that have already implemented ESI can be very informative. Start by contacting managers, educators, or clinical nurse specialists at area EDs to identify EDs using ESI. Visiting a department that has been using ESI for at least 6 months should be most beneficial. The leadership team may share valuable information about their own implementation experience, including issues they encountered and strategies that worked well. It is important to plan these visits to make sure that all of the group's open issues are addressed. Prior to the visit, make a list of questions and information the team needs. Be sure to request copies of policies and documentation forms. If team members have questions that cannot be answered by the publications, this handbook, or others who have implemented ESI, they can contact ENA at education@ena.com.

Changing to ESI takes several months of planning, and timing is important. Once all the tasks associated with the change are identified and timeframes established, the group can choose a realistic implementation date. The team must consider what is happening in the hospital and in the ED and identify a time when the unit is able to support the change and the educational activities. The acuity system cannot be changed gradually. A definite start date and time must be set and shared with all staff affected by the change.

Policies and Procedures

All policies related to triage must be reviewed in light of the change to ESI. Individual hospitals must decide how the ESI will be incorporated into their ED's existing triage policies and procedures. Many policies may need to be rewritten. Examples of policies and procedures that need to be addressed include the following:

- Where are different types of patients seen within the ED? This varies by hospital, depending on the ED structure and patient flow.
- If non-urgent patients have been seen in the urgent care or fast-track area, does that mean all patients classified as ESI level 4 and 5 may be triaged to fast-track? Can some ESI level-3 patients also go to the fast track?
- Where will patients be seen who are triaged as ESI level 2 due to pain? For example, on a busy afternoon, in what part of the ED is the patient with renal colic in severe pain seen? Are they placed in the last open bed even if it is monitored? In an ED with several different sections, do they have to go to a specific section?
- Some EDS are using a licensed independent provider in the triage area. The provider's role is to see and treat low acuity patients and discharge them from triage. Is this a process your department is considering? If a two-tier triage process is being used, and the patient's first contact is with the greet nurse, does the greet nurse assign an ESI level for just ESI levels 1 and 2?

The ED leadership team will ultimately make these policy decisions, but the implementation team should identify these issues and make recommendations.

The ESI research team is frequently asked whether the ESI system includes criteria for a time to reassessment by triage level. The ESI system does not include reassessment recommendations. This is a key difference between ESI and other five-level triage systems. The ESI triage research group purposefully did not identify reassessment times but left that to individual departments to incorporate into their triage policy. The group urges caution: in this era of ED crowding it is very difficult for

busy triage nurses to reassess patients at set time intervals when they are busy sorting incoming patients. Falling short of the policy can become a departmental liability. The ED technician can take and document another set of vital signs, but the nurse must talk to the patient and evaluate the vital signs for changes. Assessment is a nursing function that cannot be delegated to unlicensed assistive personnel (e.g., nursing assistants, technicians).

It would be unrealistic for the implementation team to assume that all staff will embrace the change to ESI. Resistance is expected. Major change can trigger a wide range of emotional responses such as enthusiasm, skepticism, stress, anxiety, anger, and a sense of loss. The implementation team needs to be prepared for these reactions and not personalize them. The team should put into place strategies to minimize or manage them. Change is never easy, and the implementation team needs to "stay the course" and not give up. The team needs to openly discuss the planned change, answer questions, and gather support.

Planning ESI Education

Education for physicians, nurses, and support staff is one of the critical tasks that the implementation team needs to consider. ED leadership must commit the resources to thoroughly prepare the ED staff to use ESI. Several key concepts need to be understood to maintain the reliability and validity of the instrument. Some form of education about the ESI should be provided to all staff who will use the ESI information, including ED nurses, physicians, and other providers; unlicensed assistive personnel; and clerical staff. While the triage nursing staff will need a full orientation to the ESI, other staff will need less education. The original ESI hospitals have found that successful implementation of the ESI requires every triage nurse to attend, at minimum, a two- to four-hour education program. At University of North Carolina Hospitals, clerical and nursing assistant staff members received a memo describing the five ESI categories and notice of the implementation date.

The physician on the implementation team may choose to handle physician education. The duration of physician orientation to ESI will depend on how familiar they are with the algorithm. At teaching hospitals, the ED residency director needs to allocate time for a member of the implementation team to provide an orientation for the residents. It is helpful to give residents copies of key ESI research articles for review (see Chapter 1, "Introduction to the Emergency Severity Index: A Research-Based Triage Tool"). With more hospitals using physicians at triage, it is even more important that physicians have a solid understanding of the five levels of the ESI triage system.

The education program is best conducted in a setting away from the ED that is free from the distractions of the clinical area and conducive to learning.

Implementation may be an opportunity for collaboration. For example, two hospitals chose to change to ESI at the same time and decided to pool resources. They offered joint educational programs.

Two to four hours is a realistic timeframe for the triage nurses' ESI educational program. The educator or clinical nurse specialist should set the day and time for education. Plans should include one or two make-up classes for the triage nurses who are ill, are on vacation, or are pulled from the class and back into clinical duties due to staffing issues.

The implementation team must identify one or several trainers for the orientation to ESI. It may not be realistic to have an educator available to teach all classes. Many groups use a train-the-trainer program, which initially trains team nurses who feel comfortable teaching and confident dealing with questions and resistors in the group. An experienced educator should be available during the initial sessions to ensure accuracy of the information provided and to assist the trainer if needed.

Experienced educators have found that reading the research publications can be particularly helpful in explaining why the change to ESI is so important.

Low-cost training opportunities are available for EDs to consider in implementing ESI:

- The Emergency Severity Index Online Course, 4th Edition
- *Emergency Severity Index (ESI) Version 4 Implementation Handbook, 2020 Edition.* This document is for hospitals with staff who have less curriculum development experience.

The ESI Online Training Course

The ESI Triage Research Team recognized that staff attendance at a 2–4 hour program is often difficult to organize. In addition, some hospitals have chosen to conduct train-the-trainer programs and found that there were inconsistencies in the information presented by different trainers. As a solution, in 2009 the ESI Research Team developed an online education program that is interactive, inexpensive, and self-paced. That course was refreshed and made more interactive in 2020.

The program highlights some of the nuances of the ESI that novices find challenging. The handbook is incorporated into the numerous learning activities that reinforce some of the key concepts or critical decision points. The advantage of this type of self-directed, self-paced learning is that a nurse can take the course at his or her own pace and is actively engaged by the content and review exercises. On completion, the participants receive the course post-test results and a completion certificate.

The ESI web sites also include many resources and much information about ESI. To learn more about the online course, go to https://www.ena.org/education/esi.

Locally Developed ESI Training

Many EDs develop their own educational programs using the ESI Handbook, as well as additional information relevant to triage at the local facility or hospital.

Basic ESI training can take between two to four hours. Many hospitals use this opportunity to review other triage-related information, such as high-risk situations or policy and procedure changes. The following section provides a detailed description of a two-hour training segment of ESI. It is advised that the trainers review the entire *ESI Implementation Handbook* prior to developing their own content. This will help assure reliability and validity of the ESI algorithm.

Section 1: Introduction

The introduction explains why the department has chosen to adopt ESI. The issues with the former triage acuity system should be briefly explained along with how ESI will address them and the advantages of ESI. The time allocated for this section will depend on what information has already been shared with staff. It is important for the trainer to focus on what ESI will do for the staff nurse and for ED administration.

A number of reasons can be cited to support a move to ESI:

- Increases in local ED volume, change in admission rate
- Desire to use a reliable and valid triage system
- Changes in ED patient population
- More trauma patients
- More psychiatric patients
- Changes within the hospital that have affected the ED
- Beds closed
- Unit renovations
- Holding patients in the ED
- Increased length of ED stay for admitted patients
- Nationwide trends
- Increase in the number of elderly patients
- Increase in the number of patients seeking primary care in the ED
- Increase in the number of uninsured seeking care in the ED
- Nursing shortage

At the end of the introduction, trainers should discuss the issues with the current triage acuity rating system that the ED may have already identified. These may include mistriages. While it is important to include specific examples of problems the department has experienced with the current triage system, it is also important that the trainer not let this become a "gripe" session. The facts should be presented, and any comments or questions can be addressed at the end of the program.

If the staff is not convinced that a change in the triage acuity rating system is necessary, they can play the **Triage Game** before discussing the importance of reliability and validity of triage systems.

The Triage Game

The Triage Game is a way to break the ice and illustrate the poor inter-rater reliability of the three-level triage acuity rating system. Each nurse in attendance is given a packet consisting of red, yellow, and green colored cards. The red card is labeled "emergent," the yellow "urgent," and the green "non-urgent." Three cases are read to the group, and after each case participants are asked to rate the patient acuity and hold up the appropriate card. Each participant is able to see how other members of the group rated the patient. Resistance decreases as the group begins to notice that participants rate the same patient differently. The group begins to realize that with a three-level system there is always some level of disagreement within the group.

Three cases that could be used for this game follow:

Case 1. A 57-year-old woman presents to the ED with epigastric pain rated as 6/10. She is a smoker, and her only medication is for high cholesterol. She has been tired for the last week and thinks she just needs a vacation. Her skin is cool and clammy. Is this patient emergent, urgent, or non-urgent? This case may generate some interesting discussion. Chances are many of the group will triage the patient as urgent. Some more experienced staff may recognize that she is probably having a cardiac event and will label her emergent.

Case 2. A 36-year-old female presents to the ED with left lower quadrant abdominal pain 6/10, vaginal spotting, last menstrual period 8 weeks ago, and vital signs within normal limits. Is this patient emergent, urgent, or non-urgent? Is this patient pregnant? Does she have an ectopic pregnancy? These are questions the group may ask as they try to assign a triage priority. Many participants will assign her to the urgent category, while a few may think she is emergent.

Case 3. A 10-day-old baby boy is brought to the ED by the parents because he feels warm and is not nursing well. Mom thinks he has the bug that her other kids are getting over. His rectal temperature is 38.3°C (101°F). Is this patient emergent, urgent, or nonurgent? Some nurses may accurately say he is emergent, recognizing that fever in a 10-day old is concerning. Others will say he is merely urgent because the temperature o is not that significant in light of the other kids having been sick.

After the Triage Game, it is useful to highlight the research on poor inter-rater and intra-rater reliability of conventional three-level triage systems, which is described in Chapter 1. At this point the group is about 15 to 20 minutes into the presentation and staff should be ready to hear about ESI. Participants should have a copy of the front and back of the algorithm (see the cards on the back cover of the handbook). The trainer can now begin the discussion.

Section 2: The ESI Algorithm

This section of the presentation explains the algorithm in detail. It is important to stress to course participants that ESI was

developed by a group of emergency nurses and physicians and has been in use at a number of hospitals since April 1999. Other important background information to discuss includes the following points about ESI:

- The program is based on research.
- Consistent use of the ESI by all staff is more likely when all triage nurses participate in a standardized educational program.
- ESI enables rapid sorting into one of five categories.

Begin review of the algorithm with the conceptual version so that the four major decision points can be reviewed. Then begin a detailed description of the algorithm itself. The instructor should walk through each decision point slowly and not move on to the next decision point until all questions and concerns are addressed. This section will take 40–65 minutes depending on the size of the group and the experience of participants. For each decision point, the trainer should review the questions the triage nurse should be asking.

Decision Point A

Does this patient require immediate lifesaving intervention? If the answer is yes, the patient is assigned to ESI level 1. It is imperative that the instructor spend time reviewing the notes for box A on the back of the algorithm card. The instructor should also include examples of ESI level-1 patients and the reasons they fall into that triage level. Experienced ED nurses have no problems identifying this group of patients.

Decision Point B

Is this a patient who should not wait? The trainer needs to discuss in detail the three questions that are part of Decision Point B:

- Is this a high-risk situation?
- Is there new onset confusion, lethargy, or disorientation?
- Is this patient in severe pain or distress?

Is this a high-risk situation? Define the term "high risk" and have the participants identify chief complaints or diagnoses that are high risk. Participants will usually mention abdominal aortic aneurysm and ectopic pregnancy, but the trainer needs to encourage the staff to think about other low volume, high-risk presentations. During this discussion, knowledge deficits may become evident and the instructor will need to provide additional educational materials. For example, staff nurses may disagree on the need for immediate evaluation of a patient that presents with symptoms of central retinal artery occlusion. This is a perfect opportunity to explain why this is high-risk situation. A discussion of high-risk situations also provides the trainer with an opportunity to review triage red flags in the elderly and in children.

To prepare for this section of the course, the instructor may want to review the *Emergency Nursing Core Curriculum* (Emergency Nurses Association, 2018) or other emergency nursing professional books or textbooks and develop a list of high-risk patient situations. These situations are outlined in Chapter 3, "ESI Level 2," (Table 3-1). The instructor needs to stress that a high-risk patient may be safe to wait up to 10 minutes while an open bed is found.

Is there new onset confusion, lethargy or disorientation? This question also needs to be reviewed using examples from various age groups (see Table 3-1 and case studies in Chapter 9, "Practice Cases," and Chapter 10, "Competency Cases"). The definition of "acute" change in level of consciousness is important to clarify.

Is this patient in severe pain or distress? The concept of severe pain or distress elicits many opinions and questions from the audience. The instructor should not engage in a debate about pain scales and their use at triage. The discussion should focus on the intent of this question to identify the patient in extreme pain. It may be helpful to explain that there are actually three components to severe pain:

- The patient's rating of pain is 7/10 or greater.
- The nurse's assessment, including chief complaint, subjective and objective assessment, past medical history, and current medications.
- Can the triage nurse perform any nursing interventions that may decrease this patient's pain? (Examples: ice, elevation, positioning, quiet room, something to cover their eyes, and medications.)

If the patient rates their pain as 7/10 or greater and the triage nurse feels this patient cannot wait and needs intravenous analgesia, the patient will be assigned to ESI level 2. Participants may have many questions about this concept, and the trainer needs to stress that it is not just the patient's pain rating that makes the patient an ESI level 2. This concept is discussed in detail in Chapter 3. Nurses may say they feel uncomfortable documenting a patient's high pain rating and then leaving the patient in the waiting room. It is important for the instructor to stress that the patient's rating is one piece of an assessment and that the nurse should accurately document what he or she is observing. For example: "Rates pain as 10/10, skin warm and dry, laughing with friend at triage," or "Generalized abdominal pain for 3 days, constant dull ache. Rates pain as 10/10."

The instructor should describe several patients that meet ESI level-2 criteria due to pain. Examples include sickle-cell crisis, a cancer patient with breakthrough pain, and renal colic. At the same time the instructor needs to address patients who probably will not be assigned to ESI level 2 due to pain. Examples include toothache, eye pain, most headaches, and extremity injuries. This is a great opportunity to discuss nursing interventions at triage to minimize or decrease a patient's pain. This discussion may also prompt the recognition of standing orders for analgesic medications at triage.

The next area to address is physiological or psychological distress. Examples are often the best method of explaining this concept. Examples of physiological distress include urinary retention and priapism. These patients are in acute distress and require immediate intervention. Many psychiatric emergencies fall under psychological distress. Examples include sexual assault, domestic violence, paranoia, and manic behavior. The suicidal/homicidal patient has already been assigned to ESI level 2 because they are high risk. These patients should be assigned to ESI level 2 even if they come in every day stating they are going to hurt themselves or someone else. This is an excellent opportunity to review your ED psychiatric policy.

After discussing the three questions under decision point B, it is helpful to review all the level-2 criteria together. Once again, a list of examples is helpful.

Decision Point C

How many different resources will this patient consume? It is important to clarify what is and what is not a resource. Reviewing the resource table on the back of the algorithm card usually generates questions and discussion. The following discussion includes examples of typical questions the trainer should be prepared to discuss.

> ***Course participant:*** Why isn't an interpreter a resource? We use them all the time.
>
> ***Trainer:*** It is important for the nurse using ESI not to become overly focused on differentiation of what is and what is not a resource. ESI is a triage acuity rating system that evaluates how ill or injured a patient is on presentation to the emergency department. The need for an interpreter does not change that. Inclusion of everything as a resource will not enable differentiation of triage levels.
>
> ***Course participant:*** I don't understand why crutches aren't a resource. Fitting a patient correctly and teaching crutch walking takes time.
>
> ***Trainer:*** ESI assesses acuity on presentation to the emergency department, not workload issues. If crutch walking instructions counted as a resource, all patients with sprains would now be triaged as ESI Level 3: radiograph and crutch walking. This would clearly defeat the purpose of ESI.
>
> ***Course participant:*** A patient who needs a blood test and urine test will consume two resources.
>
> ***Trainer:*** This is only one resource. For example, a urinalysis and a urine culture is one resource: laboratory study. A urinalysis and two blood tests are one resource: laboratory study. A vaginal culture and a blood test are one resource: laboratory study.
>
> ***Course participant:*** Why isn't a pelvic exam a resource? They take staff time.
>
> ***Trainer:*** As we discussed, a physical exam is not a resource. For the female patient with abdominal pain, a pelvic exam is part of that physical exam. Just like the patient with an eye complaint, a slit lamp exam is part of the physical exam for that chief complaint.
>
> ***Course participant:*** I don't understand why security is not on the list of what is a resource. We use them all the time with our psychiatric population.
>
> ***Trainer:*** Security is used to monitor psychiatric patients when they have been determined to be a danger to themselves, others, or the environment or when they are in acute distress. Because they are high risk, these patients meet the criteria for ESI level 2 as high risk. Remember, resources are only looked at after the triage nurse has determined that the patient does not meet the criteria for ESI level 1 or 2.

Once the group understands the concept of resources, it is important to give multiple examples of patients who would be assigned ESI level 4 and 5. Before discussing ESI level 3, the trainer needs to review decision point D.

Decision Point D

What are the patient's vital signs? It is important that participants understand that the triage nurse should consider the patient's vital signs. The triage nurse uses his or her judgment to determine whether the patient should be uptriaged to ESI level 2 based on abnormal vital signs. It is important to present examples of patients the triage nurse should up-triage to ESI level 2, as well as examples of ESI level-3 patients who do not require up-triage based on abnormal vital signs.

At the end of this segment, the participants should be quite comfortable with the type of patients that fall into each ESI level. Reviewing practice cases will reinforce use of the algorithm and answer many questions.

Section 3: ESI Practice Cases

After a thorough description of the ESI algorithm, patient scenarios are used as a group teaching tool. Chapter 9 lists many cases specifically written for practice and intended to simulate an actual triage encounter. The cases encompass all age groups and the complete spectrum of acuity. In addition, these cases illustrate most of the important points in the algorithm. The instructor reads each case, and the participants are asked to use the algorithm to assign an ESI level. Each participant can be given an additional packet of colored cards, such as those used in the Triage Game, labeled ESI levels 1 through 5 and be asked to hold up the appropriate card as each case study is discussed. The advantage of using the cards is that participants will begin to notice a higher degree of agreement with ESI than they observed with the three-level triage system.

Once everyone in the group has assigned an ESI level, the trainer can proceed with a step-by-step review of how the level was

determined. The research group found it helpful to instruct nurses to always start with decision point A and work through the algorithm. If the case moves to decision point C, it is helpful to have the participants verbalize the expected resources. Many misconceptions can be cleared up with this strategy.

As previously discussed, staff may initially have difficulty with what is and what is not a resource and with determining the number of resources. This is a perfect opportunity to re-emphasize the definition of resources in the ESI triage method and answer the "what about" questions.

Section 4: Competency Cases

One question managers and educators frequently hear is, “How do you know your staff is competent to perform triage?” Chapter 10 was written with this question in mind. The chapter includes two sets of cases for each nurse to review and assign a triage acuity rating using ESI.

Each nurse should complete the competency cases individually and return them to the trainer to assess for accuracy. The ED management and educational staff of each hospital must define parameters for a passing score prior to assessing staff competency. For the staff nurse whose score falls below the acceptable level, re-education is indicated, and competency should be re-assessed at a later date with different cases. Paper case assessment of competency only addresses the staff nurse's ability to assign a triage acuity rating to paper cases. An evaluation of each triage nurse performing triage with real patients and using the ESI criteria should be performed with a triage preceptor or other designated expert.

Strategies to Assist with Implementation

Strategies that the ESI triage research group have found useful for successful ESI implementation include the following:

- E-mails to remind staff of the upcoming change
- Computer help screens to explain the five ESI levels during triage data entry
- Posters to address questions about ESI after implementation
- Informal chart reviews conducted by the trainer, clinical nurse specialist, or ESI champions focusing on the finer points of the algorithm

Reinforcement is key to the successful implementation of ESI. At Brigham and Women's Hospital and the York Hospitals, the implementation teams chose to have the algorithm preprinted on progress notes. For two months the triage nurse was required to use a progress note and record the patient's chief complaint and circle the assigned ESI level. The progress note served to make the triage nurse look at the algorithm each time a patient was triaged.

Questions and misinterpretation of the finer points of the algorithm will always arise after implementation and will need to be addressed with re-education. After implementation of ESI at Brigham and Women's Hospital, it was noted that the staff were not consistently assigning an ESI level 1 to intoxicated and unresponsive patients. This point was emphasized on a poster in the break room to bring attention to the problem.

Implementation Day

The implementation team needs to be available around the clock to support the triage staff, answer questions, and review triage decisions. It is important that mistriages be addressed immediately in a non-threatening manner. Making staff aware ahead of time that this will be taking place is less threatening. Reinforcing the efforts of the staff and being available will help ensure ESI is appropriately integrated into the ED.

Post-Implementation

Following implementation it is important that triage nurses continue to be vigilant when assigning triage acuity ratings. Many nurses may complain that more patients are ESI level 2. Triage nurses should be reminded not to deviate from the original algorithm but instead to understand the value of ESI as an operational tool. The staff should understand that deviations from the algorithm will threaten the reliability and predictive validity of the tool.

Staff efforts in making a smooth transition to ESI should be recognized and rewarded. This could include an article in the hospital newspaper or a note of thanks to the staff from the ED leadership team. Successful implementation of ESI requires a dedicated team that recognizes the degree of change and effort needed to change triage systems. The team must be able to develop and carry out a specific, simple, and realistic plan. The team leader should have a clear vision, be able to clearly articulate it, be committed to the ESI implementation, and be able to energize the other members of the team and the staff. The team needs the support of the ED leadership and the resources necessary to make this planned change. For this change to be successful there must be broad-based support beginning with the most senior levels of the institution.

> **NOTE**: Appendix A, “Frequently Asked Questions and Post-test Materials for Chapters 2–8,” of this handbook includes frequently asked questions and post-test assessment questions for Chapters 2 through 8. These sections can be incorporated into a locally developed ESI training course.

References

American College of Emergency Physicians, & Emergency Nurses Association. (2017). *Triage scale standardization* [Joint policy statement]. Author. https://www.ena.org/docs/default-source/

resource-library/practice-resources/position-statements/supported-statements/triage-scale-standardization

Grol, R., & Grinshaw, J. (1999). Evidence-based implementation of evidence-based medicine. *Joint Commission Journal on Quality Improvement*, *25(*10), 503–513. https://doi.org/10.1016/s1070-3241(16)30464-3

Nelson, R. (2002). Major theories supporting health care informatics. In S. P. Englebardt and R. Nelson (Eds.), *Health care informatics: An interdisciplinary approach* (pp. 3–27). Mosby.

Sweet, V. (Ed.). (2018). *Emergency nursing core curriculum core* (8th ed.) Elsevier.

8

Evaluation and Quality Improvement

To maintain reliability of the Emergency Severity Index (ESI) in an individual institution, it is important to evaluate how the system is being used. A natural learning curve will occur, and it can be easy for nurses to fall back into maladaptive triage habits or become concerned about triaging "too many level-2 patients" when the waiting room is crowded. Additionally, new models of triage intake are being used by EDs across the United States. Physicians, nurse practitioners, and physician assistants may play a role at triage. It is important that anyone who performs the triage assessment and is responsible for assigning a triage level upon presentation be competent in ESI. Continuous evaluation using standard quality improvement (QI) methods will help ensure that the reliability and validity of the system is maintained by all.

In 2001, the Institute of Medicine (IOM) published the report, *Crossing the Quality Chasm, A New Health System for the 21st Century*, which defined quality healthcare and identified six aims to improve the overall quality of healthcare (IOM, 2001). The IOM defined quality healthcare as, "The degree to which health services for individuals and populations increase the likelihood of desired health outcomes and are consistent with current professional knowledge." The six aims of quality include improving the safety, effectiveness, patient centeredness, timeliness, efficiency, and equity of the healthcare system. They are defined in Table 8-1 (IOM, 2001). The triage process is probably one of the highest risk areas in the ED, and attention to quality monitoring is important. All six aims can be used to evaluate the triage process. Emergency departments can structure their quality improvement (QI) monitoring process around any or all of the six IOM aims. Specific examples are discussed later in the chapter.

It is also important to choose a system that enables the improvement to be readily assessed. When choosing a method to evaluate the success or failure of implementation, it is important to remember why the triage process was changed. The following reasons are frequently identified as driving forces for change existing triage processes:

Table 8-1. The Six Institute of Medicine Aims

IOM Aim	Definition
Safety	Avoiding injuries from care that is intended to help
Effectiveness	Providing services based on evidence and avoiding interventions not likely to benefit
Patient-Centeredness	Respectful and responsive to individual patient preferences, needs, and values in clinical decision-making
Timeliness	Reducing waits, and sometimes harmful delays, for those who receive care
Efficiency	Avoiding waste, in particular, of equipment, supplies, ideas, or energy
Equity	Care that does not vary in quality due to personal characteristics (gender, ethnicity, geographic location, or socio-economic status)

- Reduction in variation of assigned triage categories and the ability for everyone to "speak the same language" regarding triage categories
- Decreased risk of negative outcomes due to mistriage, particularly while patients are waiting
- The ability to obtain more accurate data to use for administrative purposes
- The need to move from a three-category to a five-category triage system to better "sort" the increasing number of ED patients
- A more accurate description of patient triage levels and departmental case mix (Wuerz, Milne, Eitel, Travers, & Gilboy, 2000)

The ultimate goal of ESI implementation is to accurately capture patient acuity to optimize the safety of patients in the waiting room by ensuring that only patients stable to wait are selected to wait. Patients should be "triaged" according to acuity of illness. When a reliable and valid triage system such as ESI is used, the triage score can then be used as administrative data to

accurately describe departmental case mix, beyond admission and discharge status. With this in mind, it is important that every patient be assigned a triage score on arrival. The primary goal of conducting QI activities for the ESI triage system is to maintain reliability and validity of the system implementation. If triage nurses are not assigning scores accurately, then the data cannot be used for any purpose, either real time or for other administrative purposes. With the addition of new nurses or other providers at triage, and natural trends over time, it is important to, at a minimum, always monitor the accuracy of the triage level. It is also important to clearly articulate to the ED staff what is not a goal of ESI triage implementation. For example, ESI triage alone cannot decrease the ED length of stay or improve customer satisfaction with the ED visit.

ESI Triage Quality Indicators and Thresholds

In any QI plan, it is important to select meaningful indicators to monitor. Donabedian's trilogy of structure, process, and outcome (Table 8-2) can be used to select the type of indicator (Duff, 1992). All indicators can be organized around Donabedian's structure and the six IOM aims of improving quality care: safety, effectiveness, patient centeredness, timeliness, efficiency, and equity. Selected examples are included in Table 8-2 and Table 8-3. The tables include indicators specific to monitoring implementation of ESI and suggest other indicators that can be used to evaluate other aspects of the broader triage process.

Table 8-2. Donabedian's Trilogy

	Definition (Example)
**Structure**	How care is organized (standing protocols that allow nurses to give antipyretics for fever at triage)
**Process**	What is done by caregivers (proportion of patients with fever at triage who receive antipyretics at triage)
**Outcome**	Results achieved (fever reduction within one hour after arrival)

While selecting quality indicators to review is critical, it is also important to recognize specific indicators that are not appropriate to review. For example, the actual number of resources that were used in providing care to the patient is ***not*** an appropriate quality indicator to monitor. Resources are incorporated in the ESI algorithm only to help the triage nurse to differentiate among the large proportion of patients that are not critically ill. Monitoring the number of resources used "on the back end" may further increase the triage nurses' focus on counting resources, which is not the most important component of the algorithm. However, knowledge of the standard of care will serve to increase accuracy in assessing the resources used for various presentations, enabling more accurate triage assignment.

In addition to selecting useful quality indicators, it is also important that the ED management team select a realistic threshold to meet for each indicator. All indicators do not need to have the same threshold. For example, when reviewing accuracy of triage categorization, a realistic goal must be determined. Should the triage category be correct 100 percent, 90 percent, or 80 percent of the time? Frequently a threshold of 90 percent is selected. However, the goals and circumstances of each department may be unique and should be considered when selecting each indicator and threshold. For example, the ED management team might stipulate that, when in doubt about a patient's triage rating, nurses err on the side of overtriage. While this approach might result in some patients being mistriaged as more acute than they actually are, it is preferable to risking an adverse event because the patient was triaged to a less urgent category. In this ED, the triage accuracy threshold might be 80 percent, with a goal to keep the mistriage rate at 20 percent.

Finally, it is also important to determine how many triage indicators should be monitored on an ongoing basis. It is reasonable to select one or more indicators. The number of indicators to be monitored will be determined by available staff resources and the relationship of ESI indicators to other quality indicators that are routinely monitored. It is also possible to focus on monitoring one aspect of triage for a period of time and then switch to another indicator when improvement occurs in the previously monitored indicator. Various levels of indicators could also be measured, e.g., shift-level or a day-of-week level of evaluation.

Accuracy of triage acuity level should probably be monitored on a continuous basis to evaluate new triage nurses as well as monitor for trends that may identify the need for re-education on a particular aspect of triage. These data can be reported as the proportion of correctly assigned triage levels. In addition, a more formal evaluation of inter-rater reliability can be periodically conducted by having a proportion (example, 20%) of randomly selected nurses from all shifts assign triage levels to pre-selected paper cases. It is recommended that at least 10 paper cases are used for this type of evaluation. This evaluation will measure how often the triage nurses in an individual department would assign triage levels the same or would "agree." This can be a valuable exercise to conduct on a regular basis (e.g., after key changes in departmental processes) if resources are available. It may also be appropriate to evaluate triage acuity accuracy more often in a department with higher nurse turnover.

Table 8-3. Possible Triage Quality Improvement Indicators

IOM Aims	Structure, Process, Outcome	Indicator	Data Source/Method
**Safety**	Structure	Implementation of ESI (reliable and valid system)	Administrative process
	Process	Assignment of correct ESI triage level (under- and overtriage levels)	Review of triage note by triage expert
	Outcome	Review of all negative outcomes	Review by internal QI or triage committee
**Effectiveness**	Structure	Implementation of nurse initiated analgesic protocol at triage	Administrative policy
	Process	Proportion of patients with pain eligible for analgesics at triage that received them	Medical record review
	Outcome	Decrease in patient reported pain score within 30 minutes of arrival	Medical record review
**Patient Centeredness**	Process	Documentation of a subjective statement by the patient describing reason for visit	Medical record and triage note
**Timeliness**	Process	Time of arrival to time to physician evaluation	Medical record review
**Efficiency**	Structure	Staffing policy to enable flexibility in nurse staffing pattern to meet the demands of changing influx of patients at triage	Administrative policy
	Process	Increased capability of nurses to float to triage during increased influx and move to other patient care areas when triage demand is low	Staffing pattern log reviews
		Length of stay per ESI triage level	Medical record and triage note
		Admission rates per ESI triage level	Medical record and triage note
		Review of all ESI level 4 and 5 cases admitted	Medical record and triage note
**Equity**	Process	All patients eligible for analgesics at triage according to the protocol receive them, regardless of gender or race	Medical record and triage note

ESI Triage Data Collection

The method of collecting QI data for ESI triage indicators can be incorporated into the data collection process for other ED quality improvements or data can be collected as a separate process. The method of data collection will depend on the indicator selected, the availability of triage experts, and logistic issues such as accessibility to electronic versus paper ED records. For example, if "accuracy of triage category" is selected as a triage quality indicator, a triage expert is needed to review the triage categories.

Accuracy of the triage category assigned is a critical indicator and should be monitored when ESI is first implemented. If it is determined that the institution wishes to measure ED length of stay or wait times to see the physician for each ESI triage category, it is preferable to have access to electronic information to successfully monitor this indicator. Without electronic sources, these data are cumbersome to track, and manual calculations most likely result in error. It is also advisable to monitor medians instead of means when evaluating any indicator that is a time measure (e.g., time to physician assessment). The mean is usually the preferred index of central tendency because it is based complete information. Its computation includes the exact values of each data point in the data. At the same time it is also the index most sensitive to the skewing caused by outliers. The median is not affected by outliers. Standardized time interval nomenclature is starting to appear in the literature. However, it is important to reiterate that ESI does not stipulate times to care. Finally, when monitoring quality indicators, it is important to determine how many charts must be reviewed for each indicator and how frequently the indicator should be reviewed (monthly, quarterly, other). Selection of the appropriate number of charts for each indicator will depend on the particular indicator. If wait times for each category are reviewed, data will be most accurate when a large percentage of cases, preferably all, are reviewed.

Routine evaluation of the accuracy of ESI should reflect an appropriate number of randomly selected charts. Cases from different nurses and each shift and day of the week should be reviewed. Ten percent of all cases is often selected as an appropriate number of cases to review. In a busy ED, this is often an unrealistic number. It is important for each institution to consider the number of review staff, their backgrounds, and their availability. It is also prudent to evaluate ESI rating

accuracy for individual cases where there was a near-miss or an adverse event related to the triage process.

When determining the frequency of triage audits, the institution should consider other departmental QI activities and try to integrate the review of triage indicators into the same process and schedule.

It is also very helpful to involve the triage nurses in data collection. Peer reviews are a useful way to raise awareness about triage accuracy.

Sharing Results and Making Improvements

In the vast majority of cases, any attention given to QI and process improvement activities is given to the monitoring stage of the process, and little attention is paid to evaluating the data and determining process improvements. The "numbers"" are often posted somewhere, and little is done to actually improve the outcomes. The most important component of QI is sharing the data and discussing ways to improve the results. Positive systems outcomes in triage improvement depend on measuring, analyzing data, and then educating the staff. All staff should be aware of the triage quality indicators, the current overall incidence in which the threshold is met, and the actual goal. For example, if the accuracy of the triage category is being monitored and continues to be reported as 60 percent, intervention is necessary.

Examples of ESI Triage Indicators

The EDs described in the following have implemented ESI and a QI program. They have provided examples of how they incorporate triage indicators into their QI plan.

Hospital 1

At Hospital 1, the accuracy of triage nurses' ESI triage ratings is assessed on a continuous basis and reported quarterly as one indicator of the overall ED QI plan. This indicator has been monitored since ESI was implemented and continues to be the only triage indicator monitored to date. Each week, three different nurses randomly select five charts to review with the ED clinical nurse specialist (CNS). The assessment team reviews many different general documentation indicators, including the accuracy of the ESI triage category. The CNS is the designated triage expert and discusses each case with the staff nurse as she reviews the records. When there is a disagreement, cases are reported as mistriages for the QI report. The assessment team collects and retypes all mistriages as an educational tool that includes an explanation of the correct triage category. These cases are compiled in a handout and distributed to all staff nurses monthly. The assessment team reviews sixty charts monthly.

Hospital 1 has noted several distinct advantages of the triage accuracy review:

- All ED staff nurses are aware of the QI indicators. Case examples provide individual nurses with the opportunity to reflect on their own practice with similar case scenarios. Staff nurses have the opportunity to discuss each case with the CNS to obtain additional insight.
- All nurses benefit from the discussion when the cases are distributed as a teaching tool.

Hospital 1, like many other EDs, also has excellent information technology resources that facilitate quality monitoring of clinical information. The triage acuity is part of the electronic medical record. It is possible to track time to physician evaluation for each triage category. This can be powerful administrative data. This data is far more powerful when describing overall ED acuity than when using hospital admission data to describe overall ED acuity.

Hospital 2

At Hospital 2, several triage indicators are reviewed on a regular basis. The ESI rating assigned by the nurse at triage and time data are recorded in the hospital's computer information system during the ED visit. The electronic information is compiled for monthly QI monitoring. Time data are reported by ESI triage level, including the following:

- Total ED length of stay
- Time from triage to placement in the ED bed
- Time from triage to being seen by the ED physician
- Time from placement in the ED bed to discharge

The time data are used for many purposes, such as monitoring for operational problems that lead to increased length of stay. The time data prove useful in addressing issues related to specific patient populations at Hospital 2's ED. For example, the time data were tracked for psychiatric patients and subsequently a new policy regarding psychiatric consults was developed. The policy stipulates response times for the crisis team to see ED psychiatry patients and is based on ESI triage level. Information about the number of patients triaged to the various areas of the ED (medical urgent care, minor trauma, pediatrics, acute) is also reported by ESI triage level on a monthly basis. These data are used to make operational decisions, such as the time of day that medical urgent care and minor trauma services are offered.

The accuracy of triage nurses' ESI ratings is reviewed as part of the QI program at Hospital 2. The initial review was conducted during the first few months after implementation of the ESI. The nurse educator reviews a random sample of ED charts on a regular basis to assess the accuracy of the triage nurses' ESI ratings. Individual nurses receive feedback and trends are reported to the entire nursing staff.

Accuracy of triage ratings are also reviewed at Hospital 2 through a monthly peer chart review process. Each nurse selects two random ED charts per month and reviews many aspects of nurses' documentation, including the ESI triage rating. The

review is forwarded to nursing leadership for follow-up with individual nurses. Any important trend identified is communicated to the entire staff.

Another QI effort at Hospital 2 is the review of all ESI level-3 patients triaged to the medical urgent care (fast track) area. The nurse manager receives a monthly report, compiled with electronic data from the hospital computer system, of all ESI level-3 patients triaged to medical urgent care and all ESI level-4 and level-5 patients triaged to the ED. Though the department has a guideline that ESI level-4 and level-5 adult patients are primarily triaged to medical urgent care or minor trauma, and ESI levels-1, -2, and -3 adult patients are primarily triaged to the acute ED, the triage nurse is allowed discretion in triaging these patients. The ongoing review of the ESI level-3 patients sent to medical urgent care enables the leadership team to review the accuracy of the nurses' triage decisions.

Hospital 3

At Hospital 3 the manager assigns experts to review triage categories. The manager and clinical coordinators review charts identified by peers as potential mistriages. The expert group reviews the chart and discusses it with the triage nurse. The team of experts spot check charts frequently. If a trend is noticed, the expert group will post the case so that all staff can learn from it.

Hospital 4

At Hospital 4, the manager created a log after initiation of the ESI triage system. The triage nurse logged the patient name, triage nurse name, triage level, and rationale and resources for each patient triaged. The management team reviewed each chart for triage category accuracy, either while the patient was in the department or the next day. The management did this for the first two weeks and again in three months. The purpose of this monitoring activity was to assess the triage nurses' understanding of resource definitions.

Hospital 5

Hospital 5's strategic plan called for the hospital to increase the number of trauma and stroke patients they would accept from outlying hospitals. Most of these patients were ED-to-ED transfers. Many of these patients arrived intubated and others were intubated on arrival. The staff felt that the acuity of the ED patient population was rising quickly. Nursing leadership chose to look at case mix data (the number of patients in each ESI category) for 1 year and was able to make staffing adjustment to cover increases in patient acuity. This is an excellent example of why it is so important that every patient, including trauma and cardiac arrest patients, receive an accurate triage category. This enables each hospital to benchmark their case mix data with other institutions. These patients are clearly not "triaged" at "triage," but they represent an important group of patients seen in the ED. If the primary nurse does not assign a triage category to these patients, the ED case mix data will significantly underrepresent the higher acuity of the department's case mix.

Summary

It is important for the emergency department nursing leadership to implement a QI plan. The plan should generate meaningful data that can be shared with the ED staff on a regular basis. Issues with individual triage nurses must be promptly identified and education provided. General trends also must be identified rapidly and addressed with an appropriate response including communication with senior level leadership to plan for change. The members of the ESI research team are repeatedly asked about quality assurance, and our suggestion is to keep it simple, relevant, and meaningful.

NOTE Appendix A, "Frequently Asked Questions and Post-test Materials for Chapters 2–8," includes frequently asked questions and post-test assessment questions for Chapters 2 through 8. These sections can be incorporated into a locally developed ESI training course.

References

Duff, P. (1992).Structure, process and outcome. *Nursing Standard*, *7*(11), 4–5. https://doi.org/ 10.7748/ns.7.11.4.s71

Institute of Medicine (2001). *Crossing the quality chasm: A new health system for the 21st century*. National Academies Press. https://doi.org/10.17226/10027

Wuerz, R., Milne, L. W., Eitel, D. R., Travers, D., & Gilboy, N. (2000). Reliability and validity of a new five-level triage instrument. *Academic Emergency Medicine*, *7*(3), 236–242. https://doi.org/10.1111/j.1553-2712.2000.tb01066.x

9

ESI Practice Cases

The cases in this chapter are provided to give a nurse the opportunity to practice categorizing patients using the Emergency Severity Index (ESI). Please read each case and, based on the information provided, assign a triage acuity rating using ESI. Answers to and discussions of these cases are presented at the end of the chapter.

Practice Cases

1. "I was taking my contacts out last night, and I think I scratched my cornea," reports a 27-year- old-female. "I'm wearing these sunglasses because the light really bothers my eyes." Her right eye is red and tearing. She rates her pain as 6/10. Vital signs are within normal limits.

2. An ambulance presents to the ED with an 18-year-old female with a suspected medication overdose. Her college roommates found her lethargic and "not acting right," so they called 911. The patient has a history of depression. On exam, you notice multiple superficial lacerations to both wrists. Her respiratory rate is 10 breaths/minute, and her SpO_2 on room air is 86 percent.

3. An ambulance arrived with an unresponsive 19-year-old male with a single self-inflicted gunshot wound to the head. Prior to intubation, his Glasgow Coma Scale score was 3.

4. "I ran out of my blood pressure medicine, and my doctor is on vacation. Can someone here write me a prescription?" requests a 56-year-old male with a history of high blood pressure. Vital signs: BP 128/84 mm Hg, HR 76 beats/minute, RR 16 breaths/minute, T 36.1°C (97°F).

5. A 41-year-old male involved in a bicycle crash walks into the emergency department with his right arm in a sling. He tells you that he fell off his bike and landed on his right arm. His is complaining of pain in the wrist area and has a 2-centimeter laceration on his left elbow. "My helmet saved me," he tells you.

6. A 32-year-old female presents to the emergency department complaining of shortness of breath for several hours. No past medical history, +smoker. Vital signs: RR 32 breaths/minute, HR 96 beats/minute, BP 126/80 mm Hg, SpO_2 93% on room air, T 37°C (98.6°F). No allergies, current medications include vitamins and birth control pills.

7. "I just turned my back for a minute," cried the mother of a 4-year-old. The child was pulled out of the family pool by a neighbor, who immediately administered mouth-to-mouth resuscitation. The child is now breathing spontaneously but continues to be unresponsive. On arrival in the ED, vital signs were HR 126 beats/minute, RR 28 breaths/minute, BP 80/64 mm Hg, SpO_2 96% on a nonrebreather mask.

8. A healthy 7-year-old walks into the emergency department accompanied by his father, who reports that his son woke up complaining of a stomachache. "He refused to walk downstairs and is not interested in eating or playing." The child vomits at triage. Pain 6/10. Vital signs: T 38°C (100.4°F), RR 22 breaths/ minute, HR 88 beats/minute, BP 84/60 mm Hg, $SpO_2$100%.

9. A 6-year-old male tells you that he was running across the playground and fell. He presents with a 3-centimeter laceration over his right knee. He is healthy, with no medications and no allergies, and immunizations are up to date.

10. "I slipped on the ice, and I hurt my wrist," reports a 58-year-old female with a history of migraines. There is no obvious deformity. Vital signs are within normal limits, and she rates her pain as 5/10.

11. A 4-year-old female is transported to the ED following a fall off the jungle gym at a preschool. The fall was 4 feet. A witness reports that the child hit her head and was unconscious for a couple of minutes. On arrival you notice that the child is crying and asking for her mother.

Her left arm is splinted. Vital signs: HR 162 beats/minute, RR 38 breaths/minute.

12. A 60-year-old man requests to see a doctor because his right foot hurts. On exam the great toe and foot skin is red, warm, swollen, and tender to touch. He denies injury. Past medical history includes type 2 diabetes and psoriasis. Vital signs: T 37.4°C (99.4°F), RR 18 breaths/minute, HR 82 beats/minute, BP 146/70 mm Hg, SpO_2 99%.

13. A 52-year-old female requests to see a doctor for a possible urinary tract infection. She is complaining of dysuria and frequency. She denies abdominal pain or vaginal discharge. No allergies, takes vitamins, and has no significant past medical history. Vital signs: T 37.5°C (97.4°F), HR 78 beats/minute, RR 14 beats/minute, BP 142/70 mm Hg.

14. "I called my pediatrician, and she told me to bring him in because of his fever," reports the mother of a 2-week-old. Vital signs: T 38.3°C (101°F), HR 154 beats/minute, RR 42 breaths/minute, SpO_2 100%. Uncomplicated, vaginal delivery. The baby is acting appropriately.

15. "My right breast is so sore, my nipples are cracked, and now I have a fever. Do you think I will have to stop nursing my baby?" asks a tearful 34-year-old female. She is three months postpartum and has recently returned to work part-time. Pain 5/10. Vital signs: T 39.3°C (102.8°F), HR 90 beats/minute, RR 18 breaths/minute, BP 108/60 mm Hg,. No past medical history, taking multivitamins, and is allergic to penicillin.

16. Paramedics arrive with a 16-year-old unrestrained driver who hit a tree while traveling at approximately 45 miles per hour. The passenger side of the car had significant damage. The driver was moaning but moving all extremities when help arrived. His initial vital signs were BP 74/50 mm Hg, HR 132 beats/minute, RR 36 breaths/minute, SpO_2 99%, T 37°C (98.6°F).

17. An ambulance arrives with a 45-year-old woman with asthma who has had a cold for week. She started wheezing a few days ago and then developed a cough and a fever of 103. Vital signs: T 38.6°C (101.6°F), HR 92 beats/minute, RR 24 breaths/minute, BP 148/86 mm Hg, SpO_2 97%.

18. "I have an awful toothache right here," a 38- year-old male tells you as he points to his right lower jaw. "I lost my dental insurance, so I haven't seen a dentist for a couple of years." No obvious swelling is noted. Vital signs are within normal limits. Pain 9/10.

19. "I think I have food poisoning," reports an otherwise healthy 33-year-old female. "I have been vomiting all night, and now I have diarrhea." The patient admits to abdominal cramping that she rates as 5/10. She denies fever or chills. Vital signs: T 36°C (96.8°F), HR 96 beats/minute, RR 16 breaths/minute, BP 116/74 mm Hg.

20. "My migraine started early this morning, and I can't get it under control. I just keep vomiting. Can I lie down somewhere?" asks a 37-year-old female. Past medical history migraines, no allergies. Pain 6/10. T 36.6°C (98°F), RR 20 breaths/minute, HR 102 beats/minute, BP 118/62 mm Hg, SpO_2 98%.

21. "I cut my finger trying to slice a bagel," reports a 28-year-old healthy male. A 2-centimeter laceration is noted on the left first finger. Bleeding is controlled. Vital signs are within normal limits. His last tetanus immunization was 10 years ago.

22. "The smoke was so bad; I just couldn't breathe," reports a 26-year-old female who entered her burning apartment building to try to rescue her cat. She is hoarse and complaining of a sore throat and a cough. You notice that she is working hard at breathing. History of asthma; uses inhalers when needed. No known drug allergies. Vital signs: T 36.6°C (98°F), RR 40 breaths/minute, HR 114 beats/minute, BP 108/74 mm Hg.

23. "I'm 7 weeks pregnant, and every time I try to eat something, I throw up," reports a 27-year- old female. "My doctor sent me to the emergency department because he thinks I am getting dehydrated. T 36.1°C (97°F), RR 18 breaths/minute, HR 104 beats/minute, BP 104/68 mm Hg, SpO_2 99%. Pain 0/10. Lips are dry and cracked.

24. "I have this aching pain in my left leg," reports an obese 52-year-old female. "The whole ride home, it just ached and ached." The patient tells you that she has been sitting in a car for the last 2 days. "We drove my daughter to college, and I thought it was the heat getting to me." She denies any other complaints. Vital signs: BP 148/90 mm Hg, HR 86 beats/minute, RR 16 breaths/minute, T 36.6°C 98°F.

25. An ambulance arrives with an 87-year-old male who fell and hit his head. He is awake, alert, and oriented and remembers the fall. He has a past medical history of atrial fibrillation and is on multiple medications, including warfarin. His vital signs are within normal limits.

26. "I have this rash in my groin area," reports a 20-year-old healthy male. "I think it's jock rot, but I can't get rid of it." Using over the counter spray, no known drug allergies. Vital signs: T 36.6°C (98°F), HR 58 beats/minute, RR 16 breaths/minute, BP 112/70 mm Hg.

27. An ambulance arrives with a 17-year-old restrained driver involved in a high-speed motor vehicle crash. The patient is immobilized on a backboard and is complaining of

abdominal pain. He has multiple lacerations on his left arm. Vital signs prior to arrival: BP 102/60 mm Hg, HR 86 beats/minute, RR 28 breaths/minute, SpO_2 96%.

28. "I just need another prescription for pain medication. I was here 10 days ago and ran out," a 27-year-old male tells you. "I hurt my back at work, and it's still bothering me." Denies numbness, tingling, or bladder or bowel issues. Vital signs are within normal limits. Pain 10/10.

29. An ambulance arrives with a 32-year-old female who fell off a stepladder while cleaning her first-floor gutters. She has an obvious open fracture of her right lower leg. She has +2 pedal pulse. Her toes are warm, and she is able to wiggle them. Denies past medical history medications, or allergies. Vital signs are within normal limits for her age.

30. The medical helicopter is en route to your facility with a 16-year-old male who was downhill skiing and hit a tree. Bystanders report that he lost control and hit his head. He was intubated at the scene and remains unresponsive.

31. A healthy middle-aged man presents to the emergency department with his left hand wrapped in a bloody cloth. "I was using my table saw, and my hand slipped. I think I lost of couple of fingertips." No past medical history, no medications or allergies. Vital signs are within normal limits. Pain 6/10.

32. A 27-year-old female wants to be checked by a doctor. She has been experiencing low abdominal pain (6/10) for about 4 days. This morning, she began spotting. She denies nausea, vomiting, diarrhea, or urinary symptoms. Her last menstrual period was 7 weeks ago. Past medical history includes previous ectopic pregnancy. Vital signs: T 36.6°C (98°F), HR 66 beats/minute, RR 14 breaths/minute, BP 106/68 mm Hg.

33. "My right leg is swollen, and my calf hurts," reports a 47-year-old morbidly obese female sitting in a motorized scooter. The patient denies chest pain or shortness of breath but admits to a history of type 2 diabetes and hypertension. Vital signs: T 36.6°C (98°F), RR 24 breaths/minute, HR 78 beats/minute, BP 158/82 mm Hg, SpO_2 98%. Pain 6/10.

34. "I think my son has swimmer's ear. He spends half the day in the pool with his friends, so I am not surprised," the mother of a 10-year-old boy tells you. The child has no complaints except painful, itchy ears. Vital signs: T 36.1°C 97°F, HR 88 beats/minute, RR 18 breaths/minute, BP 100/68 mm Hg.

35. An ambulance presents to the ED with a 54-year-old female with chronic renal failure who did not go to dialysis yesterday because she was feeling too weak. She tells you to look in her medical record for a list of her current medications and past medical history. Her vital signs are all within normal limits.

36. A 68-year-old female presents to the ED with her right arm in a sling. She was walking out to the mailbox and slipped on the ice. "I put my arm out to break my fall. I was lucky I didn't hit my head." Right arm with good circulation, sensation, and movement, obvious deformity noted. Past medical history: takes ibuprofen for arthritis. No known drug allergies. Vital signs within normal limits. She rates her pain as 6/10.

37. "I just don't feel right," reports a 21-year-old female who presented to the ED complaining of a rapid heart rate. "I can barely catch my breath, and I have this funny pressure feeling in my chest." HR is 178 beats/minute and regular, RR 32 breaths/minute, BP 82/60 mm Hg. Her skin is cool and diaphoretic.

38. Concerned parents arrive in the ED with their 4-day-old baby girl who is sleeping peacefully in the mother's arms. "I went to change her diaper," reports the father, "and I noticed a little blood on it. Is something wrong with our daughter?" The mother tells you that the baby is nursing well and weighed 7 lbs., 2 oz (3.23 kg) at birth.

39. "I was using my chainsaw without safety goggles, and I think I got some sawdust in my left eye. It hurts and it just won't stop tearing," reports a healthy 36-year-old male. Vital signs are within normal limits.

40. "It hurts so much when I urinate," reports an otherwise healthy 25-year-old. She denies fever, chills, abdominal pain, or vaginal discharge. Vital signs: T 36.8°C (98.2°F), HR 66 beats/minute, RR 14 breaths/minute, BP 114/60 mm Hg.

41. "I was smoking a cigarette and had this coughing fit, and now I feel short of breath," reports a tall, thin 19-year-old man. No past medical history, No meds or allergies, Vital signs: T 36.6°C (98°F), HR 102 beats/minute, RR 36 breaths/minute, BP 128/76 mm Hg, SpO2 92%. Pain 0/10.

42. A 26-year-old female is transported by ambulance to the ED because she experienced the sudden onset of a severe headache that began after she moved her bowels. She is 28 weeks pregnant. Her husband tells you that she is healthy, takes only prenatal vitamins, and has no allergies. The patient is moaning and does not respond to voice. Emergency medical technicians tell you that she vomited about 5 minutes ago.

43. "I think I'm having a stroke," reports an anxious 40-year-old female. "I looked in the mirror this morning, and the corner of my mouth is drooping, and I can't close my left

eye. You have to help me, please." No past medical history, no medications. Vital signs all within normal limits.

44. An 88-year-old female is brought to the ED by ambulance. This morning, she had an episode of slurred speech and weakness of her left arm that lasted about 45 minutes. She has a history of a previous stroke, and she takes an aspirin every day. She is alert and oriented with clear speech and equal hand grasps.

45. "It is like I have my period. I went to the bathroom, and I am bleeding. This is my first pregnancy, and I am scared. Do you think everything is OK?" asks a 26-year-old healthy female. Vital signs: BP 110/80 mm Hg, HR 72 beats/minute, RR 18 breaths/minute, SpO2 99%, T 37°C (98.6°F). She describes the pain as crampy, but rates it as "1" out of 10.

46. A 42-year-old male presents to triage with a chief complaint of "something in his right eye." He was cutting tree limbs and thinks something went into his eye. No past medical history, no allergies, no medications. On exam, his right eye is reddened and tearing. Pain is 4/10.

47. "Our pediatrician told us to bring the baby to the emergency department to see a surgeon and have some tests. Every time I feed him, he vomits, and it just comes flying out," reports the mother of a healthy appearing 3–week-old. "None of my other kids did this." Normal vaginal delivery. Vital signs are within normal limits.

48. "I suddenly started bleeding and passing clots the size of oranges," reports a pale 34-year-old who is 10 days postpartum. "I never did this with my other two pregnancies. Can I lie down before I pass out?" Vital signs: BP 86/40 mm Hg, HR 132 beats/minute, RR 22 breaths/minute, SpO_2 98%.

49. "I have had a cold for a few days, and today I started wheezing. When this happens, I just need one of those breathing treatments," reports a 39-year-old female with a history of asthma. T 36.6.x°C (98°F), RR 22, HR 88, BP 130/80 mm Hg, SpO_2 99%. No medications, no allergies.

50. "I was seen in the ED last night for my fractured wrist. The bone doctor put this cast on and told me to come back if I had any problems. As you can see, my hand is really swollen, and the cast is cutting into my fingers. The pain is just unbearable." Circulation, sensation, and movement are decreased.

51. A 58-year-old male collapsed while shoveling snow. Bystander CPR was started immediately. He was defibrillated once by the paramedics, resulting in the return of a perfusing rhythm. The hypothermic cardiac arrest protocol was initiated prehospital, and he presents with cold normal saline infusing.

52. "My doctor told me to come to the ED. I had a gastric bypass three weeks ago and have been doing fine, but today I started vomiting and having this belly pain." The patient, an obese 33-year- old female, rates her pain as 6/10. Vital signs: BP 126/70 mm Hg, HR 76 beats/minute, RR 14 breaths/minute, T 36.6°C (98°F).

53. "I had a baby 5 weeks ago, and I am just exhausted. I have seen my doctor twice, and he told me I wasn't anemic. I climb the stairs, and I am so short of breath when I get to the top that I have to sit down, and now my ankles are swollen. What do you think is wrong with me?" asks a 23-year-old obese female.

54. "I am so embarrassed!" An 18-year-old tells you that she had unprotected sex last night. "My friend told me to come to the hospital because there is a pill I can take to prevent pregnancy." The patient is healthy, takes no medications, and has no allergies. Vital signs: T 36.1°C (97°F), HR 78, RR 16, BP 118/80.

55. A 76-year-old male requests to see a doctor because his toenails are hard. Upon further questioning, the triage nurse ascertains that the patient is unable to cut his own toenails. He denies any breaks in the skin or signs of infection. He has a history of chronic obstructive pulmonary disease and uses several metered-dose inhalers. His vital signs are normal for his age.

56. An ambulance arrives with a 42-year-old male who called for an ambulance because of dizziness and nausea every time he tries to move. The patient states, "I feel okay when I lie perfectly still, but if I start to sit up, turn over, or move my head, the room starts to spin, and I have to throw up." No past medical history. Vital signs: T 36.2°C (97.2°F), RR 16 breaths/minute, HR 90 beats/minute, BP 130/82 mm Hg, SpO_2 99%. Pain 0/10.

57. The patient is the restrained driver of a truck involved in a high-speed, multicar collision. Her only complaint is right thigh pain. She has a laceration on her left hand and an abrasion on her left knee. Vital signs: BP 110/74 mm Hg, HR 72 beats/minute, RR 16 breaths/minute. No medications, no allergies, no past medical history.

58. "My wife called for an ambulance because my internal defibrillator gave me a shock this morning when I was eating breakfast. Really scared me! I saw my doctor a few days ago, and he changed some of my medications. Could that be why that happened?" The patient has a significant cardiac history and reports taking multiple medications, including amiodarone. Vital signs: T 36.9°C (98.5°F), RR 20 breaths/minute, HR 90 beats/minute, BP 120/80 mm Hg.

59. "Nurse, I have this pressure in my chest that started about an hour ago. I was shoveling that wet snow, and I may

have overdone it," reports an obese 52-year-old male. He tells you his pain is 10 out of 10 and that he is nauseated and short of breath. His skin is cool and clammy. Vital signs: BP 86/50 mm Hg, HR 52 beats/minute and irregular.

60. "My sister has metastatic breast cancer, and her doctor suggested that I bring her in today to have more fluid drained off her lungs." The fluid buildup is making it harder for her to breathe. The patient is a cachectic 42-year-old female on multiple medications. Vital signs: T 37°C (98.6°F), RR 34 breaths/minute, SpO_2 95%, HR 92 beats/minute, BP 114/80 mm Hg.

61. A 58-year-old male presents to the emergency department complaining of left lower-quadrant abdominal pain for three days. He denies nausea, vomiting, or diarrhea. No change in appetite. Past medical history of hypertension. Vital signs: T 37.8°C (100°F), RR 18 breaths/minute, HR 80 beats/minute, BP 140/72 mm Hg, SpO_2 98%. Pain 5/10.

62. "I think he has another ear infection," the mother of an otherwise healthy 2-year-old tells you. "He's pulling on his right ear." The child has a tympanic temperature of 37.8°C (100.2°F) and is trying to grab your stethoscope. He has a history of frequent ear infections and is currently taking no medication. He has a normal appetite and his urine output is normal, according to the mother.

63. "My son needs a physical for camp," an anxious mother tells you. "I called the clinic, but they can't see him for two weeks and camp starts on Monday." Her son, a healthy 9-year- old, will be attending a summer day camp.

64. "Last night I had sex, and we used a condom but it broke. I just don't want to get pregnant," a teary 18-year-old female tells you. Vital signs are within normal limits.

65. "I have a fever and a sore throat. I have finals this week, and I am scared this is strep," reports a 19-year-old college student. She is sitting at triage drinking bottled water. No past medical history. Medications: birth control pills. No allergies to medications. Vital signs: T 38.1°C (100.6°F), HR 88 beats/minute, RR 18 breaths/minute, BP 112/76 mm Hg.

66. "This 84-year-old male passed out in the bathroom," reports the local paramedics. "When we arrived he was in a third-degree heart block with a rate in the 20s and a blood pressure in the 60s. We began externally pacing him at a rate of 60. He is now alert, oriented, and asking to see his wife."

67. A 16-year-old male wearing a swimsuit walks into the ED. He explains that he dove into a pool, and his face struck the bottom. You notice an abrasion on his forehead and nose as he tells you that he needs to see a doctor because of tingling in both hands.

68. A-25-year-old female presented to the emergency department because of moderate lower abdominal pain with a fever and chills. Two days ago, the patient had a therapeutic abortion at a local clinic. The patient reports minimal vaginal bleeding. Vital signs: T 38.2°C (100.8°F), RR 20 breaths/minute, HR 92 beats/minute, BP 118/80 mm Hg, SpO_2 99%. Pain 5/10.

69. An ambulance radios in that they are in route with a 17-year-old with a single gunshot wound to the left chest. On scene the patient was alert, oriented and had a BP of 82 by palpation. Two large-caliber intravenous catheters were immediately inserted. Two minutes prior to arrival in the ED, the patient's HR was 130 beats/minute and BP was 78 by palpation.

70. "I was at a family reunion, and we were playing baseball. One of my nephews hit the ball so hard, and I tried to catch it, missed, and it hit me right in the eye. My vision is fine. It just hurts," reports a 34-year-old healthy female. Vital signs are within normal limits. There are no obvious signs of trauma to the globe, only redness and swelling in the periorbital area. The patient denies loss of consciousness.

71. A 76-year-old male is brought to the ED because of severe abdominal pain. He tells you, "It feels like someone is ripping me apart." The pain began about 30 minutes prior to admission, and he rates the intensity as 20/10. He has hypertension, for which he takes a diuretic. No allergies. The patient is sitting in a wheelchair moaning in pain. His skin is cool and diaphoretic. Vital signs: HR 122 beats/minute, BP 88/68 mm Hg, RR 24 breaths/minute, SpO_2 94%.

72. The patient states that she is 6 weeks post laparoscopic gastric bypass. Two days ago, she began to have abdominal pain with nausea and vomiting of pureed food. She reports a decrease in her fluid intake and not being able to take her supplements because of vomiting. Vital signs: T 36.5°C (97.8°F), RR 20 breaths/minute, HR 90, BP 110/70 mm Hg, SpO_2 99%. Pain 4/10.

73. A 26-year-old female walks into the triage room and tells you she needs to go into detox again. She has been clean for 18 months but started using heroin again 2 weeks ago when her boyfriend broke up with her. She had called several detox centers but was having no luck finding a bed. She denies suicidal or homicidal ideation. She is calm and cooperative.

74. "My throat is on fire," reports a 19-year-old female. It started a couple of days ago, and it just keeps getting worse. Now I can barely swallow, and my friends say my

voice is different. I looked in the mirror, and I have this big swelling on one side of my throat." No past medical history, no medications, no allergies. Vital signs: T 38.7°C (101.6°F), RR 24 breaths/minute, HR 92 beats/minute, BP 122/80 mm Hg, SpO_2 100% on room air.

75. "My doctor told me to come to the ED. He thinks my hand is infected," a 76-year-old female with arthritis, chronic renal failure, and diabetes tells you. She has an open area on the palm of her hand that is red, tender, and swollen. She hands you a list of her medications and reports that she has no allergies. She is afebrile. Vital signs: HR 72 beats/minute, RR 16 breaths/minute, BP 102/60 mm Hg.

76. Police escort a disheveled 23-year-old handcuffed male into the triage area. The police report that the patient had been standing in the middle of traffic on the local highway screaming about the end of the world. The patient claims that he has been sent from Mars as the savior of the world. He refuses to answer questions or allow you to take vital signs.

77. "My dentist can't see me until Monday, and my tooth is killing me. Can't you give me something for the pain?" a healthy 38-year-old male asks the triage nurse. He tells you the pain started yesterday, and he rates his pain as 10/10. No obvious facial swelling is noted. Allergic to penicillin. Vital signs: T 37.7°C (99.8°F), HR 78 beats/minute, RR 16 breaths/minute, BP 128/74 mm Hg.

78. "I have been on antibiotics for 5 days for mastitis. I am continuing to nurse my baby, but I still have pain and tenderness in my right breast. Now there is this new reddened area," a 34-year-old new mother tells you. The patient reports having a fever and chills and just feeling run down. T 39°C (102.2°F), RR 20 breaths/minute, HR 90 beats/minute, BP 122/80 mm Hg, SpO_2 98%. Pain 6/10.

79. A young male walks into triage and tells you that he has been shot. As he rolls up the left leg of his shorts, you notice two wounds. He tells you that he heard three shots. He is alert and responding appropriately to questions. Vital signs: T 36.8°C (98.2°F), HR 78 beats/minute, RR 16 breaths/minute, BP 118/80 mm Hg.

80. An 82-year-old resident of a local assisted living facility called for an ambulance because of excruciating generalized abdominal pain and vomiting that started a few hours ago. The woman is moaning in pain but is able to tell you that she had a heart attack 6 years ago. Vital signs: T 36.6°C (98°F), RR 28 breaths/minute, HR 102 beats/minute, BP 146/80 mm Hg, SpO_2 98%. Pain 10/10.

81. "I should have paid more attention to what I was doing," states a 37-year-old carpenter who presents to the ED with a 3-centimeter laceration to his right thumb. The thumb is wrapped in a clean rag. "I know I need a tetanus shot," he tells you. BP 142/76 mm Hg, RR 16 breaths/minute, T 37°C (98.6°F).

82. "My son woke me up about three hours ago complaining of a right earache. I gave him some acetaminophen, but it didn't help," the 4-year-old's mother tells you. No fever, other vital signs within normal limits for age.

83. "How long am I going to have to wait before I see a doctor?" asks a 27-year-old female with a migraine. The patient is well known to you and your department. She rates her pain as 20/10 and tells you that she has been like this for 2 days. She vomited twice this morning. Past medical history: migraines, no allergies, medications include Fioricet.

84. An ambulance arrives with a 75-year-old male with a self-inflicted 6-centimeter laceration to his neck. Bleeding is currently controlled. With tears in his eyes, the patient tells you that his wife of 56 years died last week. Health: No known drug allergies, baby aspirin per day, BP 136/82 mm Hg, HR 74 beats/minute, RR 18 breaths/minute, SpO_2, 98% on room air.

85. "My mother is just not acting herself," reports the daughter of a 72-year-old female. She is sleeping more than usual and complains that it hurts to pee." Vital signs: T 38.2°C (100.8°F), HR 98 beats/minute, RR 22 breaths/minute, BP 122/80 mm Hg. The patient responds to verbal stimuli but is disoriented to time and place.

86. An ambulance arrives in the ED with a 57-year-old female with multiple sclerosis. She is bedridden, and her family provides care in the home. The family called for an ambulance because her urinary catheter came out this morning. No other complaints. Vital signs are within normal range. Currently on antibiotics for a urinary tract infection.

87. "I got my belly button pierced a month ago and now it hurts so bad," reports a 19-year-old healthy college student who is accompanied by her roommate. They are chatting about plans for the evening. The area is red, tender, and swollen, and pus is oozing from around the site. Vital signs: T 37.8°C (100°F), HR 74 beats/minute, RR 18 breaths/minute, BP 102/70 mm Hg, SpO_2 100%. Pain 8/10.

88. "Why the hell don't you just leave me alone?" yells a 73-year-old disheveled male who was brought to the ED by ambulance. He was found sitting on the curb drinking a bottle of vodka with blood oozing from a 4-centimeter forehead laceration. He is oriented to person, place, and time and has a Glasgow Coma Scale score of 14.

89. "This is so embarrassing," reports a 42-year-old male. "We were having incredible sex, and I heard a crack. Next thing you know, my penis was flaccid, and I noticed some bruising." The pain is "unbelievable," 20/10. No meds, No known drug allergies.

90. "I have this infection in my cuticle," reports a healthy 26-year-old female. "It started hurting 2 days ago, and today I noticed the pus." The patient has a small paronychia on her right second finger. No known drug allergies. T 37.1°C (98.8°F), RR 14 breaths/minute, HR 62 beats/minute, BP 108/70 mm Hg.

91. A 20-year-old male presents to the ED after being tackled while playing football. He has an obvious dislocation of his left shoulder and complains of severe pain, 10/10. Neurovascular status is intact, and vital signs are within normal limits.

92. A 72-year-old female with obvious chronic obstructive pulmonary disease and increased work of breathing is wheeled into triage. Between breaths, she tells you that she "is having a hard time breathing and has had a fever since yesterday." The SpO_2 monitor is alarming, displaying a saturation of 79 percent.

93. A 17-year-old handcuffed male walks into the ED accompanied by the police. The parents called the police because their son was out of control: he was verbally and physically acting out and threatening to kill the family. He is cooperative at triage and answers your questions appropriately. He has no past medical history or allergies and is currently taking no medications. Vital signs are within normal limits.

94. "I think I need a tetanus shot," a 29-year-old female tells you." I stepped on a rusty nail this morning, and I know I haven't had one for years." No past medical history, no known drug allergies, no medications.

95. A 63-year-old cachectic male is brought in from the local nursing home because his feeding tube fell out again. The patient is usually unresponsive. He has been in the nursing home since he suffered a massive stroke about four years ago.

96. A 28-year-old male presents to the ED requesting to be checked. He has a severe shellfish allergy and mistakenly ate a dip that contained shrimp. He immediately felt his throat start to close, so he used his epinephrine auto-injector. He tells you that he feels okay. No wheezes or rash noted. Vital signs: BP 136/84 mm Hg, HR 108 beats/minute, RR 20 breaths/minute, SpO_2 97%, T 37.2°C (97°F).

97. You are trying to triage an 18-month-old whose mother brought him in for vomiting. The toddler is very active and trying to get off his mother's lap. To distract him, the mother hands him a bottle of juice, which he immediately begins sucking on. The child looks well hydrated and is afebrile.

98. "He was running after his brother, fell, and cut his lip on the corner of the coffee table. There was blood everywhere," recalls the mother of a healthy 19-month-old. "He'll never stay still for the doctor." You notice that the baby has a 2-centimeter lip laceration that extends through the vermilion border. Vital signs are within normal limits for age.

99. A 44-year-old female is retching continuously into a large basin as her son wheels her into the triage area. Her son tells you that his diabetic mother has been vomiting for the past 5 hours, and now it is "just this yellow stuff." "She hasn't eaten or taken her insulin," he tells you. No known drug allergies. Vital signs: BP 148/70 mm Hg, HR 126 beats/minute, RR 24 breaths/minute.

100. An ambulance arrives with a 76-year-old male found on the bathroom floor. The family called for an ambulance when they heard a loud crash in the bathroom. The patient was found in his underwear, and the toilet bowl was filled with maroon-colored stool. Vital signs on arrival: BP 70 by palpation, HR 128 beats/minute, RR 40 breaths/minute. His family tells you he has a history of atrial fibrillation and takes a "little blue pill to thin his blood."

Practice Cases: Answers and Discussion

1. **ESI level 5: No resources.** This patient will need an eye exam and will be discharged to home with prescriptions and an appointment to follow up with an ophthalmologist.

2. **ESI level 1: Requires immediate lifesaving intervention.** The patient's respiratory rate, oxygen saturation, and inability to protect her own airway indicate the need for immediate endotracheal intubation.

3. **ESI level 1: Requires immediate lifesaving intervention.** The patient is unresponsive and will require immediate lifesaving interventions to maintain airway, breathing, circulation, and neurological status. Specifically, the patient will require immediate confirmation of endotracheal tube placement.

4. **ESI level 5: No resources.** The patient needs a prescription refill and has no other medical complaints. His blood pressure is controlled with his current medication. If at triage his blood pressure was 188/124 mm Hg and he complained of a headache, then he would meet the criteria for a high-risk situation and be assigned

to ESI level 2. If this patient's blood pressure were elevated but the patient had no complaints, he or she would remain at ESI level 5. The blood pressure would be repeated and would most likely not be treated in the ED or treated with oral medications.

5. **ESI level 3: Two or more resources.** At a minimum, this patient will require a radiograph of his right arm and suturing of his left elbow laceration.

6. **ESI level 2: High-risk situation.** This 32-year-old female with new-onset shortness of breath is on birth control pills. She is a smoker and is exhibiting signs and symptoms of respiratory distress (SpO_2 and respiratory rate.) Based on history and signs and symptoms, a pulmonary embolus, as well as other potential causes for her respiratory distress, must be ruled out.

7. **ESI level 1: Unresponsive.** This 4-year-old continues to be unresponsive. The patient will require immediate lifesaving interventions to address airway, breathing, and circulation.

8. **ESI level 3: Two or more resources.** At a minimum, this child will need a workup for his abdominal pain, which will include labs and a computed tomography scan or ultrasound – two resources.

9. **ESI level 4: One resource.** The laceration will need to be sutured – one resource.

10. **ESI level 4: One resource.** This patient needs a radiograph to rule out a fracture. A splint is not a resource.

11. **ESI level 2: High-risk situation.** This 4-year-old had a witnessed fall with loss of consciousness and presents to the ED with a change in level of consciousness. She needs to be rapidly evaluated and closely monitored.

12. **ESI level 3: Two or more resources.** This patient has a significant medical history, and based on his presentation, he will require two or more resources, which could include labs and intravenous antibiotics.

13. **ESI level 4: One resource.** She will need one resource – labs – which will include a urinalysis and urine culture. She most likely has a urinary tract infection that will be treated with oral medications.

14. **ESI level 2: High-risk situation.** A temperature higher than 38°C (100.4°F) in an infant less than 28 days old is considered a high-risk situation no matter how good the infant looks. Infants in this age range are at a high risk for bacteremia.

15. **ESI level 3: Two or more resources.** At a minimum, she will require labs and intravenous antibiotics.

16. **ESI level 1: Requires immediate lifesaving intervention.** The patient is presenting with signs of shock – hypotension, tachycardia, and tachypnea. Based on the mechanism of injury and presenting vital signs, this patient requires immediate lifesaving interventions, including aggressive fluid resuscitation.

17. **ESI level 3: Two or more resources.** This history is consistent with pneumonia. Because the patient is not in acute respiratory distress, he or she does not meet ESI level-2 criteria. This patient will require labs, a chest radiograph, and perhaps intravenous antibiotics.

18. **ESI level 5: No resources.** This patient will require a physical exam. He has no signs and symptoms of an abscess or cellulitis, so he will be referred to a dentist for treatment. In the emergency department, he may be given medications by mouth. On arrival he rates his pain as 9/10, but because he does not meet the criteria for ESI level 2, he would not be given the last open bed.

19. **ESI level 3: Two or more resources.** Lab studies, intravenous fluid, and an intravenous antiemetic are three of the resources this patient will require. The patient is not high risk or in severe pain or distress.

20. **ESI level 3: Two or more resources.** A patient with a known history of migraines with vomiting will require pain medication, an antiemetic, and fluid replacement. The pain is not severe at 6/10. This patient is not high risk.

21. **ESI level 4: One resource.** This patient will require a laceration repair. A tetanus booster is not a resource.

22. **ESI level 1: Requires immediate lifesaving intervention.** From the history and presentation, this patient appears to have a significant airway injury and will require immediate intubation. Her respiratory rate is 40 breaths/minute, and she is in respiratory distress.

23. **ESI level 3: Two or more resources.** Lab studies, intravenous fluid, and an intravenous antiemetic are three of the resources this patient will require. She is showing signs of dehydration.

24. **ESI level 3: Two or more resources.** At a minimum, she will require labs and noninvasive vascular studies of her lower leg. She should be placed in a wheelchair with her leg elevated and instructed not to walk until the doctor has seen her.

25. **ESI level 2: High-risk situation.** Patients taking warfarin who fall are at high risk of internal bleeding. Although the patients' vital signs are within normal limits and he shows no signs of a head injury, he needs a prompt evaluation and a computed tomography scan of the head.

26. **ESI level 5: No resources.** Following a physical exam, this patient will be sent home with prescriptions and appropriate discharge instructions.

27. **ESI level 2: High-risk situation.** The mechanism of injury is significant, and this patient has the potential for serious injuries. He needs to be evaluated by the trauma team and should be considered high risk. If his BP were 70 by palpation and his HR was 128 beats/minute, he would be rated ESI level 1, requiring immediate lifesaving intervention.

28. **ESI level 5: No resources.** No resources are required. Following a physical exam, this patient will be sent home with appropriate discharge instructions and a prescription if indicated.

29. **ESI level 3: Two or more resources.** An obvious open fracture will necessitate this patient going to the operating room. At a minimum, she will need the following resources: radiograph, labs, intravenous antibiotics, and intravenous pain medication.

30. **ESI level 1: Requires immediate lifesaving interventions.** Prehospital intubation is one of the criteria for ESI level 1. This patient has sustained a major head injury and will require an immediate trauma team evaluation.

31. **ESI level 3: Two or more resources.** Based on the patient's presentation, he will require at least intravenous pain medication and laceration repairs. In addition, he may need a radiograph and intra-venous antibiotics.

32. **ESI level 3: Two or more resources.** Based on her history, this patient will require two or more resources – labs and an ultrasound. She may in fact be pregnant. Ectopic pregnancy is a possibility, but this patient is currently hemodynamically stable, and her pain is generalized across her lower abdomen.

33. **ESI level 3: Two or more resources.** This patient is at high risk for a deep vein thrombosis. For diagnostic purposes, she will require two resources: labs and an ultrasound. If a deep vein thrombosis is confirmed, she will require additional resources – remember, ESI level 3 is two or more resources. If this patient were short of breath or had chest pain, they would meet ESI level-2 criteria.

34. **ESI level 5: No resources.** This child needs a physical exam. Even if eardrops are administered in the emergency department, this does not count as a resource. The family will be sent home with instructions and a prescription.

35. **ESI level 2: High-risk situation.** A complaint of weakness can be due to a variety of conditions, such as anemia or infection. A dialysis patient who misses a treatment is at high risk for hyperkalemia or other fluid and electrolyte problems. This is a patient who cannot wait to be seen and should be given your last open bed.

36. **ESI level 3: Two or more resources.** It looks like this patient has a displaced fracture and will need to have a closed reduction prior to casting or splinting. At a minimum, she needs radiographs and an orthopedic consult. Her vital signs are stable, so there is no need to uptriage her to ESI level 2. Her pain is currently 6/10. If she rated her pain as 9/10 and she is tearful, would you uptriage her to a rating of ESI level 2? Probably not, given the many nursing interventions you could initiate to decrease her pain, such as ice, elevation, and appropriate immobilizations.

37. **ESI level 1: Requires immediate lifesaving interventions.** The patient is hypotensive with a heart rate of 178 beats/minute. She is showing signs of being unstable – shortness of breath and chest pressure. This patient requires immediate lifesaving interventions, which may include medications and cardioversion.

38. **ESI level 5: No resources.** The parents of this 4-day-old need to be reassured that a spot of blood on their baby girl's diaper is not uncommon. The baby is nursing and looks healthy.

39. **ESI level 4: One resource.** This patient will require eye irrigation. Eye drops are not a resource. A slit lamp exam is part of the physical exam of this patient.

40. **ESI level 4: One resource.** This patient will require one resource – labs. A urinalysis and urine culture will be sent and, depending on your institution, a urine pregnancy test. One or all of these tests count as one resource.

41. **ESI level 2: High-risk situation.** This young, healthy male has an elevated respiratory rate and a low oxygen saturation. The patient's history and signs and symptoms are suggestive of a spontaneous pneumothorax. He needs to be rapidly evaluated and closely monitored.

42. **ESI level 1: Requires immediate lifesaving intervention.** From the history, it sounds like this patient has suffered some type of head bleed. She is currently unresponsive to voice and could be showing signs of increased intracranial pressure. She may not be able to protect her own airway and may need to be emergently intubated.

43. **ESI level 2: High-risk situation.** Facial droop is one of the classic signs of a stroke. This patient needs to be evaluated by the stroke team and have a head CT within minutes of arrival in the ED. Many nurses want to make all stroke alerts ESI level 1. This patient does not meet level 1 criteria as she does not require immediate lifesaving

interventions. The triage nurse needs to facilitate moving this patient into the treatment area and initiate the stroke alert process.

44. **ESI level 2: High-risk situation.** The patient's history indicates that she may have had a transient ischemic attack this morning. The patient is high risk, and it would not be safe for her to sit in the waiting room for an extended period of time.

45. **ESI level 3: Two or more resources.** Based on her history, this patient will require two or more resources – labs and an ultrasound. She may be experiencing a spontaneous abortion. Currently, she is hemodynamically stable and has minimal cramping or pain.

46. **ESI level 4: One resource.** The only resource this patient will require is irrigation of his eyes. A slit lamp exam is not considered a resource but is part of the physical exam.

47. **ESI level 3: Two or more resources.** A 3- week-old with projectile vomiting is highly suspicious for pyloric stenosis. The infant will need, at minimum, labs to rule out electrolyte abnormalities, an ultrasound, and a surgery consult.

48. **ESI level 1: Requires immediate lifesaving intervention.** This patient is presenting with signs and symptoms of a post-partum hemorrhage. She tells you she is going to pass out, and her vital signs reflect her fluid volume deficit. The patient needs immediate intravenous access and aggressive fluid resuscitation.

49. **ESI level 4: One resource.** This patient will need a hand-held nebulizer treatment for her wheezing. No labs or radiograph should be necessary because the patient does not have a fever.

50. **ESI level 2: High-risk situation.** The recent application of a cast along with swelling of the hand and unbearable pain justifies an ESI level- 2 acuity rating. He may have compartment syndrome.

51. **ESI level 1: Requires immediate lifesaving intervention.** Studies have shown that lowering brain temperature post cardiac arrest decreases ischemic damage. This patient requires immediate lifesaving interventions to airway, breathing, circulation, and neurologic outcome. Even though the patient converted to a stable rhythm, the nurse should anticipate that additional lifesaving interventions might be necessary.

52. **ESI level 3: Two or more resources.** She will need two or more resources – laboratory tests, intravenous fluid, medication for her nausea, and probably a computed tomography scan of her abdomen. This patient will be in your emergency department for an extended period of time being evaluated. If her pain were 10/10 and she was tachycardic, the patient would meet the ESI level-2 criteria.

53. **ESI level 2: High-risk situation.** This patient is describing more than just the fatigue or anemia. This patient could be describing the classic symptoms of a low-volume but high-risk situation – peripartum cardiomyopathy, a form of cardiomyopathy that occurs in the last month of pregnancy and up to five months post-partum. There is a decrease in the left ventricular ejection fraction which causes congestive heart failure.

54. **ESI level 5: No resources.** This patient will need a bedside pregnancy test before receiving medication. She may be rated an ESI level 4 if your institution routinely sends pregnancy tests to the lab.

55. **ESI level 5: No resources.** This elderly gentleman has such brittle toenails that he is no longer able to clip them himself. He requires a brief exam and an outpatient referral to a podiatrist.

56. **ESI level 3: Two or more resources.** Based on the history, this patient may have acute labyrinthitis and will require two or more resources – intravenous fluids and an intravenous antiemetic.

57. **ESI level 2: High-risk situation.** Based on mechanism of injury, this patient will need rapid evaluation by the trauma team.

58. **ESI level 2: High-risk situation.** This patient is not someone who should sit in your waiting room. He does not meet the criteria for ESI level 1, but he meets the criteria for ESI level 2. The patient's internal defibrillator fired for some reason and needs to be evaluated.

59. **ESI level 1: Requires immediate lifesaving intervention.** The history combined with the signs and symptoms indicate that this patient is probably having a myocardial infarction. The "pressure" started after shoveling wet snow, and now he is nauseated and short of breath, and his skin is cool and clammy. He needs establishment of immediate intravenous access, administration of medications, and placing of external pacing pads.

60. **ESI level 2: High-risk situation.** Breast cancer can metastasize to the lungs and can cause a pleural effusion. The collection of fluid in the pleural space leads to increasing respiratory distress as evidenced by the increased respiratory rate and work of breathing.

61. **ESI level 3: Two or more resources.** Abdominal pain in a 58-year-old male will require two or more resources. At a minimum, he will need labs and an abdominal computed tomography scan.

62. **ESI level 5: No resources.** This child has had previous ear infections and is presenting today with the same type of symptoms. He does not appear ill, and his vital signs are within normal limits. The child requires a physical exam and should be discharged with a prescription.

63. **ESI level 5: No resources.** Because the mother could not get an appointment with a primary care physician, she brought her son to the emergency department for a routine physical exam. He will be examined and discharged.

64. **ESI level 5: No resources.** This patient will need a bedside pregnancy test prior to receiving medication. She may be rated an ESI level 4 if your institution routinely sends pregnancy tests to the lab.

65. **ESI level 4: One resource.** In most EDs, this patient will have a rapid strep screen sent to the lab, one resource. She is able to drink fluids and will be able to swallow pills if indicated.

66. **ESI level 1: Requires immediate lifesaving intervention.** The patient is in third-degree heart block and requires external pacing to preserve airway, breathing, and circulation.

67. **ESI level 2: High-risk situation.** Because of the mechanism of injury and his complaints of tingling in both hands, this patient should be assigned ESI level 2. He has a cervical spine injury until proven otherwise. He is not someone who should be rated an ESI level 1 in that he does not require immediate lifesaving interventions to prevent death. At triage, he needs to be appropriately immobilized.

68. **ESI level 3: Two or more resources.** Based on the history, this patient will require at a minimum labs and intravenous antibiotics. In addition she may need a gynecological consult and intravenous pain medication.

69. **ESI level 1: Requires immediate lifesaving interventions.** The trauma team needs to be in the trauma room and ready to aggressively manage this 17-year-old with a single gunshot wound to the left chest. He will require airway management, fluid resuscitation and, depending on the injury, a chest tube or rapid transport to the operating room.

70. **ESI level 4: One resource.** The history is suggestive of an orbital fracture. The patient will require one resource – a radiograph. She will need a visual acuity check and eye evaluation, but these are not ESI resources.

71. **ESI level 1: Requires immediate lifesaving intervention.** The patient is presenting with signs of shock: hypotensive, tachycardic, with decreased peripheral perfusion. He has a history of hyper-tension and is presenting with signs and symptoms that could be attributed to a dissecting abdominal aortic aneurysm. He needs immediate intra-venous access, aggressive fluid resuscitation, and, perhaps, blood prior to surgery.

72. **ESI level 3: Two or more resources.** Abdominal pain and vomiting post gastric bypass needs to be evaluated. This patient needs labs, intravenous access, antiemetics, and a computed tomography scan.

73. **ESI level 4: One resource.** This patient is seeking help finding a detoxification program that will help her. She is not a danger to herself or others. The social worker or psychiatric counselor should be consulted to assist her. Once a placement has been found, she can be discharged from the emergency department and can get herself to the outpatient program. If your social worker or psychiatric counselor requires a urine toxicology or other lab work, the patient will require two or more resources and then meet ESI level-3 criteria.

74. **ESI level 2: High-risk situation.** Voice changes, fever, difficulty swallowing, and swelling on one side of the throat can be signs of a peritonsillar abscess. The patient needs to be monitored closely for increasing airway compromise and respiratory distress.

75. **ESI level 3: Two or more resources.** This patient has a complex medical history and presented with an infected hand. At a minimum she will need labs, intravenous access, and intravenous antibiotics to address her presenting complaint. Her vital signs are normal, so there is no reason to uptriage her to ESI level 2.

76. **ESI level 2: High-risk situation.** This patient is experiencing delusions and may have a past medical history of schizophrenia or other mental illness, or he may be under the influence of drugs. Regardless, the major concern is patient and staff safety. He needs to be taken to a safe, secure area and monitored closely.

77. **ESI level 5. No resources.** No resources should be necessary. He will require a physical exam, but without signs of an abscess or cellulitis, this patient will be referred to a dentist. In the ED, he may be given oral medications and prescriptions for antibiotics and/or pain medication. He is not at ESI level 2, even though he rates his pain as 10/10. Based on the triage assessment, he would not be given the last open bed.

78. **ESI level 3: Two or more resources.** This patient probably has been on antibiotics for five days for mastitis and now presents to the ED due to fever, chills, and feeling rundown. She will require labs, intravenous antibiotics, a lactation consult if avail-able, and perhaps admission.

79. **ESI level 2: High-risk situation.** This patient has two obvious wounds, but until he is thoroughly examined in the trauma room, you cannot rule out the possibility that he has another gunshot wound. The wounds on his thigh look non-life-threatening, but a bullet could have nicked a blood vessel or other structure; therefore, he meets ESI level-2 criteria. His vital signs are within normal limits, so he does not meet the ESI level-1 criterion.

80. **ESI level 2: High-risk situation with severe pain and distress.** Abdominal pain in the elderly can be indicative of a serious medical condition, and a pain score of 10/10 is significant. The triage nurse needs to keep in mind that due to the normal changes of aging, the elderly patient may present very differently than a younger patient and is more likely to present with vague symptoms.

81. **ESI level 4: One resource.** This patient will require a laceration repair. A tetanus booster is not a resource.

82. **ESI level 5: No resources.** Following a physical exam, this 4-year-old will be sent home with appropriate discharge instructions and perhaps a prescription.

83. **ESI level 3: Two or more resources.** At a minimum, this patient will require intravenous access with fluid, intravenous pain medication, and an antiemetic. Although she rates her pain as 20/10, she should not be assigned to ESI level 2. She has had the pain for two days, and the triage nurse cannot justify giving the last open bed to this patient. The triage nurse will need to address this patient's concerns about wait time.

84. **ESI level 2: High-risk situation.** This 75-year-old male tried to kill himself by cutting his throat. Because of the anatomy of the neck, this type of laceration has the potential to cause airway, breathing, and/or circulation problems. At the same time, he is suicidal, and the ED needs to ensure that he does not leave or attempt to harm himself further.

85. **ESI level 2: New onset confusion, lethargy, or disorientation.** The daughter reports that her mother has a change in level of consciousness. The reason for her change in mental status may be a urinary tract infection that has advanced to bacteremia. She has an acute change in mental status and is therefore high risk.

86. **ESI level 4: One resource.** The patient was brought to the emergency department for a new urinary catheter – one resource. There are no other changes in her condition, and she is already on antibiotics for a urinary tract infection, so no further evaluation is needed.

87. **ESI level 3: Two or more resources.** Based on the history, this patient may have a cellulitis from the navel piercing. At a minimum she will require labs and intravenous antibiotics.

88. **ESI level 2: High-risk situation.** The history of events is unclear. How did the 73- year-old gentleman get the laceration on his forehead? Did he fall? Get hit? Because of his age, presentation, and the presence of alcohol, he is at risk for a number of complications.

89. **ESI level 2: High-risk situation.** This patient may be describing a penile fracture, a medical emergency. It is most often caused by blunt trauma to an erect penis. This patient needs to be evaluated promptly.

90. **ESI level 4: One resource.** This young woman needs an incision and drainage of her paronychia. She will require no other resources.

91. **ESI level 2: Severe pain and distress.** The triage nurse is unable to manage his pain at triage other than applying a sling and ice. He will require intravenous opioids to reduce his pain and enable reduction of his shoulder.

92. **ESI level 1: Requires immediate lifesaving intervention.** Immediate aggressive airway management is what this patient requires. Her saturation is very low, and she appears to be tiring. The triage nurse does not need the other vital signs in order to decide that this patient needs immediate care.

93. **ESI level 2: High-risk situation.** Homicidal ideation is a clear high-risk situation. This patient needs to be placed in a safe, secure environment, even though he is calm and cooperative at triage.

94. **ESI level 5: No resources.** A tetanus immunization does not count as a resource. The patient will be seen by a physician or midlevel provider and receive a tetanus immunization and discharge instructions. This patient will require no resources.

95. **ESI level 4: One resource.** This patient will be sent back to the nursing home after the feeding tube is reinserted. There is no acute change in his medical condition that warrants any further evaluation. He is unresponsive, but that is the patient's baseline mental status, so he is not at ESI level 1.

96. **ESI level 2: High-risk situation for allergic reaction.** The patient has used his epinephrine auto-injector but still requires additional medications and close monitoring.

97. **ESI level 5: No resources.** A physical exam and providing the mother with reassurance and education is what this 18-month-old will require. His activity level is appropriate, and he is taking fluids by mouth.

98. **ESI level 3: Two or more resources.** A laceration through the vermilion border requires the physician to line up the edges exactly. Misalignment can be noticeable. A healthy 19-month-old will probably not cooperate. In most settings, he will require procedural sedation, which counts as two resources. The toddler's vital signs are within normal limits for his age, so there is no reason to uptriage to ESI level 2.

99. **ESI level 2: High-risk situation.** A 44-year-old diabetic with continuous vomiting is at risk for diabetic ketoacidosis. The patient's vital signs are a concern, as her heart rate and respiratory rate are both elevated. It is not safe for this patient to wait for an extended period of time in the waiting room.

100. **ESI level 1: Requires immediate lifesaving intervention.** This 76-year-old patient is in hemorrhagic shock from his gastrointestinal bleed. His blood pressure is 70, his heart rate is 128 beats/minute, and his respiratory rate is 40 breaths/minute, all indicating an attempt to compensate for his blood loss. This patient needs immediate intravenous access and the administration of fluid, blood, and medications.

10

Competency Cases

This chapter can be used to assess competency. It includes two sets of 25 case studies. The cases are divided into two sets to enable flexibility in ESI competency evaluation. For example, Set A can be used for initial assessment and Set B can be used for remedial or follow-up assessment. Both sets contain realistic patient scenarios that a triage nurse would encounter in any emergency department. Please read each case and, based on the information provided, assign a triage acuity rating using ESI. Answers to all cases follow after Set B.

Set A Competency Cases

1. "I think I picked up a bug overseas," reports a 34-year-old male presenting to the emergency department complaining of frequent watery stools and abdominal cramping. "I think I am getting dehydrated." Vital signs: T 36.7°C (98°F), RR 22 breaths/minute, HR 112 beats/minute, BP 120/80 mm Hg, SpO_2 100%. His lips are dry and cracked.

2. "I think he broke it," reports the mother of a 9- year-old boy. "He was climbing a tree and fell about 5 feet (1.5 meters), landing on his arm. I am a nurse, so I put on a splint and applied ice. He has a good pulse." The arm is obviously deformed. Vital signs: T 36.7°C (98°F), RR 26 breaths/minute, HR 90 beats/minute, SpO_2 99%. Pain 5/10.

3. "I don't know what's wrong with my baby girl," cries a young mother. She reports that her 2- week-old baby is not acting right and is not interested in eating. As you begin to undress the baby, you notice that she is listless and her skin is mottled.

4. "My pain medications are not working anymore. Last night I couldn't sleep because the pain was so bad," reports a 47-year-old female with metastatic ovarian cancer. "My husband called my oncologist, and he told me to come to the emergency department." The patient rates her pain as 9/10. Vital signs are within normal limits.

5. A 48-year-old male tells you that he has a history of kidney stones and thinks he has another one. He has right costovertebral angle pain that radiates around to the front and into his groin. He is nauseous but tells you he took a pain pill, and right now he has minimal pain. He denies vomiting. Vital signs: T 36.7°C (98°F), RR 16 breaths/minute, HR 80 beats/minute, BP 136/74 mm Hg, SpO_2 100%. Pain 3/10.

6. "After my pediatrician saw my son's rash, he said I had to bring him to the emergency department immediately. He has this rash on his face and chest that started today. He has little pinpoint purplish spots he called petechiae. My son is a healthy kid who has had a cold for a couple of days and a cough. My pediatrician said he had to be sure nothing bad is going on. What do you think?"

7. "Her grandfather pulled her by the wrist up and over a big puddle. Next thing you know, she is crying and refusing to move her left arm," the mother of a healthy 3–year-old tells you. Vital signs are within normal limits.

8. A 46-year-old asthmatic in significant respiratory distress presents via ambulance. The paramedics report that the patient began wheezing earlier in the day and had been using her inhaler with no relief. On her last admission for asthma, she was intubated. Vital signs: RR 44 breaths/minute, SpO_2 93% on room air, HR 98 beats/minute, BP 154/60 mm Hg. The patient is able to answer your questions about allergies and medications.

9. A 56-year-old male with a recent diagnosis of late-stage non-Hodgkin's lymphoma was brought to the ED from the oncology clinic. He told his oncologist that he had facial and bilateral arm swelling and increasing shortness of breath. The patient also reports that his symptoms are worse if he lies down. Vital signs: BP 146/92 mm Hg, HR 122 beats/minute, RR 38 breaths/minute, SpO_2 98% on room air, temperature normal.

10. An ambulance arrives with a 28-year-old male who was stabbed in the left side of his neck during an altercation. You notice a large hematoma around the wound, and the patient is moaning he cannot breathe. Vital signs: HR 110 beats/minute, RR 36 breaths/minute, SpO_2 89%.

11. An 11-year-old presents to triage with his mother, who reports that her son has had a cough and runny nose for a week. The child is running around the waiting room and asking his mother for a snack. Vital signs are within normal limits.

12. "I don't know what is wrong with my son," reports the worried mother of a normally healthy eight-year-old male. "He's losing weight and acting so cranky. Last night he was up to the bathroom every hour, and he can't seem to get enough to drink." The child is alert and oriented and answers your questions appropriately. Vital signs: T 37°C (98.6°F), RR 30 breaths/minute, HR 98 beats/minute, BP 92/78 mm Hg, SpO_2 98%.

13. "He has had diarrhea for two days, and he just started throwing up this morning. This has been going around the family, and he seems to have it the worst. He has been drinking before today, but now he doesn't want anything to drink," reports the mother of a 19-month-old. The toddler is awake and alert but quiet in the mother's arms, and you notice his lips are dry and cracked. Vital signs: T 36.2°C (99°F), RR 30 breaths/minute, HR 130 beats/minute, SpO_2 100%.

14. An ambulance arrives with an 87-year-old male who slipped on the ice and injured his right hip. His right leg is shortened and externally rotated. The patient's only complaint is hip pain. He rates his pain as 5/10, and his vital signs are within normal limits.

15. "My baby is having a hard time drinking his bottle," reports the young mother of a 3-month-old. The baby is alert and looking around. You notice a large amount of dried mucus around both nares. Vital signs: T 36.7°C (98°F), RR 40 breaths/minute, HR 132 beats/minute, SpO_2 99%.

16. A 72-year-old female is brought in by ambulance from the nearby nursing home. The ambulance personnel report that she has become increasingly confused over the last 24 hours. She is usually awake, alert, and oriented and takes care of her own activities of daily living. At triage she has a temperature of 37.6°C (99.6°F), HR 86 beats/minute, RR 28 breaths/minute, BP 136/72 mm Hg, SpO_2 94% on room air.

17. Melissa, a 4-year-old with a ventriculoperitoneal shunt (to treat hydrocephalus), is brought to the ED by her parents. The mother tells you that she is concerned that the shunt may be blocked because Melissa is not acting right. The child is sleepy but responds to verbal stimuli. When asked what was wrong, she tells you that her head hurts and she is going to throw up. Vital signs: T 37°C (98.6°F), RR 22 breaths/ minute, HR 120 beats/minute, SpO_2 99% on room air, BP 94/76 mm Hg.

18. Paramedics arrive with a 62-year- old male with a history of a myocardial infarction (four years ago) who is complaining of chest pressure that started an hour ago. The field electrocardiogram shows ST-segment elevation in the anterior and lateral leads. Currently, the patient's HR is 106 beats/minute, RR 28 breaths/minute, BP 72/53 mm Hg, SpO_2 is 95% on a non-rebreather mask. His skin is cool and clammy.

19. "I had a knee replacement three months ago. Now look at it!" states a 64-year-old male. The knee is red, swollen, and tender to touch. Vital signs: T 37.2°C (99°F), RR 20 breaths/minute, HR 74 beats/minute, BP 164/74 mm Hg, SpO_2 97%. Pain 6/10.

20. "This is so embarrassing," reports a 29-year-old male." For the last 12 hours, I have had this thing stuck in my rectum. I have tried and tried to get it out with no success. Can someone help me?" The patient denies abdominal pain or tenderness. Vital signs are within normal limits. Pain 4/10.

21. An ambulance arrives with a 67-year-old female who lives alone. The patient called for the ambulance because she was too sick to get herself to the doctor. The patient has had a fever and cough for three days. She reports coughing up thick green phlegm and is concerned that she has pneumonia. She denies shortness of breath. She has past medical history of hypertension. Vital signs: T 38.9°C (102°F), RR 28 breaths/minute, HR 86 beats/minute, BP 140/72 mm Hg, SpO_2 94%.

22. An ambulance arrives with a 14-year-old male who was snowboarding at a nearby ski area, lost control, and ran into a tree. The patient was wearing a ski helmet; is currently aware, alert, and oriented; and is complaining of pain in the left upper quadrant pain and left thigh. His left femur appears to be broken. Vital signs: BP 112/80 mm Hg, HR 86 beats/minute, RR 14 breaths/minute, SpO_2 98%, and temperature is normal.

23. "I woke up this morning, and there was a bat flying around our bedroom. Scared me half to death, and now I am so worried about rabies," an anxious 48-year-old female tells you. "My husband opened the window, and the bat flew out." Past medical history of ovarian cysts, no medicines or allergies, vital signs are within normal limits.

24. The family of a 74-year-old male called for an ambulance when he developed severe mid-abdominal pain. "My husband is not a complainer," reports his wife." The only medication he takes is for high blood pressure." On arrival

at the ED, the patient's HR is 140 beats/minute, RR 28 breaths/minute, SpO_2 94%, BP 72/56 mm Hg.

25. "I woke up this morning, and my eyes were all red and crusty," reports a 29-year-old kindergarten teacher. "I think I got it from the kids at school," she tells you. She denies pain or other visual disturbances. Her vital signs are within normal limits.

Set B Competency Cases

1. "Without the helmet, I would have been really hurt," reports a 19-year-old healthy male who was involved in a bicycle crash. He lost control of his bike when he hit a pothole. He has a 2-centimeter laceration on his arm and pain over his left clavicle. Vital signs: T 36.3°C (97.4°F), RR 18 breaths/minute, HR 62 beats/minute, BP 122/70 mm Hg, SpO_2 100%. Pain 6/10.

2. When asked why she came to the emergency department, the 18-year-old college student begins to cry. She tells the triage nurse that she was sexually assaulted last night at an off- campus party.

3. "I have this skin rash in my crotch. It looks like jock rot. Probably got it from not washing my gym clothes," reports a 19-year-old healthy male. No abnormal vital signs.

4. "The doctor told me to come back this morning and have my boil checked. He lanced it yesterday and packed some stuff in it. He said he just wants to make sure it is healing OK," reports a 54-year-old diabetic male. The patient goes on to tell you that he feels so much better. Vital signs: T 36.7°C (98°F), RR 16 breaths/minute, HR 64 beats/ minute, BP 142/78 mm Hg, SpO_2 98%. Pain 2/10.

5. A 16-year-old high school hockey player collapsed on the ice after being hit in the anterior chest by the puck. The coaching staff began cardiopulmonary resuscitation almost immediately, and he was defibrillated three times with a return of spontaneous circulation. He arrives in the emergency department intubated.

6. "I have been wheezing for a few days, and today I woke up with a fever. My rescue inhaler doesn't seem to be helping," reports a 43-year-old female with a past history of asthma. Vital signs: T 38.6°C (101.4°F), RR 26 breaths/minute, HR 90 beats/minute, BP 138/70 mm Hg, SpO_2 95%.

7. "This sounds really strange. A bug flew into my right ear while I was gardening. I tried to get it out by using a cotton swab. I just don't know what else to do. This buzzing noise is driving me crazy," a 55-year-old female tells you. No previous medical history and vital signs are within normal limits.

8. "This morning, I stepped on a rusty nail, and it went right through my shoe into my foot. I washed it really well. I read on the internet that I need a tetanus shot." No previous medical history, and vital signs are within normal limits.

9. "I was having breakfast with my wife, and all of a sudden I couldn't see out of my right eye. It lasted about five minutes. I'm just scared because I've never had anything like this happen before," reports a 56-year-old male with a history of hypertension and high cholesterol.

10. "I was walking down the street and twisted my ankle as I stepped off the curb. I don't think it's broken, but it hurts so much," reports a 43-year- old female with a history of colitis. Vital signs: T 36.7°C (98°F), HR 72 beats/minute, RR 18 breaths/minute, BP 134/80 mm Hg, SpO_2 100%. Pain 8/10.

11. A 16-year-old female is brought to the emergency department by her mother, who reports that her daughter took more than 30 acetaminophen tablets about 30 minutes before admission. The tearful girl tells you that her boyfriend broke up with her this morning. No previous medical history, and no allergies or medications. Vital signs within normal limits.

12. "My colitis is acting up," reports a 26-year-old female. "It started with an increased number of stools, and now I am cramping a lot. My gastroenterologist told me to come to the emergency department to be evaluated." No other past medical history. Vital signs: T 36.1°C (97°F), RR 18 breaths/ minute, HR 68 beats/minute, BP 112/76 mm Hg, SpO_2 100%. Pain 6/10.

13. "I was so disappointed about not making the varsity soccer team that I punched a wall," reports a 15-year-old healthy male. His hand is swollen and tender to touch. Vital signs: T 36.1°C (97°F), RR 16 breaths/minute, HR 58 beats/minute, BP 106/80mm Hg, SpO_2 100%. Pain 5/10.

14. A 46-year-old female with a history of sickle cell disease presents to the emergency department because of a crisis. She has pain in her lower legs that began 8 hours ago, and the pain medication she is taking is not working. Currently, she rates her pain as 8/10. She has no other medical problems, and her current medications include folate and hydrocodone/acetaminophen. Vital signs are all within normal limits.

15. "I take a blood thinner because I have had clots in my legs," reports a 54-year-old black male. "They told me that medicine would prevent them, but today I have pain and swelling in my lower leg. It started out just being sore, but now I can hardly walk on it." Denies any other complaints. Vital signs within normal limits.

16. A 65-year-old female is brought in by ambulance from the local nursing home for replacement of her percutaneous endoscopic gastrostomy tube. The information from the nursing home states that she had a massive stroke three years ago and is now aphasic. Her mental status is unchanged, and she has “do not resuscitate/do not intubate” orders. Vital signs are within normal limits.

17. A 26-year-old female presents to the ED because she cannot get an appointment with her therapist. She went home for the holidays, and the visit brought back many issues from her childhood. She is unable to sleep and has been drinking more than usual. She admits to thinking about hurting herself but has no plan. She has a history of previous suicide attempts. Vital signs are within normal limits.

18. “I am here on business for a week, and I forgot to pack my blood pressure medication. I haven’t taken it for 2 days. Do you think one of the doctors will write me a prescription?” asks a 58-year-old male. Vital signs: BP 154/88 mm Hg, HR 64 beats/minute, RR 18 breaths/minute, T 36.7°C (98°F), SpO_2 99%.

19. “I fell running for the bus,” reports a 42-year- old female. “Nothing hurts, I just have road burn on both my knees, and I think I need a tetanus booster.” Vital signs within normal limits.

20. An ambulance radios in that they are en route with a 21-year-old with a single gunshot wound to the left chest. Vital sign: BP 78 by palpation, HR 148 beats/minute, RR 36 breaths/minute, and SpO_2 96% on a non-rebreather mask.

21. A 51-year-old presents to triage with redness and swelling of his right hand. He reports being scratched by his cat yesterday. Past medical history of gastroesophageal reflux disease. Vital signs: BP 121/71 mm Hg, HR 118 beats/minute, RR 18 breaths/minute, T 38.8°C (101.8°F), SpO_2 98%. Pain 5/10.

22. An ambulance arrives with a 52-year-old female overdose. The patient took eight 75 mg tabs of bupropion 2 hours ago because her husband left her for another woman, and now she wants to die. She is awake, alert, and oriented.

23. The local police arrive with a 48-year-old male who was arrested last night for public intoxication. He spent the night in jail, and this morning he is restless and has tremors. The patient usually drinks a case of beer a day and has not had a drink since 7 p.m. Vital signs: BP 172/124 mm Hg, HR 122 beats/minute, RR 18 breaths/minute, T 37°C (98.6°F), SpO_2 97% Pain 0/10.

24. A healthy 10-year-old male is brought to the emergency department by his mother, who reports that her son has not moved his bowels for a week. He is complaining of 7/10 generalized abdominal pain, nausea, and lack of appetite. Vital signs: BP 107/66, HR 75, RR 20, T 37°C (98.6°F), SpO_2 99%.

25. An ambulance arrives with a 22-year-old woman with asthma who began wheezing earlier this morning. She is sitting upright on the ambulance stretcher leaning forward with an albuterol nebulizer underway. The patient is diaphoretic, working hard at breathing and unable to answer your questions. Ambulance personnel tell you that they think she is tiring out. Her respiratory rate is 48, SpO_2 is 94%, and she has a prior history of intubations.

Set A Competency Cases – Answers

1. **ESI level 3: Two or more resources.** From the patient’s history, he will require labs and intravenous fluid replacement – two resources.

2. **ESI level 3: Two or more resources.** It looks as though this patient has a displaced fracture and will need a closed reduction prior to casting or splinting. At a minimum, he needs radiographs and an orthopedic consult. This patient may also require procedural sedation. However, there are already two or more resources involved, so it is not necessary to be overly concerned about counting resources beyond two.

3. **ESI level 1: Requires immediate lifesaving intervention**. Possible aggressive fluid resuscitation.

4. **ESI level 2: Severe pain or distress.** This patient needs aggressive pain management with intravenous medications. There is nothing the triage nurse can do to decrease the patient’s pain level. The answer to “Would you give your last open bed to this patient?” should be yes.

5. **ESI level 3: Two or more resources.** The patient is presenting with signs and symptoms of another kidney stone. At a minimum, he will need a urinalysis and computed tomography scan. If his pain increases, he may need intravenous pain medication. At a minimum, two resources are required. If the pain level were 7/10 or greater and the triage nurse could not manage the pain at triage, the patient could meet level-2 criteria

6. **ESI level 2: High-risk.** Rashes are difficult to triage, but the presence of petechiae is always a high-risk situation. Even if the patient looks good, it is important to recognize that petechiae can be a symptom of a life-threatening infection, meningococcemia.

7. **ESI level 4 or 5:** This case is an example of variations in practice around the country. Many emergency

departments would examine the child and then attempt to reduce the dislocation of the radial head without a radiograph. Others may radiograph the child's arm, which is considered one ESI resource. Reduction is not considered a resource.

8. **ESI level 2: High-risk.** An asthmatic with a prior history of intubation is a high-risk situation. This patient is in respiratory distress as evidenced by her respiratory rate, oxygen saturation, and work of breathing. She does not meet the criteria for ESI level 1, "requires immediate lifesaving intervention."

9. **ESI level 2: High-risk.** This patient is demonstrating respiratory distress with his increased respiratory rate and decreased oxygen saturation. Symptoms are caused by compression of the superior vena cava from the tumor. It is difficult for blood to return to the heart, causing edema of the face and arms.

10. **ESI level 1: Requires immediate lifesaving intervention.** Depending on the exact location, penetrating neck trauma can cause significant injury to underlying structures. Based on the presenting vital signs, immediate actions to address airway, breathing, and circulation are required. Intubation might be necessary due to the large neck hematoma, which may expand.

11. **ESI level 5: No resources.** This healthy-sounding 11-year-old will be examined by a physician and then discharged home with appropriate instructions and a prescription if indicated.

12. **ESI level 2: High-risk.** This patient has an elevated respiratory rate and heart rate. The symptoms of polydipsia and polyuria are two classic signs of diabetic ketoacidosis.

13. **ESI level 3: Two or more resources.** This 19-month-old is dehydrated and will require a minimum of two resources: labs and intravenous fluids. In addition the physician may order an intravenous antiemetic.

14. E**SI level 3: Two or more resources.** This patient probably has a fractured hip and will need a radiograph, intravenous pain medication, and an orthopedic consult. If the reason for a fall in the elderly is unclear, the patient should be assigned ESI level 2 to rule out a cardiac or neurological event.

15. **ESI level 5: No resources.** Following a physical exam, this baby will be discharged to home. Prior to leaving, the mother needs to be taught techniques to keep the baby's nares clear of mucus.

16. **ESI level 2: High risk.** An elderly patient with increasing confusion and a fever needs to be evaluated for an infection. Urinary tract infection and pneumonia need to be ruled out. This patient may be septic and requires rapid evaluation and treatment.

17. **ESI level 2: New-onset confusion, lethargy, or disorientation.** The mother of this 4-year-old knows her child and has probably been through this situation before. A child with a ventriculoperitoneal shunt with a change in level of consciousness and a headache is thought to have a blocked shunt until proven otherwise and may be experiencing increased intracranial pressure.

18. **ESI level 1: Requires immediate lifesaving intervention.** This patient is experiencing another cardiac event that requires immediate treatment. His vital signs and skin perfusion are suggestive of cardiogenic shock, and the patient may require fluid resuscitation or vasopressors to treat hypotension.

19. **ESI level 3: Two or more resources.** The patient is presenting with signs and symptoms of an infection. At a minimum, he will require labs, a radiograph, an orthopedic consult, and intravenous antibiotics.

20. **ESI level 3: Two or more resources.** A radiograph is needed to confirm placement in rectum. Then intravenous sedation and analgesia may be used to enable the physician to remove the foreign body in the ED, or the patient may be admitted for surgery. In this situation, two or more resources are required.

21. **ESI level 3: Two or more resources.** This elderly patient may have pneumonia. Labs and a chest radiograph are required, in addition to intravenous antibiotics. If vital signs are outside the accepted parameters, the patient may be considered high-risk and meet ESI level-2 criteria.

22. **ESI level 2: High-risk.** The mechanism of injury represents a high-risk situation. His left upper quadrant pain could be due to a splenic rupture or injury. He may also have a fractured femur, another source of volume loss. This patient's vital signs are stable, so there is no need for immediate lifesaving intervention, but he is at risk for hemorrhagic shock due to volume loss.

23. **ESI level 4: One resource.** It is unknown whether the patient was bitten by the bat since they were sleeping, so postexposure prophylaxis will be initiated. This is one resource – an intramuscular medication.

24. **ESI level 1:** The patient is presenting with signs of shock, hypotension tachycardia, and tachypnea. He has a history of hypertension and is presenting with signs and symptoms that could be suggestive of a dissecting abdominal aortic aneurysm. On arrival at the emergency department, he will require immediate lifesaving interventions such as immediate intravenous access, aggressive fluid resuscitation, and perhaps blood prior to surgery.

25. **ESI level 5: No resources.** Following a physical exam, this patient will be discharged to home with a prescription and appropriate discharge instructions. No resources are required.

Set B Competency Cases – Answers

1. **ESI level 3: Two or more resources.** Based on the mechanism of injury, this patient will require a radiograph of his clavicle and suturing of his arm laceration. In addition, he may need a tetanus booster, but that does not count as a resource. If the mechanism of injury were more significant, the patient could have met ESI level-2 criteria, high-risk. The patient's pain rating is 8/10, but the triage nurse can intervene by applying a sling and providing ice to decrease the pain and swelling.
2. **ESI level 2: Severe pain or distress.** This patient needs to be taken to a safe, quiet room within the emergency department. Her medical, emotional, and legal needs must be addressed in a timely manner.
3. **ESI level 5: No resources.** Following a physical exam, this young man will be discharged to home with a prescription and appropriate discharge instructions.
4. **ESI level 5: No resources.** This patient was instructed to come back to the emergency department for a wound check. He will be examined and discharged to home. No resources are required. A point-of-care finger stick glucose is indicated, but this is not a resource. If the patient came back with a fever or increasing pain and redness, then his ESI level would reflect the additional resources he would require.
5. **ESI level 1: Requires immediate lifesaving interventions.** From the history, it sounds like the hockey player experienced a disruption in the electrical activity in his heart due to the blow to the chest from the hockey puck. He will require immediate lifesaving interventions to address airway, breathing, and circulation. This patient is intubated, which meets criteria for lifesaving interventions.
6. **ESI level 3: Two or more resources.** This patient has a history of asthma that is not responding to her rescue inhaler. In addition, she has a fever. At minimum, she will need two resources: hand-held nebulizer treatments and a chest radiograph.
7. **ESI level 4: One resource.** This patient will need an ear irrigation to flush it out.
8. **ESI level 5: No resources.** This patient will require a physical exam and then a tetanus booster, which is not considered a resource.
9. **ESI level 2: High-risk.** This patient is exhibiting signs of central retinal artery occlusion, which can lead to permanent vision loss. Rapid evaluation is necessary.
10. **ESI level 4: One resource.** To rule out a fracture, this patient will require a radiograph, one resource. The application of a splint and crutch walking instructions are not counted as resources. This patient does not meet the criteria for ESI level 2 for pain because nursing can immediately initiate interventions to address her pain.
11. **ESI level 2: High-risk.** An overdose is a clear high-risk situation. This patient needs to be seen immediately, and interventions to prevent liver damage must be initiated. At the same time she needs to be placed in a safe, secure environment and monitored closely to prevent harm to herself.
12. **ESI level 3: Two or more resources.** The patient is presenting with a colitis flare-up. She will need labs and possibly intravenous medications and a computed tomography scan of the abdomen, especially in light of her presentation with normal vital signs. Two resources.
13. **ESI level 4: One resource.** This young man presents with a mechanism of injury suggestive of a boxer's fracture. A radiograph is indicated to rule out a fracture – one resource.
14. **ESI level 2: High-risk.** Sickle cell disease requires immediate medical attention because of the severity of the patient's pain, which is caused by the sickle cells occluding small and sometimes large blood vessels. Rapid analgesic management will help prevent the crisis from progressing to the point where hospitalization will be unavoidable.
15. **ESI level 3: Two or more resources.** This patient will need lab tests and lower-extremity vascular studies to rule out a deep vein thrombosis.
16. **ESI level 3: Two or more resources.** This patient will need to be seen by surgery or gastroenterology and her percutaneous endoscopic gastrostomy tube reinserted – two resources.
17. **ESI level 2: High-risk situation.** This patient is a danger to herself and needs to be placed in a safe environment with a constant observer.
18. **ESI level 5: No resources.** The patient will need a history and physical exam and then will be discharged to home with a prescription. An oral dose of his blood pressure medication does not count as a resource.
19. **ESI level 5: No resources.** A tetanus booster is not a resource, and neither is cleaning and dressing abrasions.
20. **ESI level 1: Requires immediate lifesaving intervention.** The trauma team needs to be in the trauma

room and ready to aggressively manage this 21-year-old with a single gunshot wound to the left chest. He will require airway management, fluid resuscitation and, depending on the injury, a chest tube or rapid transport to the operating room.

21. **ESI level 3: Two or more resources.** This patient probably has cellulitis of the hand and will require labs and intravenous antibiotics. Starting a saline lock is not a resource, but intravenous antibiotics are a resource.

22. **ESI level 2: High-risk situation.** An overdose is a high-risk situation, and bupropion overdoses are prone to seizures, hallucinations, and irregular heart rhythms. This patient is suicidal and also needs to be monitored closely for safety.

23. **ESI level 2: High-risk situation.** This 48-year-old male is probably showing signs of alcohol withdrawal, a high-risk situation. He is restless, tremulous, and tachycardic. In addition, he is hypertensive. He is not safe to wait in the waiting room and should be given your last open bed.

24. **ESI level 3: Two or more resources.** Abdominal pain, loss of appetite, and nausea in a 10-year-old who has not had a bowel movement in several days is probably due to constipation. He will need two or more resources—labs, maybe a radiograph, maybe a surgery consult, maybe an enema—but at least two resources.

25. **ESI level 1: Requires immediate lifesaving intervention.** This young asthmatic is tiring out and will need immediate lifesaving intervention that will require at a minimum a nurse and physician at the bedside immediately. The decision may be to continue the respiratory treatments and try intravenous steroids, intravenous magnesium, and heliox immediately. She may also require rapid sequence intubation.

A

Frequently Asked Questions and Post-Quiz Materials for Chapters 2–8

This appendix can be used in locally-developed ESI educational programs or on an as-needed basis to address frequently-asked questions (FAQs) about triaging with the ESI. In addition to these FAQs, additional case studies are provided. The case studies illustrate how the concepts discussed in the FAQs are applied to actual triage situations.

Chapter 2

Frequently Asked Questions

1. **Do I have to upgrade the adult patient's triage level if the heart rate is greater than 100?**

   No, but it is a factor to consider when assigning the ESI level.

2. **Do I have to upgrade the patient's triage level if the pain rating is 7/10 or greater?**

   No. Again, this is one factor to consider when assigning the ESI level.

3. **If the patient is chronically confused, should the patient then automatically be categorized as ESI level 2?**

   No, an ESI level 2 is assigned to patients with an acute change in mental status.

4. **When do I need to measure vital signs?**

   For any patient who meets ESI level-3 criteria. While local emergency departments may have protocols regarding when and by whom vital signs are obtained, the triage nurse determines whether or not they may be useful in determining the ESI level for an individual patient.

Post-Quiz Questions and Answers

Questions

Assign an ESI level to each of these patients.

Level Patient

1. _________ A 62-year-old with cardiopulmonary resuscitation in progress.
2. _________ A 53-year-old with 30% body surface area burn.
3. _________ A 22-year-old who needs a work note.
4. _________ A 12-year-old with an earache.
5. _________ A 45-year-old involved in high speed motor vehicle collision, BP 120/60, HR 72, RR 18.
6. _________ An unresponsive 14-year-old. The emergency medical technician tells you he and his friends had been "doing shots."

Answers

1. ESI level 1
2. ESI level 2
3. ESI level 5
4. ESI level 5
5. ESI level 2
6. ESI level 1

Chapter 3

Frequently Asked Questions

1. **Do I have to assign the ESI triage category of 2 for the 25-year-old female patient who rates her pain as 10/10 and is eating potato chips?**

   No. With stable vital signs and no other factors that would meet high-risk criteria, this patient should be assigned ESI level 3. She will most likely need labs and either radiographs, intravenous access, or pain medications, i.e., two or more resources. You would not use your last open bed for her.

2. **Does an 80-year-old female who is chronically confused need to be triaged as ESI level 2?**

   No. The criteria for ESI level 2 are new onset of confusion, lethargy, or disorientation.

3. **Shouldn't the patient with active chest pain be rated an ESI level 1?**

   Not all patients with chest pain meet ESI level-1 criteria. If they are unresponsive, pulseless, apneic, or not breathing, or require immediate lifesaving intervention, they then meet level-1 criteria. A chest pain patient who is pale, diaphoretic, hypotensive, or bradycardic and who will require immediate intravenous access to improve their hemodynamic status is level 1. Stable patients with active chest pain usually meet high-risk criteria and should be categorized ESI level 2 – immediate placement should be facilitated.

Post-Quiz Questions and Answers

Questions

Read each case and determine whether the patient meets the criteria for ESI level 2. Justify your decision.

1. A 40-year-old male presents to triage with vague, midsternal chest discomfort, occurring intermittently for one month. This morning, he reports a similar episode, which has now resolved. Currently complains of mild nausea but feels pretty good. Medical history: smoker. He is alert, with skin warm and dry, and does not appear to be in any distress.
2. A 22-year-old female on college break presents to the triage desk complaining of a sudden onset of feeling very sick, severe sore throat, and feeling "feverish." She is dyspneic and drooling at triage, and her skin is hot to touch.
3. A 68-year-old male is brought in by his wife for sudden onset of left arm weakness, slurred speech, and difficulty walking. Symptoms began two hours prior to arrival. Past medical history: atrial fibrillation. Medications: digoxin. The patient is awake, oriented, and mildly short of breath. Speech is slurred; right-sided facial droop is present. Left upper-extremity weakness noted with 2/5 muscle strength.
4. A 60-year-old male complains of sudden loss of vision in the left eye that morning. Patient denies pain or discomfort. Past medical history: Coronary artery disease, high blood pressure. The patient is slightly anxious but in no distress.
5. A 22-year-old female with 10/10 abdominal pain for two days. Denies nausea, vomiting, diarrhea, or urinary frequency. Her heart rate is 84, and she is eating ice cream.
6. A 70-year-old female with her right arm in a cast is brought to triage by her daughter. The daughter states that her mother fell yesterday and fractured her arm. The patient is complaining of pain. Daughter states, "They put this cast on yesterday, but I think it's too tight." Daughter reports her mother has been very restless at home and thinks her mother is in pain. Patient has a history of Alzheimer's disease. The patient is confused and mumbling (at baseline per daughter); her face is flushed. She is unable to provide verbal description of her complaints. Her right upper extremity is in a short arm cast and digits appear tense, swollen and ecchymotic. Nail beds are pale; capillary refill is delayed. Patient is not wearing a sling.
7. An 8-month-old presents with fever, cough, and vomiting. The baby has vomited twice this morning. No diarrhea. Mother states the baby is usually healthy but has "not been eating well lately." Does not own a thermometer but states the baby is "hot" to the touch and gave acetaminophen two hours prior to arrival. The baby is wrapped in a blanket, eyes open, and appears listless, with skin hot and moist. Fontanel is sunken. Respirations are regular and not labored.
8. A 34-year-old male presents to triage with right lower quadrant pain, 5/10, all day. Pain is associated with loss of appetite, nausea, and vomiting. Past medical history: None. The patient appears to be in moderate discomfort, guarding his abdomen. Skin is warm and dry.
9. A 28-year-old male arrives with friends with a scalp laceration. Patient states he was struck in the head with a baseball bat one hour prior to arrival. Friends state he "passed out for a couple of minutes." Patient complains of headache, neck pain, mild nausea, and emesis x 1. Patient looks pale, but is otherwise alert and oriented to person, place, and time. There is a 5-cm laceration to the scalp near his left ear with bleeding controlled.
10. A 28-year-old male presents with a chief complaint of tearing and irritation to the right eye. He is a construction worker and was drilling concrete. He states, "I feel like there is something in my eye" and reports he "irrigated the eye several times, but it doesn't feel any better." Patient appears in no severe distress; however, he is continually rubbing his eye. Right eye appears red and irritated with excessive tearing.
11. A 40-year-old male is brought in by his son. He is unable to ambulate due to foot and back pain. Patient states he fell approximately 10 feet (3 meters) off of a ladder and is complaining of foot and back pain. States he landed on both feet and had immediate foot and back pain. Denies loss of consciousness/neck pain. No other signs of trauma noted. The patient appears pale, slightly diaphoretic, and in mild distress. He rates his pain 6/10. Patient is sitting upright in a wheelchair.

12. A 12-year-old female is brought to triage by her mother who states her daughter has been weak and vomiting for three days. The child states she "feels thirsty all the time and her head hurts." Vomited once today. Denies fever, abdominal pain, or diarrhea. No significant past medical history. The child is awake, lethargic, and slumped in the chair. Color is pale, skin warm and dry.
13. A 40-year-old male presents to triage with a gradual increase in shortness of breath over the past two days associated with chest pain. Past medical history: colon cancer. He is in moderate respiratory distress, skin warm and dry.
14. A 60-year-old male presents with complaint of dark stools for one month with vague abdominal pain. Past medical history: None. Pulse is tachycardic at a rate of 140 and he has a blood pressure of 80 by palpation. His skin is pale and diaphoretic.
15. An ambulance arrives with a 25-year-old female with sudden onset of significant vaginal bleeding, 9/10 abdominal pain. The patient is 7 months pregnant. BP 92 by palpation, HR 130 beats/minute.

Answers

1. **ESI level 2.** This patient is high-risk, due to history of angina for 1 month. The patient complained of symptoms of acute coronary syndrome earlier in the morning. Smoking is a significant risk factor; however, the patient presentation is concerning enough to be considered high risk. These are symptoms significant for a potential cardiac ischemic event. Acute myocardial infarction is frequently accompanied or preceded by waxing and waning symptoms. An immediate electrocardiogram is necessary.
2. **ESI level 2.** This patient is at high risk for epiglottitis. This is a life-threatening condition characterized by edema of the vocal cords. Onset is rapid, with a high temperature (usually > 38.5°C/101.3°F), lethargy, anorexia, and sore throat. Patients do not have a harsh cough associated with croup, often assume the tripod position, and also have mouth drooling, an ominous sign. Patients may appear exhausted. Epiglottitis is more common in children but may occur in adults, usually age 20 to 40. These patients are at high risk for airway obstruction and need rapid airway access (preferably in the operating room).
3. **ESI level 2.** This patient is presenting with signs of an acute stroke and requires immediate evaluation. If he meets criteria, he may still be in the time window for fibrinolytic or percutaneous vascular intervention.. He is a very high-priority ESI level-2 patient.
4. **ESI level 2.** High risk for central retinal artery occlusion caused by an embolus. This is one of the few true ocular emergencies and can occur in patients with risk factors of coronary artery disease, hypertension, or embolus. Without rapid intervention, irreversible loss of vision can occur in 60 to 90 minutes.
5. **ESI level 3.** Since she is able to eat ice cream, you would not give your last open bed for this patient. She will probably require at least two resources.
6. **ESI level 2.** High risk for compartment syndrome. Despite the patient being a poor historian, the triage nurse should be able to identify some of the signs of possible compartment syndrome: pain, pallor, pulselessness, paresthesia, and paralysis. The patient requires immediate lifesaving intervention: cutting off the cast and further evaluation for potential compartment syndrome.
7. **ESI level 2.** High risk for sepsis or severe dehydration. If the baby were alert and active with good eye contact, similar complaints, and had a fever of 38°C (100.4°F) or greater, the ESI category would be 3. The temperature is not needed to make the assessment that the baby is high risk. The presence of lethargy and a sunken fontanel are indications of severe dehydration.
8. **Initially ESI level 3.** However, the patient could be upgraded to ESI level 2 if vital signs were abnormal, i.e., heart rate greater than 100. Signs of acute appendicitis include mild to severe right lower quadrant pain with loss of appetite, nausea, vomiting, low-grade fever, muscle rigidity, and left lower quadrant pressure that intensifies the right lower quadrant pain. The presence of all these symptoms and tachycardia would indicate a high risk for a surgical emergency.
9. **ESI level 2.** High risk for epidural hematoma. This is a great example of the importance of understanding mechanism of injury. This man was struck with a baseball bat to the head with enough force to cause a witnessed loss of consciousness. Patients with epidural hematomas have a classic transient loss of consciousness before they rapidly deteriorate. Even though this patient looks good now and is alert and oriented at present, he must be immediately placed for further evaluation.
10. **ESI level 2.** High risk for severe alkaline burn. Concrete is an alkaline substance and continues to burn and penetrate the cornea causing severe burns. Alkaline burns are more severe than burns with acid substances and require irrigation with very large amounts of fluids.
11. **ESI level 2.** High risk for lumbar and calcaneus fractures. Again, mechanism of injury is very important to evaluate.

Although he is not unresponsive or lethargic, he needs rapid evaluation and treatment.

12. **ESI level 2.** Lethargy and high risk for severe dehydration from probable diabetic ketoacidosis. It is not normal for a 12-year-old to be slumped over in a chair. Her history of being thirsty and lethargic suggest a strong suspicion for diabetic ketoacidosis. She needs rapid evaluation and rehydration.

13. **ESI level 2.** High risk for a variety of complications associated with cancer, e.g., pleural effusion, congestive heart failure, further malignancy, and pulmonary embolus. A history of cancer can help identify high-risk status.

14. **ESI level 1.** Patient is rated ESI level 1 after consideration of heart rate, skin condition and blood pressure. Tachycardia and hypotension indicate blood loss. The patient needs immediate hemodynamic support.

15. **ESI level 1.** She is at high risk for abruptio placentae and needs an immediate cesarean section to save the fetus. Abruption occurs when the placenta separates from its normal site of implantation. Primary causes include hypertension, trauma, illegal drug use, and a short umbilical cord. Bleeding may be dark red or absent when hidden behind the placenta. Abruption is usually associated with pain of varying intensity.

Chapter 4

Frequently Asked Questions

1. **Why isn't crutch-walking instruction a resource?**

   Though crutch-walking instruction may consume a fair amount of the ED staff members' time, it is often provided to patients who have simple ankle sprains. These patients are typically classified as ESI level 4 (ankle radiograph = one resource). The patients are clearly less acute and less resource intensive than more complex patients such as those with tibia/fibula fractures who are usually ESI level 3 (leg films, orthopedic consult, cast/splint, intravenous pain medications = two or more resources). A better way to reflect the ED staff's efforts for crutch walking instruction is with a nursing resource intensity measure.

2. **Why isn't a splint a resource?**

   The application of simple, pre-formed splints (such as splints for ankle sprains) is not considered a resource. In contrast, the creation and application of splints by ED staff, such as thumb spica splints for thumb fractures, does constitute a resource. A helpful way to differentiate patients with extremity trauma is as follows: patients with likely fractures should be rated ESI level 3 (two or more resources: radiograph, pain medications, creation and application of splints/casts); whereas patients more likely to have simple sprains can be rated as ESI level 4.

3. **Why isn't a saline or heparin lock a resource?**

   Generally speaking, insertion of a saline lock does not consume a large amount of ED staff time. However, many patients who have saline locks inserted also have at least two other resources (e.g., laboratory tests, intravenous medications) and are therefore classified as ESI level 3 anyway.

4. **Are all moderate sedation patients ESI level 3 or higher?**

   Yes, moderate sedation is considered a complex procedure (two resources) and is generally performed with patients who also have laboratory tests or radiographs and other procedures such as fracture reduction or dilation and curettage.

5. **Which of the following are considered resources: eye irrigation, nebulized medication administration, and blood transfusions?**

   All three are considered resources for the purposes of ESI triage ratings. The resources tend to be used for more acute patients, require significant ED staff time, and likely lead to longer length of stay for patients.

6. **Are all asthmatics ESI level 4 because they will require a nebulized medication?**

   No. Stable asthmatics who only require nebulized medications are assigned ESI level 4. However, some asthmatics are in severe respiratory distress and meet ESI level-2 criteria. Others are somewhere in between and will require intravenous steroids or a radiograph in addition to nebulized treatments and would be assigned ESI level 3. Finally, asthmatics who require only a prescription refill of their inhaler are assigned ESI level 5. They do not require any resources.

Post-Quiz Questions and Answers

Questions

Read the following statements and provide the correct answer.

1. A magnetic resonance imaging procedure is considered a resource in the ESI triage system.

   (True or False)

2. A psychiatry consult is considered a resource in the ESI triage system.

   (True or False)

3. Cardiac monitoring is considered a resource in the ESI triage system.

   (True or False)

4. How many ESI resources will this patient need?

   A healthy 25-year-old construction worker presents with back pain. The triage nurse predicts he will need a lumbar spine radiograph, oral pain medication administered in the ED, and a prescription to take home.

   (0, 1, 2 or more)

5. It is necessary to take vital signs to determine the number of ESI resources an adult ED patient will need.

   (True or False)

6. The triage nurse must have enough experience to be certain about the resources needed for each patient in order to accurately assign an ESI triage level.

   (True or False)

7. A 30-year-old sexually active female patient presents with vaginal bleeding and cramping, does not use birth control, and is dizzy and pale. In determining this patient's ESI triage level, does it matter whether the local ED does urine pregnancy tests at the point of care versus sending a specimen to the laboratory?

   (Yes or No)

   How many resources will this patient require?

   (0, 1, 2 or more)

8. How many ESI resources will this patient need?

   A healthy 40-year-old man presents to triage at 2:00 a.m. with a complaint of a toothache for two days, no fever, and no history of chronic medical conditions.

   (0, 1, 2 or more, irrelevant)

9. How many ESI resources will this patient need?

   A 22-year-old female involved in a high-speed rollover motor vehicle collision and thrown from the vehicle, presents intubated, with no response to pain, and hypotensive.

   (0, 1, 2 or more, irrelevant)

10. How many ESI resources will this patient need?

    A 60-year-old healthy male who everted his ankle on the golf course presents with moderate swelling and pain upon palpation of the lateral malleolus.

    (0, 1, 2 or more, irrelevant)

11. Is it considered an ESI resource if a patient requires a constant observer to prevent a fall?

    (Y/N)

Answers

1. **True.** The magnetic resonance imaging will make use of personnel outside the ED (MRI staff) and increase the patient's ED length of stay.
2. **True.** The consult involves personnel outside the ED (psychiatry team) and increases the patient's ED length of stay.
3. **False.** Monitoring is part of the routine care provided by ED staff. However, most patients who receive monitoring also need at least two other ED resources (electrocardiogram, blood tests, radiographs), and may therefore be classified as ESI level 3.
4. **One ESI resource.** The radiograph is considered a resource since it utilizes personnel outside the ED. The oral pain medication and take-home prescription are not considered resources since they are quick interventions performed by ED personnel.
5. **False.** While vital signs are helpful in uptriage of level-3 patients to level 2, they are not necessary for differentiating patients needing one, two, or more than two resources.
6. **False.** The ESI is based upon the experienced ED triage nurse's prediction, or estimation, of the number and type of resources each patient will need in the ED. The purpose of resource prediction is not to order tests or make an accurate diagnosis, but to quickly sort patients into distinct categories using acuity and expected resources as a guide.
7. **No, it does not matter.** The patient will need at least two resources and be classified as at least a level 3 (depending on vital signs) whether the pregnancy test is done in the ED (not a resource) or in the laboratory (a resource). The predicted resources will include the following: complete blood count, intravenous fluids, ultrasound, and possibly a gynecology consult and intravenous medications if it is determined that she is aborting a pregnancy and the cervical os is open.
8. **No resources.** This patient will likely have a brief exam (not a resource) and receive a prescription for pain medication (not a resource) by the provider, and therefore is an ESI level-5 patient.
9. **Irrelevant.** The patient is an ESI level 1 based on being intubated and unresponsive. The nurse does not need determine the number of resources in order to make the triage classification.

10. **One resource.** The patient will need an ankle radiograph (one resource) and may get an elastic wrap or ankle splint (not a resource) and crutches (not a resource). Simple ankle sprains are generally classified as ESI level 4. However, if the patient were in severe pain that required pain medication by injection, or if he had a deformity that might need a cast, orthopedic consult and/or surgery, then he would need two or more resources and be classified as an ESI level 3.

11. **Yes.** A constant observer at the bedside is considered a resource. However, if a patient is ESI level 2 or high risk because they are a danger to themselves or others, it is not necessary to predict the number of resources they will require in the ED.

Chapter 5

Frequently Asked Questions

1. **Why aren't vital signs required to triage ESI level-1 and level-2 patients?**

   Vital signs are not necessary to rate patients as life threatening (ESI level 1) or high-risk (ESI level 2). Since ESI level-1 and level-2 patients are critical, they require the medical team to respond quickly. Simultaneous actions can occur, and vital signs can be collected as part of the initial assessment in the main acute area of the emergency department. There is one situation in which vital signs are taken for level-1 or level-2 patients. If the life-threatening situation is not initially obvious, the triage nurse may recognize it only when vital signs are taken. For example, a young healthy patient with warm dry skin who complains of feeling dizzy may not initially meet the level-1 or level-2 criteria until the heart rate is obtained and found to be 166.

2. **Why aren't vital signs required for ESI level-4 and level-5 patients?**

   Vital signs are not necessary to rate patients as low or no resource (ESI level 4 or 5). Also, the pain, anxiety, and discomfort associated with an emergency department visit often alter a patient's vital signs. Vital signs may quickly return to normal once the initial assessment is addressed. However, a nurse may choose to assess vital signs if other concerning signs exist (e.g., changes in skin color or mentation, dizziness, sweating). If there is no physical sign indicating a need for vital signs, the patient can be taken into the main emergency department or express care room.

3. **Why are vital signs done on ESI level-3 patients?**

   Vital signs can aid in differentiating patients needing multiple resources as either stable (ESI level 3) or potentially unstable or high-risk (ESI level 2). On occasion, ESI level-3 patients may actually have unstable vital signs while appearing stable. Vital signs for ESI level-3 patients provide a safety check. In general, ESI level-3 patients are more complicated and many are admitted to the hospital. Since these patients are not appropriate for the fast-track area, they are sometimes asked to wait for more definitive care. These patients present a unique challenge to the triaging process, and caregivers find it necessary to rely on vital signs to confirm that an appropriate ESI level has been assigned.

4. **Why are temperatures always done for pediatric patients less than 36 months?**

   Temperature is useful in differentiating pediatric patients that are low or no resource (ESI level 4 or 5) from those that will consume multiple resources. An abnormal temperature in the less than 3-month-old may indicate bacteremia and place the child in a high-risk category.

5. **Why does the literature present conflicting information on the value of vital signs during the triage process?**

   There is no definitive research on the utility of vital signs for emergency department triage. Many factors influence the accuracy of vital sign data. Vital signs are a somewhat operator-dependent component of a patient's assessment. In some cases, vital signs may be affected by many factors such as chronic drug therapy (e.g., beta-blockers). Vital signs may also be used to fulfill part of the public health obligation assumed by emergency departments. And, lastly, vital signs help segment young pediatric patients into various categories.

6. **Does The Joint Commission require vital signs to be done during triage?**

   The Joint Commission does not specifically state a standard for obtaining vital signs. The organization does assert that physiologic parameters should be assessed as determined by patient condition.

7. **Should vital sign criteria be strict in the danger zone vital sign box?**

   In common usage, when the danger zone vital sign criteria are exceeded, uptriage is "considered" rather than automatic. The experienced triage nurse is called on to use good clinical judgment in rating the patient's ESI level. The nurse incorporates information about the vital signs, history, medications, and clinical presentation of the patient in that decision-making process. Research is still needed to determine the predictive value of vital signs at triage and to determine absolute cutoffs for uptriage.

8. **What if ESI level-4 or -5 patients have danger zone vital signs?**

   Though it is not required to take vital signs in order to assign ESI 4 or 5 levels, many patients may have vitals assessed at triage if that is part of the particular ED's operational process. Per the ESI triage algorithm, the triage nurse does not have to take the vital signs into account in determining whether the patient meets ESI level-5 (no resources) or ESI level-4 (one resource) criteria. However, in practice, the prudent nurse will use good clinical judgment and take the vital sign information into account in rating the ESI level. If the patient requests only a prescription refill and has no acute complaints but has a heart rate of 104 beats/minute after walking up the hill to the ED, the nurse might still rate the patient as an ESI level 5. However, if the patient requests a prescription refill and has a heart rate that is irregular at 148 beats/minute, the nurse should rate the patient as ESI level 2. The triage nurse must also consider the following dilemma: an elevated blood pressure in an ESI level-4 or -5 patient. If the patient is asymptomatic related to the blood pressure, the triage level should not change. Most likely, elevated blood pressure in the asymptomatic patient will not be treated in the ED. However, it may be important to refer the patient to a primary care physician for blood pressure follow-up and long-term diagnosis and treatment.

Post-Quiz Questions and Answers

Questions

Rate the ESI level for each of the following patients.

1. A 3-week-old male

   Vital signs:

   Temperature: 38.2° C (100.8° F)

   Heart rate: 160 beats/minute

   Respiratory rate: 48 breaths/minute

   Oxygen saturation: 96%

   Narrative:

   Poor feeding

   Less active than usual

   Sleeping most of the day

2. A 22-month-old, fever, pulling ears, immunizations up to date, history of frequent ear infections

   Vital signs:

   Temperature: 39° C (102.2° F)

   Heart rate: 128 beats/minute

   Respiratory rate: 28 breaths/minute

   Oxygen saturation: 97%

   Narrative:

   Awoke screaming

   Pulling at ears

   Runny nose this week

   Alert, tired, flushed, falling asleep now

   Calm in mom's arms, cries with exam

3. A 6-year-old with cough

   Vital signs:

   Temperature: 40.2° C (104.4° F)

   Heart rate: 140 beats/minute

   Respiratory rate: 30 breaths/minute

   Oxygen saturation: 91%

   Narrative:

   Cough with fever for two days

   Chills

   Short of breath with exertion

   Green phlegm

   Sleeping a lot

4. A 94-year-old male, abdominal pain

   Vital signs:

   Temperature: 37.2° C (98.9° F)

   Heart rate: 100 beats/minute

   Blood pressure: 130/80 breaths/minute

   Oxygen saturation: 93%

   Narrative:

   Vomiting

   Epigastric pain

   Looks sick

5. A 61-year-old female, referred with asthma

   Vital signs:

   Temperature: 37.3° C (99.1° F)

   Heart rate: 112 beats/minute

   Respiratory rate: 28 breaths/minute

   Blood pressure: 157/94

Oxygen saturation: 91%

Peak expiratory flow rate = 200

Narrative:

Asthma exacerbation with dry cough

Steroid dependent

Multiple hospitalizations

Never intubated

6. A 9-year-old male, head trauma

Narrative:

Collided with another player at lacrosse game

Loss of consciousness for "about 5 minutes," witnessed by coach

Now awake with headache and nausea

Answers

1. **ESI level 2.** An infant less than 28 days with a temperature greater than 38°C (100.4° F) is considered high risk regardless of how good they look. For a child between 3 and 36 months with a fever greater than 39°C (102.2° F), the triage nurse should consider assigning ESI level 3 if there is no obvious source for a fever or the child has incomplete immunizations.

2. **ESI level 5.** A child under 36 months of age requires the obtaining of vital signs. This child has a history of frequent ear infections, is up to date on immunizations, and presents with signs of another ear infection. This child meets the criteria for ESI level 5 (exam, oral medication administration, and discharge to home). Danger zone vitals are not exceeded. If the child were underimmunized or there were no obvious source of infection, the child would be assigned to ESI level 3.

3. **ESI level 2.** The clinical picture indicates high probability of tests that equal two or more resources (ESI level 3). Danger zone vital signs exceeded criteria (SpO_2 = 91%, respiratory rate = 30 breaths/minute), making the patient an ESI level 2.

4. **ESI level 2.** The clinical picture mandates ESI level 3 with expected utilization of radiograph, blood work, and specialist consultation resources. Danger zone vital signs are not exceeded. If an experienced triage nurse reported this patient to be in imminent danger of deterioration, the patient may be upgraded to an ESI level 2. A 94-year-old ill-appearing patient presenting with epigastric pain, vomiting, and probable dehydration should be considered a high-risk ESI level-2 patient. If this patient did not look toxic, a rating of ESI level 3 might be an appropriate starting point in the decision algorithm.

5. **ESI level 2.** The clinical picture mandates ESI level 3 with expected utilization of radiograph, blood work, and specialist consultation resources. Respiratory rate and heart rate danger zone vital signs are exceeded, so the patient is uptriaged to ESI level 2.

6. **ESI level 2.** This patient is assigned an ESI level 2 due to the high-risk information provided in the scenario. Vital signs are not necessary, and the patient should be taken to a treatment area immediately for rapid assessment.

Chapter 6

Frequently Asked Questions

1. **How do you rate the ESI level for children with rashes, since some rashes are of great concern while others are less serious?**

   In triaging patients with rashes (as with other conditions), the most important action by the triage nurse is to perform a quick assessment of the patient's appearance, work of breathing, and circulation. These will give the nurse information about the physiological stability of the child and facilitate assessment of their need for life support or their high-risk status. If the child with a rash does not meet level-1 or -2 criteria, then the history becomes an important factor in determining the ESI level. Key information in the history of patients with a rash includes the presence of a fever, exposure to tick bites, or exposure to plants that might indicate contact dermatitis.

2. **Why isn't the placement of a saline lock a resource for pediatric patients? It is a much more intensive procedure in children, especially infants and small children who need to be immobilized for the procedure.**

   While the placement of a saline lock in a young child is a more involved procedure than in adults, in the ESI system resources are proxies for acuity and are not used to monitor nursing resource intensity. Children in need of saline locks are likely going to need other interventions such as laboratory studies and medications or fluid, and thus qualify for ESI level 3 based on these additional resource needs. In the unusual case of a child needing a prophylactic saline lock but no other resources, the child is likely to be of lower acuity and thus not likely to be a level-3 patient.

3. **Since resource prediction is a major part of the ESI, have you considered changing the ESI for pediatrics to reflect the fact that resources for children are different than adults?**

This was reviewed in the course of the pediatric ESI study (Travers et al., 2009). The study results did not support this. The use of resources in the differentiation of ESI levels 3, 4 and 5 is a proxy for acuity, not a staff workload index. Children who require fewer resources tend to be less acute than those who require more resources, even though some resources (e.g., placing a splint) may be more time consuming in children than adults.

4. **Are you going to create a separate pediatric version of the ESI?**

   No. Again, this was researched in the course of the pediatric ESI study (Travers et al., 2009) but the results did not support the creation of a separate ESI for children. An additional consideration is the increased complexity that would be introduced for triage nurses if they had to use two different algorithms, one for children and one for adults. The ESI version 4 does include vital signs criteria for all ages, including three categories for ages from birth to 8 years. It is an all-age triage tool.

Post-Quiz Questions and Answers

Questions

Rate the ESI level for each patient.

Level Patient

1. _________ A 14-year-old with rash on feet, was exposed to poison ivy three days ago. Ambulatory, with stable vital signs.

2. _________ A 3-month-old with petechial and purpuric lesions all over. Vital signs: respiratory rate 60 breaths/minute, heart rate 196 beats/minute, oxygen saturation 90%, temperature 39°C rectal.

3. _________ A 5-year-old with rash on neck and face, with swelling and moist lesions around the eyes and cheeks. Vital signs: respiratory rate 20 breaths/minute, heart rate 100 beats/minute, oxygen saturation 99%, temperature 37°C (98.6 °F). Respirations nonlabored. Was treated by her pediatrician yesterday for poison ivy on the neck, but the rash is worse and spreading today. Mom states child not eating or drinking well today and was up most of the night crying with itching and pain.

4. _________ A 10-year-old presents with facial swelling after eating a cookie at school. Fine red rash all over. Has a history of peanut allergies. Wheezing heard upon auscultation. Vital signs: respiratory rate 16 breaths/minute, heart rate 76 beats/ minute, oxygen saturation 97%, temperature 36.7°C (98.1°F).

5. _________ An 8-year-old healthy child with a fever of 38.7°C (101.6°F) at home arrives at triage with complaints of a sore throat and a fine red sandpaper rash across chest. Sibling at home had a positive strep culture at the pediatrician a few days ago. Respirations are unlabored. Vital signs are stable.

Answers

1. **ESI level 5.** This patient has a rash but is able to ambulate and has no abnormalities in appearance, work of breathing or circulation. During his ED visit he will receive an exam, and perhaps a prescription, but no ESI resources.

2. **ESI level 1.** The baby has the classic signs of meningococcemia with abnormalities in appearance, work of breathing and circulation. She needs immediate lifesaving interventions.

3. **ESI level 3.** Unlike the first patient with poison ivy, this patient will likely need additional interventions including possible intravenous hydration and medications to reduce swelling.

4. **ESI level 2.** Though this patient has stable vital signs, she is at high risk of respiratory compromise given her history and wheezing. She is a high-risk patient and should be promptly taken to the treatment area for monitoring and treatment.

5. **ESI level 4.** This is a healthy patient with stable vital signs and a family member with a positive strep culture. The only resource likely needed is a strep culture.

Chapter 7

Post-Quiz Questions and Answers

Questions

1. Identify the three phases of change described by Lewin.

2. The ESI algorithm is so simple; why do the nurses need two hours or more of education to learn to use it?

3. As the nurse manager of a low-volume emergency department, do I still need an implementation team?

Answers

6. Unfreezing, movement, and refreezing.

7. Yes, the algorithm looks simple, but staff need to develop a clear understanding of each of the decision points. Application to realistic cases will reinforce learning.

8. The change process is never easy. An implementation team provides input from various members of the department. The team can assist in developing and carrying out the implementation plan.

Chapter 8

Frequently Asked Questions

1. **What do we do if we do not have good electronic data monitoring systems for quality improvement efforts?**

   Although it is very helpful and will expand the number of indicators you can monitor, you do not have to have electronic data monitoring to perform ESI quality improvement.

2. **Can staff nurses monitor each other for the accuracy of the ESI triage acuity rating?**

   No. A nurse who is experienced with and expert in triage should determine whether the acuity ratings are correct.

3. **How many indicators should we be monitoring?**

   This is a decision to be made by the leadership team. Select only those indicators that have been identified as important to your ED and select only the number of indicators you have the resources to monitor.

Reference

Travers, D. A., Waller, A., Katznelson, J., & Agans, R. (2009). Reliability and validity of the Emergency Severity Index for pediatric triage. *Academic Emergency Medicine, 16*(9), 843–849. https://doi.org/10.1111/j.1553-2712.2009.00494.x

B

ESI Triage Algorithm, v4

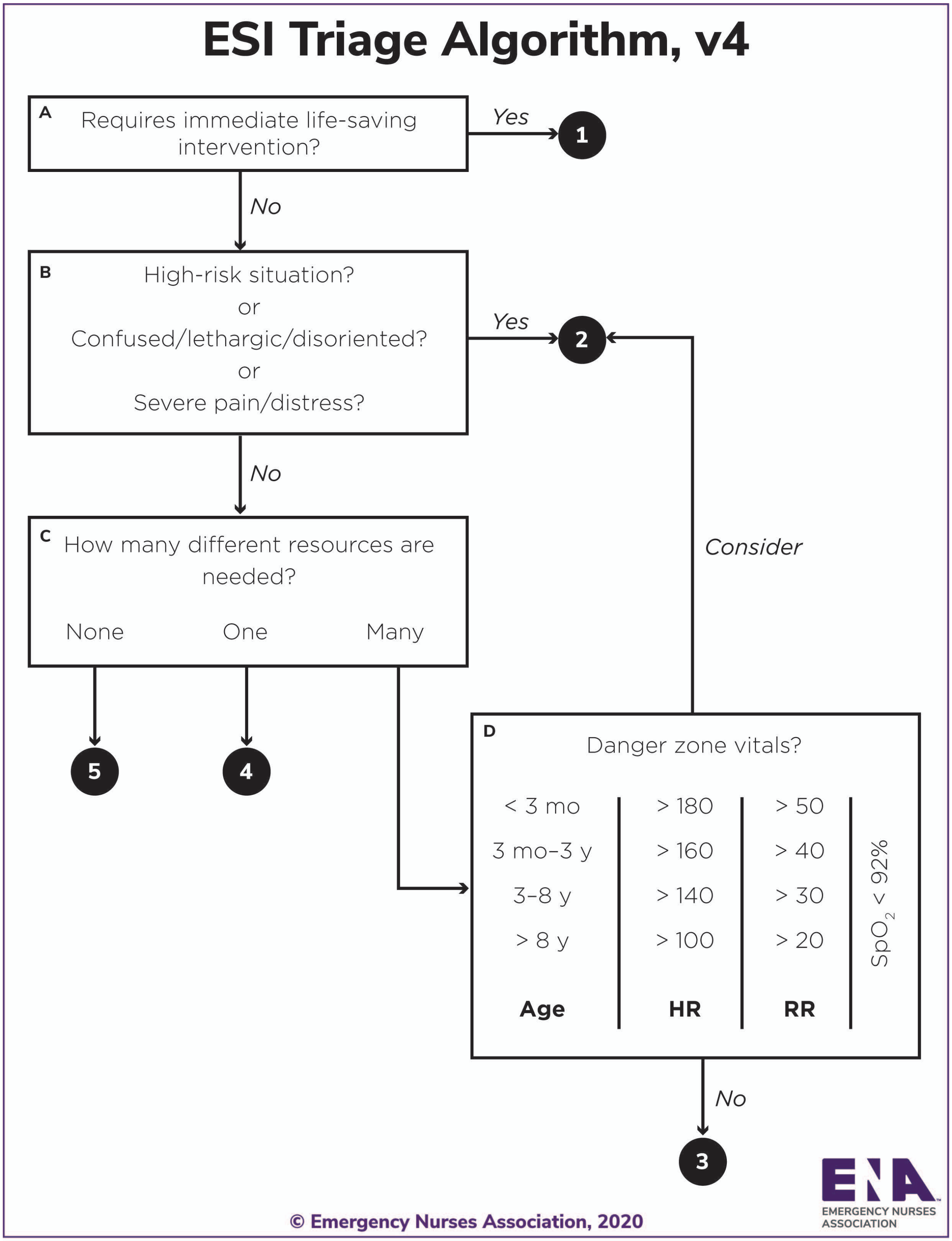

A. Immediate life-saving intervention required: Airway, emergency medications, or other hemodynamic interventions (intravenous access, supplemental oxygen, monitor, electrocardiogram, or labs DO NOT COUNT); and/or any of the following clinical conditions: intubated, apneic, pulseless, severe respiratory distress, pulse oximetry (SpO_2) < 90%, acute mental status changes, or unresponsive.

Unresponsiveness is defined as a patient who is either:

1. Nonverbal and not following commands (acuteley); or
2. Requires noxious stimulus (P or U on AVPU)

B. A high-risk situation is a patient you would put in your last open bed.

Severe pain/distress is determined by clinical observation and/or patient rating of pain greater than or equal to 7 on a 0–10 pain scale.

C. Resources: Count the number of different types of resources, not the individual items (e.g., complete blood count, electrolytes, and coagulants equals one resource; complete blood count plus chest radiograph equals two resources.)

Resources

- Labs (blood, urine)
- Imaging
- Intravenous fluids (hydration)
- Intravenous, intramuscular, or nebulized medications
- Specialty consultation
- Simple procedure = 1 (laceration repair, foley catheter)
- Complex procedure = 2 (procedural sedation)

Not resources

- History and physical exam (including pelvic)
- Point-of-care testing
- Saline or heparin lock
- Oral medications
- Tetanus immunizations
- Prescription refills
- Contact with primary care physician
- Simple wound care (dressings, recheck)
- Crutches, splints, slings

D. Danger zone vitals: Consider uptriage to ESI 2 if any vital sign criterion is exceeded.

Pediatric fever considerations:
1–28 days of age: Assign at least ESI 2 if temperature > 38˚C (100.4˚F)
1–3 months: Consider assigning ESI 2 if temperature > 38˚C (100.4˚F)
3 months–3 years: Consider assigning ESI 3 if:

1. Temperature > 39˚C (101.2˚F); or
2. Incomplete immunizations; or
3. No obvious source of fever

© Emergency Nurses Association, 2020

ENA EMERGENCY NURSES ASSOCIATION

C
Initialisms, Abbreviations, and Acronyms

AAA – abdominal aortic aneurysm

ACEP – American College of Emergency Physicians

ACS – acute coronary syndrome

ATS – Australasian Triage Scale

AVPU – A scale describing level of responsiveness. Is the patient *A*lert? Do they only respond to *V*erbal stimuli? Do they only respond to *P*ainful stimuli? Are they *U*nresponsive?

BP – blood pressure

CNS – Clinical Nurse Specialist

CPR – cardiopulmonary resuscitation

CTAS – Canadian Triage and Acuity Scale

ED – emergency department

ENA – Emergency Nurses Association

ESI – Emergency Severity Index

FAQ – frequently asked questions

HR – heart rate

IOM – Institute of Medicine

PAT – pediatric assessment triangle

QI – quality improvement

RR – respiratory rate

SIRS – systemic inflammatory response syndrome

SpO_2 – oxygen saturation by pulse oximetry

T – temperature

U.S. – United States

VS – vital signs

Index

Note: Page numbers followed by *f* and *t* denote figures and tables, respectively.

A

AAA. *See* abdominal aortic aneurysm (AAA)
abdominal aortic aneurysm (AAA), 18, 52, 97
ACEP. *See* American College of Emergency Physicians (ACEP)
American College of Emergency Physicians (ACEP), 1, 22, 34, 35, 42, 50, 97
ACS. *See* acute coronary syndrome (ACS)
acute coronary syndrome (ACS), 12, 15, 19, 97
ATS. *See* Australasian Triage Scale (ATS)
Australasian Triage Scale (ATS), 2, 34, 97
AVPU *See* alert, verbal, painful, unresponsive (AVPU)
alert, verbal, painful, unresponsive (AVPU), 8–10, 10*t*

B

benefits of Emergency Severity Index, 4
blood pressure (BP), 19, 33, 42, 97
BP. *See* blood pressure (BP)

C

Canadian Triage and Acuity Scale (CTAS), 1, 34, 97
clinical nurse specialist (CNS), 50, 51, 55, 60, 97
CNS. *See* clinical nurse specialist (CNS)
criteria, 2, 7, 9, 11–15, 17–22, 25, 27–29, 33–36
CTAS. See Canadian Triage and Acuity Scale (CTAS)

D

decision points, 7–13, 7*f*, 8*f*
  A, 9–11, 11*f*
  B, 11, 11*f*, 12, 18*f*
  C, 12, 13, 12*f*
  D, 13, 14*f*

E

ED. *See* emergency department (ED)
emergency department (ED), *viii*, 1, 97
Emergency Nurses Association (ENA), *viii*, 1, 22, 41, 49, 53, 97
ENA. *See* Emergency Nurses Association (ENA)
Emergency Severity Index (ESI), 1–37, 97
  benefits, 4
  criteria, 2, 7, 9, 11–15, 17–22, 25, 27–29, 33–36
  decision points, 7–13, 7*f*, 8*f*
    A, 9–11, 11g
    B, 11, 11*f*, 12
    C, 12, 12*f*, 13
    D, 13, 14*f*
  evaluation and quality improvement, 57–61
    data collection, 59–61
    quality indicators, 58, 59, 59*t*
  history, 2
  implementation, 49–55
    decision-making and planning, 49
    policies and procedures, 50
    education planning, 51
    post implementation, 55
  levels, 7, 8
    level 1, 9–11
    level 2, 11, 12, 17–25
    level 3, 13, 14, 27–31
    level 4, 13, 27–31
    level 5, 13, 27–31
  pediatric triage with ESI, 39–47
    assessment, 40–43
    background and research, 39
    differences from adult triage, 40
    levels, 43–46, 46*t*, 46*t*
    special populations, 45
    standardized approach, 41–43
  practice cases, 63–75
  research, 2–4
  resources, 2–4, 7, 8, 12–14, 13*t*, 14*t*, 22, 27–30, 33, 34, 43–45, 46*t*, 46*t*, 58
  resource needs, 2, 4, 7, 12, 13, 27, 28, 44
  standardization of triage acuity, 1, 2, 4
  triage algorithm, 8*f*, 27*f*, 95, 96
  vital signs, 7, 8, 13, 14, 14*f*, 40–44, 54, 97
    pediatric fever, 8, 14*f*, 35, 35*f*, 42, 96
  VS. *See* vital signs (VS)
Emergency Severity Index Conceptual Algorithm, v4, 7, 7*f*
ESI. *See* Emergency Severity Index (ESI)
ESI Triage Algorithm v4, 8*f*, 27*f*, 95, 96
evaluation and quality improvement (QI), 57–61
  quality indicators, 58, 59, 59*t*

data collection, 59–61

F

FAQ. *See* frequently asked questions (FAQ)
frequently asked questions (FAQ), 85–94

H

heart rate (HR) 13, 33, 97
history of the ESI, 2
HR. *See* heart rate (HR)implementation of ESI, 49–55
decision-making and planning, 49
policies and procedures, 50
education planning, 51
post implementation, 55
levels of the ESI, 7, 8
level 1, 9–11
level 2, 11, 12, 17–25
high-risk situation, 18–22
patient experiencing new onset confusion, lethargy, or disorientation, 22
patient expressing severe pain or distress, 22–25
level 3, 13, 14, 27–31
level 4, 13, 27–31
level 5, 13, 27–31

I

Institute of Medicine (IOM), 1, 58, 58*t*, 97
IOM. *See* Institute of Medicine (IOM)

O

oxygen saturation by pulse oximetry, 9, 13, 14, 14*t*, 18, 33, 33*t*, 35, 41, 42

P

PAT. *See* pediatric assessment triangle (PAT)
pediatric assessment triangle (PAT), 41, 87
pediatric triage with ESI
ABCDE. *See* Step 2. airway, breathing, circulation, disability, exposure/environmental control (ABCDE)
assessment, 40–43
assessment of rashes, 42
background and research, 39
differences from triage of adults, 40
infant triage, 42
levels, 43–46, 46*t*, 46*t*
pediatric assessment triangle, 41
resource considerations, 44
special populations, 45
standardized approach, 41–43
ABCDE. *See* Step 2. Airway, Breathing, Circulation, Disability, Exposure/Environmental Control, 41
Step 1. Appearance, Work of Breathing, Circulation – Quick Assessment, 41
Step 2. Airway, Breathing, Circulation, Disability, Exposure/Environmental Control, 41
Step 3. Pertinent History, 41
Step 4. Vital Signs, 41, 42
Step 5. Fever, 42
Step 6. Pain, 42
practice cases, 55, 63–75

Q

QI. *See* evaluation and quality improvement (QI)

R

research, 2–4
resources 2–4, 7, 8, 12–14, 13*t*, 14*t*, 22, 27–30, 28*t*, 33, 34, 43–45, 46*t*, 46*t*, 58
resource needs 2, 4, 7, 12–13, 27, 28, 44
respiratory rate (RR) 13, 33, 14*t*, 97
RR. *See* respiratory rate (RR)

S

SIRS. *See* systemic inflammatory response syndrome (SIRS)
SpO_2. *See* oxygen saturation by pulse oximetry (SpO_2.), 9, 13, 14, 14*f*, 18, 33, 33*f*, 35, 41, 42
standardization of triage acuity, 1, 2, 4
systemic inflammatory response syndrome (SIRS), 34, 97

T

T. *See* temperature (T)
temperature, 13, 29, 33, 35, 35*t*, 97
time to treatment, 14
triage algorithm, 8, 8*f*, 28, 28*f*
triage scales, 1, 2, 34
Australasian Triage Scale, 2, 34, 97
Canadian Triage and Acuity Scale, 1, 34, 97
Emergency Severity Index, 1–37, 97

V

vital signs, 7, 8, 13, 14, F 2-6, 40–44, 54, 97
pediatric fever 8, 14, 14*t*, 35, 35*t*, 42
